# Supplementary material for: Material composition and constitutive model development of red mud-based filler for highway tunnel invert filling applications: A comprehensive study
Source: PLoS One. 2025 Apr 16;20(4):e0321926. doi: 10.1371/journal.pone.0321926 (PMC12002488; doi:10.1371/journal.pone.0321926)
Supplement: S1 Table — Data of red mud emissions and comprehensive utilization rate. (DOCX) [file pone.0321926.s001.docx]

Table S1. Red mud emissions and comprehensive utilization rate in China in the past decade (Fig.1). Data of red mud emissions and comprehensive utilization rate.

| Years | Red mud discharge | Red mud reside | Comprehensive utilization | Comprehensive utilization rate |
| --- | --- | --- | --- | --- |
| 2011 | 4300 | 4077.26 | 222.74 | 5.18 |
| 2012 | 5800 | 5500.14 | 299.86 | 5.17 |
| 2013 | 7300 | 7010.19 | 289.81 | 3.97 |
| 2014 | 7500 | 7200 | 300 | 4 |
| 2015 | 8200 | 7899.88 | 300.12 | 3.66 |
| 2016 | 8400 | 8100.12 | 299.88 | 3.57 |
| 2017 | 10000 | 9600 | 400 | 4 |
| 2018 | 10500 | 10049.55 | 450.45 | 4.29 |
| 2019 | 10500 | 9650.55 | 849.45 | 8.09 |
| 2020 | 10620 | 9770.4 | 849.6 | 8 |
| 2021 | 10360 | 9759.12 | 600.88 | 5.8 |
| 2022 | 12280 | 11249 | 1031 | 8.4 |
| 2023 | 10700 | 9650 | 1050 | 9.4 |
