# Supplementary material for: Material composition and constitutive model development of red mud-based filler for highway tunnel invert filling applications: A comprehensive study
Source: PLoS One. 2025 Apr 16;20(4):e0321926. doi: 10.1371/journal.pone.0321926 (PMC12002488; doi:10.1371/journal.pone.0321926)
Supplement: S2 Table — Test results of compaction test. (DOCX) [file pone.0321926.s002.docx]

Table S2. Compaction test curves (Fig.5). Test results of compaction test.

| Dosage | 0% | 5% | 10% | 15% | 20% | 25% | 30% |
| --- | --- | --- | --- | --- | --- | --- | --- |
| Moisture content | 0.217 | 0.2016 | 0.20298 | 0.20309 | 0.16874 | 0.15421 | 0.14788 |
|  | 0.23432 | 0.23528 | 0.21148 | 0.21622 | 0.18828 | 0.18402 | 0.16368 |
|  | 0.2522 | 0.25651 | 0.23906 | 0.23821 | 0.20774 | 0.20337 | 0.18828 |
|  | 0.28529 | 0.2752 | 0.27544 | 0.26458 | 0.22558 | 0.22055 | 0.20646 |
|  | 0.30315 | 0.30117 | 0.31498 | 0.26794 | 0.25368 | 0.25244 | 0.22346 |
| Dry density | 1.81164 | 1.78464 | 1.77132 | 1.93498 | 1.81687 | 1.93317 | 1.95806 |
|  | 1.84203 | 1.90938 | 1.86988 | 1.94527 | 1.95727 | 1.98074 | 1.99398 |
|  | 1.85614 | 1.85789 | 1.89189 | 1.92193 | 1.92526 | 1.9556 | 1.95681 |
|  | 1.80377 | 1.84197 | 1.80898 | 1.83529 | 1.8965 | 1.91539 | 1.92299 |
|  | 1.7952 | 1.7586 | 1.72768 | 1.82561 | 1.85519 | 1.8937 | 1.88325 |
