# Supplementary material for: Material composition and constitutive model development of red mud-based filler for highway tunnel invert filling applications: A comprehensive study
Source: PLoS One. 2025 Apr 16;20(4):e0321926. doi: 10.1371/journal.pone.0321926 (PMC12002488; doi:10.1371/journal.pone.0321926)
Supplement: S3 Table — Test results of unconfined compressive strength. (DOCX) [file pone.0321926.s003.docx]

Table S3. Unconfined compressive strength of MRM (Fig.7). Test results of unconfined compressive strength.

| 7d | Compaction | 0% | 5% | 10% | 15% | 20% | 25% | 30% |
| --- | --- | --- | --- | --- | --- | --- | --- | --- |
|  | 96 | 0.54 | 1.76 | 2.09 | 2.82 | 3.36 | 3.93 | 4.49 |
|  | 93 | 0.43 | 1.32 | 1.57 | 2.14 | 2.85 | 3.27 | 3.77 |
|  | 90 | 0.35 | 1.04 | 1.25 | 1.69 | 2.45 | 2.78 | 3.25 |
| 28d | Compaction | 0% | 5% | 10% | 15% | 20% | 25% | 30% |
|  | 96 | 0.73 | 2.41 | 3.04 | 3.88 | 4.58 | 5.31 | 6.22 |
|  | 93 | 0.48 | 1.77 | 2.26 | 2.87 | 3.72 | 4.36 | 5.07 |
|  | 90 | 0.43 | 1.31 | 1.57 | 2.13 | 2.87 | 3.5 | 4.08 |
