# Supplementary material for: Material composition and constitutive model development of red mud-based filler for highway tunnel invert filling applications: A comprehensive study
Source: PLoS One. 2025 Apr 16;20(4):e0321926. doi: 10.1371/journal.pone.0321926 (PMC12002488; doi:10.1371/journal.pone.0321926)
Supplement: S4 Table — Data of the changing trend of unconfined compressive strength. (DOCX) [file pone.0321926.s004.docx]

Table S4. The changing trend of unconfined compressive strength of MRM (Fig.8). Data of the changing trend of unconfined compressive strength.

| 7d | Compaction | 0% | 5% | 10% | 15% | 20% | 25% | 30% |
| --- | --- | --- | --- | --- | --- | --- | --- | --- |
|  | 96 | 0% | 226% | 19% | 35% | 19% | 17% | 14% |
|  | 93 | 0% | 208% | 19% | 36% | 33% | 15% | 15% |
|  | 90 | 0% | 197% | 19% | 36% | 45% | 14% | 17% |
| 28d | Compaction | 0% | 5% | 10% | 15% | 20% | 25% | 30% |
|  | 96 | 0% | 232% | 26% | 28% | 18% | 16% | 17% |
|  | 93 | 0% | 263% | 28% | 27% | 30% | 17% | 16% |
|  | 90 | 0% | 205% | 24% | 31% | 35% | 22% | 17% |
