# Supplementary material for: Material composition and constitutive model development of red mud-based filler for highway tunnel invert filling applications: A comprehensive study
Source: PLoS One. 2025 Apr 16;20(4):e0321926. doi: 10.1371/journal.pone.0321926 (PMC12002488; doi:10.1371/journal.pone.0321926)
Supplement: S5 Table — Test results of unconfined compressive strength of MRM after adding soil stabilizers. (DOCX) [file pone.0321926.s005.docx]

Table S5. Unconfined compressive strength of MRM after adding soil stabilizers (Fig.9). Test results of unconfined compressive strength of MRM after adding soil stabilizers.

| Age | Type | Dilution multiple | UCS |
| --- | --- | --- | --- |
| 7d | TSD | 5 | 4.76 |
|  |  | 10 | 4.79 |
|  |  | 20 | 5.16 |
|  | SZLX | 10 | 4.55 |
|  |  | 20 | 4.97 |
|  |  | 30 | 4.71 |
| 28d | TSD | 5 | 7.37 |
|  |  | 10 | 6.66 |
|  |  | 20 | 6.58 |
|  | SZLX | 10 | 6.89 |
|  |  | 20 | 6.52 |
|  |  | 30 | 6.33 |
