# Supplementary material for: Material composition and constitutive model development of red mud-based filler for highway tunnel invert filling applications: A comprehensive study
Source: PLoS One. 2025 Apr 16;20(4):e0321926. doi: 10.1371/journal.pone.0321926 (PMC12002488; doi:10.1371/journal.pone.0321926)
Supplement: S6 Table — Test results of (σ1-σ3)-ε1 curves of RMBF with confining pressure under different ages. (DOCX) [file pone.0321926.s006.docx]

Table S6. The (σ_1_-σ_3_)-ε_1_ curves of RMBF with confining pressure under different ages (Fig.10). Test results of (σ_1_-σ_3_)-ε_1_ curves of RMBF with confining pressure under different ages.

(a) 7d

| 30kPa | | 60kPa | | 90kPa | |
| --- | --- | --- | --- | --- | --- |
| ε_1_ | (σ_1_-σ_3_) | ε_1_ | (σ_1_-σ_3_) | ε_1_ | (σ_1_-σ_3_) |
| 0 | 0.2223 | 0.0017 | -2.9 | 0 | 1.3112 |
| 0.0019 | 0.4645 | 0 | -1.6 | 0.0058 | 1.0567 |
| 0.0019 | 0.2871 | 0 | -2.6 | 0.0095 | 1.0475 |
| 0.0019 | 0.5099 | 0 | 1.1 | 0.0095 | 0.8638 |
| 0.0019 | 0.722 | -0.0019 | 1.8 | 0.0132 | 0.2591 |
| 0.0076 | 0.529 | -0.0019 | 4.4 | 0.0114 | 1.3179 |
| 0.0095 | 1.0034 | -0.0019 | 5.4 | 0.0132 | 0.5444 |
| 0.0132 | 0.9784 | -0.0019 | 1.7 | 0.0171 | 1.832 |
| 0.0209 | 1.2021 | -0.0019 | 0.8 | 0.0171 | 1.0465 |
| 0.0265 | 1.8452 | 0.0039 | 1.8 | 0.0209 | 1.8114 |
| 0.0285 | 1.4944 | 0.0058 | 0.7 | 0.0228 | 1.3142 |
| 0.0304 | 2.04 | 0.0058 | 2 | 0.0228 | 1.8284 |
| 0.0304 | 1.4499 | 0.0115 | 1.6 | 0.0247 | 1.7778 |
| 0.0304 | 1.4987 | 0.0229 | 12.5 | 0.0285 | 2.6579 |
| 0.0323 | 1.7994 | 0.0343 | 13.6 | 0.0323 | 2.4017 |
| 0.0323 | 1.7783 | 0.0438 | 3.7 | 0.0361 | 3.6283 |
| 0.0341 | 2.5738 | 0.0551 | 4.4 | 0.0361 | 1.8266 |
| 0.0341 | 2.3067 | 0.0589 | 4.6 | 0.0417 | 2.8958 |
| 0.0341 | 2.0983 | 0.0626 | 5.4 | 0.0436 | 3.3848 |
| 0.0379 | 2.57 | 0.076 | 4.6 | 0.0475 | 2.5924 |
| 0.0379 | 2.3014 | 0.0969 | 4.6 | 0.0513 | 3.3237 |
| 0.0417 | 2.5151 | 0.1252 | 4.4 | 0.0531 | 2.6494 |
| 0.0455 | 2.8081 | 0.1575 | 4.2 | 0.0531 | 2.3889 |
| 0.0455 | 2.7855 | 0.1633 | 2.6 | 0.057 | 2.8622 |
| 0.0455 | 3.0006 | 0.1784 | 3.9 | 0.0589 | 2.8431 |
| 0.0494 | 3.047 | 0.1898 | 3.5 | 0.0607 | 2.8904 |
| 0.0494 | 3.0164 | 0.1974 | 2.4 | 0.0607 | 2.6435 |
| 0.0513 | 2.9975 | 0.2164 | 2.5 | 0.0589 | 3.111 |
| 0.055 | 3.2998 | 0.2259 | 2.6 | 0.0607 | 3.3418 |
| 0.0587 | 4.0833 | 0.2354 | 2.7 | 0.0626 | 3.0714 |
| 0.0626 | 3.8775 | 0.2543 | 3.4 | 0.0645 | 3.1013 |
| 0.0645 | 4.0868 | 0.2656 | 2.5 | 0.0626 | 3.1191 |
| 0.0626 | 3.2563 | 0.2733 | 0.7 | 0.0645 | 3.8196 |
| 0.0645 | 3.2477 | 0.2885 | -0.5 | 0.0645 | 3.5565 |
| 0.0645 | 3.8123 | 0.3055 | -0.6 | 0.0645 | 3.6241 |
| 0.0645 | 4.6257 | 0.3131 | 0.5 | 0.0645 | 3.057 |
| 0.0645 | 4.3739 | 0.3169 | -0.2 | 0.0645 | 3.5495 |
| 0.0664 | 4.3695 | 0.3187 | 2.5873 | 0.0645 | 3.7987 |
| 0.2485 | 8.4947 | 0.4706 | 8.686 | 0.2524 | 11.0757 |
| 0.4591 | 10.2549 | 0.7173 | 9.5087 | 0.4364 | 16.1269 |
| 0.6679 | 13.2873 | 0.9051 | 9.1073 | 0.666 | 25.7951 |
| 0.85 | 15.8624 | 1.1327 | 11.1981 | 0.8632 | 326.7377 |
| 1.1043 | 17.8062 | 1.313 | 15.8558 | 1.0815 | 949.7297 |
| 1.2769 | 17.0807 | 1.5292 | 18.5962 | 1.2731 | 1418.855 |
| 1.4838 | 11.3609 | 1.7494 | 19.645 | 1.4951 | 2159.722 |
| 1.6924 | 13.7066 | 1.9429 | 25.8746 | 1.6868 | 2649.728 |
| 1.903 | 15.6408 | 2.146 | 29.5913 | 1.8955 | 3019.257 |
| 2.0832 | 12.5403 | 2.3831 | 30.9294 | 2.1023 | 3121.762 |
| 2.3394 | 16.0364 | 2.5064 | 32.0954 | 2.328 | 3332.711 |
| 2.5537 | 15.5548 | 2.7986 | 37.6391 | 2.5405 | 3273.218 |
| 2.7492 | 15.7249 | 2.9751 | 38.4801 | 2.7492 | 3186.74 |
| 2.9731 | 15.9515 | 3.1933 | 48.4347 | 2.9655 | 2608.461 |
| 3.142 | 10.6958 | 3.4001 | 117.0024 | 3.1439 | 2253.418 |
| 3.3905 | 19.901 | 3.6335 | 355.0367 | 3.3659 | 2021.34 |
| 3.584 | 21.3216 | 3.8212 | 668.6667 | 3.5728 | 1828.382 |
| 3.7851 | 156.8221 | 4.0376 | 1232.543 | 3.7852 | 1725.934 |
| 3.9901 | 545.8233 | 4.2463 | 1640.912 | 3.9977 | 1631.305 |
| 4.1987 | 964.5913 | 4.4587 | 1952.761 | 4.1894 | 1587.658 |
| 4.3999 | 1365.265 | 4.6524 | 1815.354 | 4.398 | 1514.22 |
| 4.6105 | 1929.011 | 4.8781 | 1752.615 | 4.6371 | 1514.446 |
| 4.804 | 2323.615 | 5.0565 | 1818.893 | 4.8135 | 1489.714 |
| 5.0165 | 2665.212 | 5.2614 | 2005.802 | 5.0318 | 1422.125 |
| 5.2404 | 2434.871 | 5.4795 | 2034.141 | 5.231 | 1406.944 |
| 5.4663 | 2266.914 | 5.694 | 2159.336 | 5.453 | 1390.338 |
| 5.6862 | 1998.134 | 5.8931 | 2405.247 | 5.6465 | 1388.651 |
| 5.8627 | 1761.524 | 6.1019 | 2495.41 | 5.8627 | 1370.263 |
| 6.0809 | 1761.599 | 6.3257 | 2626.206 | 6.0771 | 1344.885 |
| 6.2859 | 1731.373 | 6.5401 | 2517.142 | 6.2745 | 1329.76 |
| 6.4965 | 1820.593 | 6.7489 | 1794.179 | 6.4965 | 1358.022 |
| 6.709 | 1840.739 | 6.9633 | 1548.477 | 6.7128 | 1324.206 |
| 6.9195 | 1859.979 | 7.1758 | 1431.955 | 6.9006 | 1282.871 |
| 7.1206 | 2002.567 | 7.3845 | 1355.37 | 7.1113 | 1268.86 |
| 7.3674 | 2054.493 | 7.5989 | 1271.44 | 7.3161 | 1266.526 |
| 7.5362 | 2253.33 | 7.8076 | 1223.05 | 7.5286 | 1267.422 |
| 7.7468 | 2379.378 | 7.9859 | 1191.177 | 7.7487 | 1288.738 |
| 7.9631 | 2507.972 | 8.2023 | 1121.496 | 7.9555 | 1253.599 |
| 8.1529 | 2607.098 | 8.4299 | 1096.232 | 8.17 | 1244.712 |
| 8.3767 | 2713.727 | 8.6367 | 1086.439 | 8.3654 | 1217.068 |
| 8.5816 | 1963.96 | 8.8321 | 1058.798 | 8.5911 | 1214.132 |
| 8.796 | 1620.914 | 9.0446 | 1036.093 | 8.7923 | 1232.289 |
| 9.0085 | 1169.746 | 9.1984 | 1052.246 | 8.9991 | 1182.296 |
| 9.204 | 1108.407 | 9.4506 | 1005.583 | 9.223 | 1213.497 |
| 9.3975 | 1030.489 | 9.6745 | 966.021 | 9.4165 | 1204.164 |
| 9.6119 | 1072.265 | 9.8719 | 977.322 | 9.6005 | 1167.345 |
| 9.8186 | 988.4766 | 10.0881 | 932.861 | 9.8282 | 1215.217 |
| 10.0274 | 973.8125 | 10.2874 | 947.08 | 10.0482 | 1244.283 |
| 10.2456 | 993.2987 | 10.4922 | 920.375 | 10.2305 | 1177.041 |
| 10.441 | 982.9313 | 10.6954 | 919.4552 | 10.4544 | 1199.027 |
| 10.6593 | 969.9158 | 10.9267 | 910.7917 | 10.6669 | 1156.771 |
| 10.8792 | 1023.627 | 11.1051 | 926.7781 | 10.866 | 1134.085 |
| 11.0822 | 1005.787 | 11.3443 | 933.111 | 11.0729 | 1121.87 |
| 11.2986 | 988.2377 | 11.551 | 858.1426 | 11.2721 | 1110.011 |
| 11.4979 | 984.6534 | 11.7465 | 870.2026 | 11.5016 | 1147.783 |
| 11.6875 | 971.6887 | 11.9609 | 850.5466 | 11.7218 | 1097.344 |
| 11.9209 | 980.542 | 12.1676 | 843.441 | 11.8983 | 1103.343 |
| 12.124 | 976.6729 | 12.3555 | 898.0973 | 12.1277 | 1118.975 |
| 12.3289 | 979.4103 | 12.5812 | 838.0986 | 12.3119 | 1116.081 |
| 12.5281 | 941.0792 | 12.7937 | 828.8518 | 12.5433 | 1113.198 |
| 12.7595 | 940.363 | 13.0062 | 823.3415 | 12.7501 | 1110.81 |
| 12.9474 | 921.6833 | 13.2074 | 866.9999 | 12.938 | 1087.26 |
| 13.1485 | 909.1389 | 13.4047 | 864.9917 | 13.1694 | 1091.519 |
| 13.3572 | 888.6478 | 13.6096 | 809.335 | 13.3744 | 1097.862 |
| 13.5849 | 887.9634 | 13.824 | 809.2016 | 13.5869 | 1083.486 |
| 13.7936 | 873.3112 | 14.0309 | 808.7418 | 13.7917 | 1092.584 |
| 14.008 | 877.6036 | 14.2566 | 811.7732 | 14.0024 | 1073.637 |
| 14.211 | 855.8958 | 14.4464 | 847.1695 | 14.2015 | 1042.19 |
| 14.3989 | 841.7752 | 14.655 | 797.9464 | 14.4026 | 1047.138 |
| 14.6114 | 837.9693 | 14.8732 | 845.0461 | 14.6133 | 1049.937 |
| 14.8144 | 827.2611 | 14.9937 | 784.4625 | 14.8296 | 1037.096 |

(b) 14d

| 30kPa | | 60kPa | | 90kPa | |
| --- | --- | --- | --- | --- | --- |
| ε_1_ | (σ_1_-σ_3_) | ε_1_ | (σ_1_-σ_3_) | ε_1_ | (σ_1_-σ_3_) |
| 0 | 1.0304 | 0.002 | 1.6009 | -0.0019 | 3.1325 |
| 0.002 | 1.023 | 0 | 1.6061 | 0 | 3.3791 |
| 0.0039 | 1.0153 | 0 | 1.8524 | 0 | 2.954 |
| 0.0039 | 1.0011 | 0 | 1.5948 | -0.0019 | 2.6931 |
| 0.0039 | 0.9903 | -0.0019 | 1.5878 | -0.0019 | 2.9262 |
| 0.0039 | 0.9777 | -0.0019 | 1.5758 | -0.0039 | 2.6569 |
| 0.0076 | 0.9594 | -0.0019 | 1.8095 | -0.0039 | 2.6387 |
| 0.0076 | 1.8535 | -0.0019 | 1.5408 | -0.0058 | 2.868 |
| 0.0114 | 2.0789 | -0.0019 | 1.6073 | -0.0076 | 2.9278 |
| 0.0134 | 1.8114 | 0.0039 | 1.8326 | -0.0076 | 2.9045 |
| 0.0134 | 1.2086 | 0.0058 | 1.565 | -0.0058 | 3.1231 |
| 0.0152 | 2.1021 | 0.0058 | 1.5424 | -0.0076 | 3.1773 |
| 0.0152 | 2.0779 | 0.0115 | 1.5972 | -0.0076 | 3.1505 |
| 0.019 | 1.8005 | 0.0229 | 1.814 | -0.0114 | 3.1154 |
| 0.0247 | 2.5236 | 0.0343 | 1.6112 | -0.0114 | 3.4277 |
| 0.0266 | 2.0728 | 0.0438 | 1.8302 | -0.0152 | 3.3857 |
| 0.0266 | 2.3024 | 0.0551 | 1.7909 | -0.0152 | 3.6856 |
| 0.0285 | 2.5288 | 0.0589 | 1.8425 | -0.038 | 3.4154 |
| 0.0323 | 2.8222 | 0.0626 | 1.8064 | -0.0417 | 3.3846 |
| 0.0323 | 2.302 | 0.076 | 1.8499 | -0.0513 | 3.1869 |
| 0.0343 | 2.3416 | 0.0969 | 1.7966 | -0.0531 | 3.394 |
| 0.0343 | 2.5696 | 0.1252 | 1.8207 | -0.055 | 3.3537 |
| 0.038 | 3.0332 | 0.1575 | 2.6717 | -0.0607 | 3.4001 |
| 0.0417 | 3.4093 | 0.1633 | 2.6217 | -0.0626 | 3.4299 |
| 0.0417 | 3.0612 | 0.1784 | 2.6517 | -0.0664 | 3.6393 |
| 0.0475 | 3.3527 | 0.1898 | 2.8443 | -0.0684 | 3.6769 |
| 0.0513 | 3.3311 | 0.1974 | 2.8806 | -0.0703 | 3.6271 |
| 0.0531 | 2.802 | 0.2164 | 2.8929 | -0.0759 | 3.668 |
| 0.0531 | 3.5882 | 0.2259 | 2.8501 | -0.0759 | 3.6051 |
| 0.0531 | 3.3892 | 0.2354 | 2.8726 | -0.0759 | 3.8863 |
| 0.057 | 3.3646 | 0.2543 | 3.1332 | -0.0759 | 3.9106 |
| 0.0645 | 3.8197 | 0.2656 | 3.0856 | -0.0759 | 3.8803 |
| 0.0645 | 3.889 | 0.2733 | 3.3829 | -0.0759 | 3.8478 |
| 0.0721 | 4.116 | 0.2885 | 3.3522 | -0.0759 | 4.2417 |
| 0.076 | 4.1 | 0.3055 | 3.6432 | -0.0759 | 3.8951 |
| 0.0779 | 3.3481 | 0.3131 | 3.6061 | -0.0759 | 4.4516 |
| 0.0797 | 4.0725 | 0.3169 | 3.8462 | -0.0759 | 4.1852 |
| 0.0816 | 4.0604 | 0.3187 | 3.5708 | -0.0759 | 4.1746 |
| 0.2619 | 6.3666 | 0.4706 | 5.3761 | 0.1139 | 11.7253 |
| 0.4857 | 8.9729 | 0.7173 | 6.6807 | 0.3149 | 17.9279 |
| 0.666 | 8.6852 | 0.9051 | 7.3892 | 0.5141 | 25.1195 |
| 0.8899 | 9.6688 | 1.1327 | 8.2228 | 0.7285 | 32.9787 |
| 1.1024 | 8.8705 | 1.313 | 9.2877 | 0.9467 | 35.9758 |
| 1.2902 | 9.5788 | 1.5292 | 9.9652 | 1.1574 | 41.0068 |
| 1.499 | 10.1284 | 1.7494 | 11.7296 | 1.3395 | 43.8018 |
| 1.7057 | 10.855 | 1.9429 | 13.7064 | 1.5615 | 58.9569 |
| 1.9373 | 12.6266 | 2.146 | 16.2531 | 1.7759 | 67.5242 |
| 2.127 | 12.8078 | 2.3831 | 19.9732 | 1.9789 | 258.3495 |
| 2.3319 | 13.8547 | 2.5064 | 20.9853 | 2.1705 | 687.0336 |
| 2.5576 | 14.7602 | 2.7986 | 25.1302 | 2.3679 | 1146.917 |
| 2.7759 | 17.7381 | 2.9751 | 25.911 | 2.5785 | 1732.755 |
| 2.9789 | 18.4505 | 3.1933 | 29.7835 | 2.8175 | 2291.632 |
| 3.1933 | 23.1805 | 3.4001 | 31.98 | 2.9864 | 1917.004 |
| 3.3981 | 28.1347 | 3.6335 | 40.3847 | 3.2216 | 2327.756 |
| 3.5803 | 35.9199 | 3.8212 | 302.2815 | 3.419 | 2473.852 |
| 3.8365 | 299.2428 | 4.0376 | 646.3006 | 3.6068 | 2582.131 |
| 4.0167 | 600.308 | 4.2463 | 1229.138 | 3.8535 | 3157.833 |
| 4.2197 | 1100.454 | 4.4587 | 1619.373 | 4.0602 | 3176.754 |
| 4.436 | 1578.364 | 4.6524 | 1974.299 | 4.2785 | 3040.762 |
| 4.6295 | 1979.727 | 4.8781 | 1603.35 | 4.4834 | 2785.726 |
| 4.8325 | 1694.502 | 5.0565 | 1535.121 | 4.6788 | 2395.65 |
| 5.0564 | 1588.243 | 5.2614 | 1571.243 | 4.8856 | 2098.748 |
| 5.2556 | 1443.643 | 5.4795 | 1981.183 | 5.0944 | 1876.288 |
| 5.4739 | 1543.471 | 5.694 | 1933.302 | 5.3125 | 1718.675 |
| 5.6825 | 1587.512 | 5.8931 | 2149.109 | 5.5308 | 1628.075 |
| 5.8627 | 1673.777 | 6.1019 | 2310.782 | 5.7394 | 1615.035 |
| 6.0848 | 1813.448 | 6.3257 | 2462.473 | 5.9462 | 1518.77 |
| 6.2916 | 1968.827 | 6.5401 | 2566.212 | 6.1454 | 1470.85 |
| 6.5136 | 2140.241 | 6.7489 | 2418.891 | 6.3428 | 1452.062 |
| 6.7222 | 2263.012 | 6.9633 | 1640.502 | 6.559 | 1392.677 |
| 6.9386 | 2125.224 | 7.1758 | 1243.453 | 6.7582 | 1403.942 |
| 7.134 | 1312.562 | 7.3845 | 1217.231 | 6.9651 | 1353.932 |
| 7.3256 | 1066.736 | 7.5989 | 1068.196 | 7.1681 | 1334.586 |
| 7.5534 | 897.815 | 7.8076 | 968.0883 | 7.3616 | 1300.688 |
| 7.7639 | 744.9958 | 7.9859 | 895.0682 | 7.595 | 1317.61 |
| 7.9897 | 670.5224 | 8.2023 | 851.7544 | 7.8207 | 1296.385 |
| 8.1719 | 606.1983 | 8.4299 | 849.2334 | 8.0067 | 1258.846 |
| 8.3767 | 586.6986 | 8.6367 | 819.8307 | 8.2344 | 1296.648 |
| 8.595 | 605.0813 | 8.8321 | 791.2851 | 8.4487 | 1270.54 |
| 8.8094 | 579.4281 | 9.0446 | 837.0536 | 8.6518 | 1221.409 |
| 9.001 | 559.0399 | 9.1984 | 647.6346 | 8.8377 | 1231.929 |
| 9.2249 | 564.6395 | 9.4506 | 815.7448 | 9.0408 | 1213.269 |
| 9.4165 | 556.5923 | 9.6745 | 784.3209 | 9.259 | 1214.004 |
| 9.6499 | 573.9183 | 9.8719 | 834.3031 | 9.4544 | 1215.192 |
| 9.8586 | 588.2851 | 10.0881 | 760.9339 | 9.6764 | 1226.715 |
| 10.0369 | 588.3332 | 10.2874 | 739.6935 | 9.904 | 1251.647 |
| 10.2532 | 597.9993 | 10.4922 | 804.8147 | 10.109 | 1231.121 |
| 10.4734 | 626.8249 | 10.6954 | 736.7262 | 10.3157 | 1191.469 |
| 10.6915 | 620.6449 | 10.9267 | 731.5478 | 10.5245 | 1236.874 |
| 10.8775 | 616.9363 | 11.1051 | 719.8544 | 10.7331 | 1182.853 |
| 11.1014 | 655.571 | 11.3443 | 725.7053 | 10.9476 | 1177.045 |
| 11.3005 | 693.457 | 11.551 | 748.3251 | 11.1468 | 1122.512 |
| 11.5111 | 658.2561 | 11.7465 | 763.1221 | 11.3346 | 1168.135 |
| 11.7122 | 618.9873 | 11.9609 | 783.2719 | 11.5661 | 1155.257 |
| 11.8944 | 609.3286 | 12.1676 | 807.6954 | 11.7673 | 1169.9 |
| 12.124 | 609.2994 | 12.3555 | 796.6335 | 11.9684 | 1184.934 |
| 12.3497 | 629.5725 | 12.5812 | 784.6365 | 12.1751 | 1112.953 |
| 12.5548 | 624.7972 | 12.7937 | 821.6664 | 12.3801 | 1125.442 |
| 12.7653 | 606.239 | 13.0062 | 836.0942 | 12.6172 | 1114.969 |
| 12.9589 | 608.5706 | 13.2074 | 811.2025 | 12.8108 | 1114.395 |
| 13.1809 | 630.5669 | 13.4047 | 828.1275 | 13.0271 | 1141.001 |
| 13.3744 | 607.0918 | 13.6096 | 849.5778 | 13.2358 | 1130.347 |
| 13.6039 | 598.1145 | 13.824 | 833.6161 | 13.4218 | 1123.355 |
| 13.7994 | 595.2533 | 14.0309 | 855.3158 | 13.6531 | 1122.92 |
| 14.0194 | 614.8607 | 14.2566 | 883.047 | 13.8637 | 1086.735 |
| 14.2091 | 607.4713 | 14.4464 | 866.4523 | 14.0667 | 1023.821 |
| 14.4292 | 623.4812 | 14.655 | 881.6311 | 14.2736 | 1040.818 |
| 14.6436 | 629.3671 | 14.8732 | 933.3688 | 14.4804 | 992.1158 |
| 14.8296 | 627.439 |  |  | 14.691 | 995.8989 |

(c) 28d

| 30kPa | | 60kPa | | 90kPa | |
| --- | --- | --- | --- | --- | --- |
| ε_1_ | (σ_1_-σ_3_) | ε_1_ | (σ_1_-σ_3_) | ε_1_ | (σ_1_-σ_3_) |
| 0 | 2.3863 | -0.0019 | 1.3017 | 0 | 1.3703 |
| 0.0038 | 2.125 | 0 | 1.2959 | 0 | 1.6147 |
| 0.0038 | 1.6045 | 0 | 1.1177 | 0.0019 | 1.3576 |
| 0.0038 | 1.5826 | -0.0019 | 1.3579 | 0.0038 | 1.5937 |
| 0.0056 | 1.5547 | -0.0019 | 1.099 | 0.0095 | 1.5869 |
| 0.0095 | 2.4423 | -0.0019 | 1.086 | 0.0114 | 1.3285 |
| 0.0095 | 1.5839 | -0.0019 | 1.0699 | 0.0228 | 1.312 |
| 0.0095 | 2.1375 | -0.0019 | 1.048 | 0.0265 | 1.5495 |
| 0.0132 | 2.4274 | 0.0019 | 1.3569 | 0.036 | 1.2915 |
| 0.0114 | 2.145 | 0.0075 | 1.3266 | 0.0455 | 1.6048 |
| 0.017 | 2.6174 | 0.0114 | 1.2872 | 0.0664 | 1.5926 |
| 0.019 | 2.4142 | 0.0228 | 1.327 | 0.0759 | 1.5728 |
| 0.019 | 2.1278 | 0.0323 | 1.3668 | 0.0796 | 1.5641 |
| 0.019 | 2.1691 | 0.0398 | 1.3267 | 0.0854 | 1.548 |
| 0.0228 | 2.6289 | 0.0436 | 1.3606 | 0.0967 | 1.6158 |
| 0.0265 | 2.9151 | 0.0569 | 1.3147 | 0.1195 | 1.8483 |
| 0.0265 | 2.6267 | 0.0721 | 1.3416 | 0.1404 | 1.8265 |
| 0.0265 | 2.6753 | 0.091 | 1.2894 | 0.1574 | 1.8064 |
| 0.0284 | 2.8709 | 0.11 | 1.302 | 0.1745 | 1.7925 |
| 0.0304 | 2.6604 | 0.129 | 1.3158 | 0.203 | 1.8469 |
| 0.0341 | 3.1156 | 0.165 | 1.5598 | 0.222 | 2.0713 |
| 0.0398 | 2.9045 | 0.184 | 1.5714 | 0.2448 | 2.046 |
| 0.0474 | 3.1655 | 0.2106 | 1.809 | 0.2561 | 2.6097 |
| 0.055 | 3.3691 | 0.239 | 1.7969 | 0.2731 | 2.6685 |
| 0.0569 | 3.1365 | 0.2599 | 1.7966 | 0.2845 | 2.8931 |
| 0.0569 | 3.0936 | 0.2846 | 2.0435 | 0.3016 | 2.8756 |
| 0.0587 | 2.8837 | 0.2979 | 2.6142 | 0.3035 | 2.8581 |
| 0.0626 | 3.4014 | 0.3187 | 2.6423 | 0.3244 | 3.17 |
| 0.0683 | 3.3342 | 0.3357 | 2.6401 | 0.3396 | 3.3919 |
| 0.0721 | 3.3689 | 0.3471 | 2.6448 | 0.3415 | 3.3721 |
| 0.0777 | 3.397 | 0.3661 | 2.8842 | 0.3452 | 3.1037 |
| 0.0854 | 3.6759 | 0.3851 | 3.0908 | 0.3585 | 3.4203 |
| 0.0949 | 3.7117 | 0.3984 | 3.1234 | 0.3661 | 3.658 |
| 0.0986 | 3.6863 | 0.406 | 3.3388 | 0.3756 | 3.6495 |
| 0.0986 | 3.6674 | 0.4136 | 3.3959 | 0.3851 | 3.1434 |
| 0.1024 | 3.9685 | 0.4211 | 3.3722 | 0.3965 | 3.3663 |
| 0.1081 | 3.6985 | 0.4306 | 3.8336 | 0.4098 | 3.3668 |
| 0.1081 | 3.6873 | 0.4345 | 3.8209 | 0.4174 | 3.347 |
| 0.294 | 5.986 | 0.6204 | 5.9946 | 0.5939 | 4.2182 |
| 0.499 | 8.2087 | 0.8064 | 7.168 | 0.8139 | 5.1404 |
| 0.6981 | 10.0073 | 1.0378 | 10.0282 | 1.0189 | 6.4884 |
| 0.8936 | 10.9981 | 1.237 | 11.7684 | 1.2199 | 142.1022 |
| 1.1308 | 14.5558 | 1.459 | 14.8222 | 1.421 | 576.6523 |
| 1.3186 | 17.0962 | 1.6659 | 17.904 | 1.6544 | 1132.911 |
| 1.5444 | 19.345 | 1.8708 | 21.116 | 1.8214 | 1716.895 |
| 1.755 | 24.3746 | 2.0795 | 26.2019 | 2.0452 | 2461.915 |
| 1.9637 | 29.0913 | 2.29 | 32.7498 | 2.2426 | 2907.933 |
| 2.1515 | 30.3294 | 2.4987 | 36.1525 | 2.4665 | 3211.493 |
| 2.383 | 29.1954 | 2.6847 | 40.5371 | 2.6733 | 3097.94 |
| 2.5804 | 35.4391 | 2.8973 | 55.0691 | 2.8896 | 2939.365 |
| 2.7795 | 37.0801 | 3.1116 | 441.9048 | 3.1003 | 2725.334 |
| 3.0054 | 47.2347 | 3.3241 | 959.0404 | 3.3033 | 2446.426 |
| 3.1989 | 117.6024 | 3.5271 | 1580.079 | 3.5232 | 2436.393 |
| 3.4 | 356.4367 | 3.7282 | 2076.149 | 3.7093 | 2277.601 |
| 3.5993 | 668.5667 | 3.9389 | 2522.542 | 3.9672 | 2385.434 |
| 3.8364 | 1231.843 | 4.1723 | 2488.052 | 4.1438 | 2167.502 |
| 4.0431 | 1642.212 | 4.379 | 2563.899 | 4.3581 | 2109.795 |
| 4.2481 | 1951.761 | 4.582 | 2588.554 | 4.5555 | 2058.538 |
| 4.415 | 1812.754 | 4.7546 | 2711.104 | 4.7641 | 2019.611 |
| 4.6731 | 1752.415 | 4.9994 | 2827.313 | 4.9729 | 1911.235 |
| 4.8534 | 1818.293 | 5.1968 | 2933.705 | 5.1796 | 1952.163 |
| 5.081 | 2005.302 | 5.4111 | 2918.058 | 5.3941 | 1849.068 |
| 5.2745 | 2033.341 | 5.6161 | 2806.179 | 5.6028 | 1844.128 |
| 5.4851 | 2160.136 | 5.8324 | 2226.466 | 5.766 | 1772.142 |
| 5.709 | 2406.247 | 6.0411 | 1767.695 | 6.0164 | 1708.96 |
| 5.912 | 2496.51 | 6.246 | 1539.991 | 6.2326 | 1716.382 |
| 6.0961 | 2627.306 | 6.4509 | 1401.17 | 6.4395 | 1650.177 |
| 6.3256 | 2515.942 | 6.6596 | 1302.197 | 6.6293 | 1540.215 |
| 6.5571 | 1787.279 | 6.8759 | 1210.674 | 6.8474 | 1583.536 |
| 6.762 | 1545.177 | 7.0751 | 1167.388 | 7.0447 | 1523.341 |
| 6.9594 | 1432.955 | 7.2932 | 1134.111 | 7.2763 | 1487.13 |
| 7.1681 | 1355.27 | 7.5001 | 1105.684 | 7.4679 | 1391.978 |
| 7.3598 | 1272.24 | 7.6955 | 1066.531 | 7.6727 | 1388.065 |
| 7.5855 | 1222.35 | 7.8871 | 1059.455 | 7.8625 | 1364.701 |
| 7.7943 | 1189.177 | 8.1091 | 1056.487 | 8.0655 | 1354.656 |
| 7.9782 | 1121.896 | 8.3274 | 1036.97 | 8.3008 | 1328.651 |
| 8.1984 | 1094.032 | 8.5361 | 1056.514 | 8.4981 | 1354.299 |
| 8.3995 | 1085.039 | 8.741 | 1034.55 | 8.7239 | 1345.511 |
| 8.6234 | 1056.498 | 8.9496 | 1062.967 | 8.9231 | 1293.73 |
| 8.8396 | 1033.893 | 9.1584 | 993.3336 | 9.1413 | 1333.14 |
| 9.0426 | 1048.446 | 9.369 | 981.3033 | 9.3537 | 1329.16 |
| 9.2476 | 1004.083 | 9.5796 | 1016.832 | 9.5398 | 1275.443 |
| 9.4411 | 965.8206 | 9.7599 | 975.9506 | 9.7485 | 1301.342 |
| 9.6669 | 975.4225 | 9.9989 | 969.4088 | 9.9515 | 1261.006 |
| 9.8851 | 931.1614 | 10.2076 | 965.0977 | 10.1791 | 1289.975 |
| 10.0919 | 945.48 | 10.3879 | 959.9457 | 10.3898 | 1265.215 |
| 10.2779 | 918.675 | 10.6099 | 954.5981 | 10.5776 | 1209.477 |
| 10.4827 | 917.8552 | 10.8375 | 950.6943 | 10.7901 | 1216.677 |
| 10.6971 | 909.1918 | 11.0178 | 951.3979 | 11.0064 | 1190.714 |
| 10.9134 | 924.7781 | 11.2265 | 956.4624 | 11.2207 | 1179.967 |
| 11.1221 | 932.8106 | 11.458 | 958.392 | 11.4295 | 1163.268 |
| 11.3365 | 856.6426 | 11.6629 | 997.9225 | 11.6288 | 1168.571 |
| 11.5339 | 869.7026 | 11.8621 | 925.3562 | 11.8299 | 1158.262 |
| 11.754 | 850.0466 | 12.0784 | 943.7734 | 12.0499 | 1127.235 |
| 11.9436 | 843.041 | 12.2927 | 939.1102 | 12.2396 | 1102.103 |
| 12.1619 | 897.5973 | 12.4958 | 924.8019 | 12.4598 | 1138.992 |
| 12.3763 | 836.6986 | 12.7045 | 935.514 | 12.6704 | 1140.094 |
| 12.5793 | 828.6518 | 12.8999 | 914.7972 | 12.8771 | 1108.568 |
| 12.7785 | 824.1415 | 13.1219 | 921.4097 | 13.0877 | 1113.569 |
| 12.9986 | 865.5999 | 13.3211 | 928.7355 | 13.306 | 1123.665 |
| 13.2072 | 862.4917 | 13.528 | 954.882 | 13.5185 | 1069.23 |
| 13.4028 | 808.335 | 13.7215 | 926.889 | 13.7234 | 1112.954 |
| 13.6229 | 808.5016 | 13.9624 | 931.6219 | 13.9226 | 1058.917 |
| 13.8391 | 807.2418 | 14.1655 | 948.8654 | 14.1351 | 1034.16 |
| 14.0213 | 811.7732 | 14.3741 | 950.3435 | 14.3533 | 1076.351 |
| 14.2509 | 845.2695 | 14.5866 | 979.376 | 14.5676 | 1070.5 |
| 14.4539 | 796.1464 | 14.7669 | 966.6097 | 14.765 | 1084.779 |
| 14.6606 | 842.7461 | 14.9965 | 983.783 | 14.9717 | 1024.898 |
| 14.8617 | 782.3625 |  |  |  |  |
