# Supplementary material for: Material composition and constitutive model development of red mud-based filler for highway tunnel invert filling applications: A comprehensive study
Source: PLoS One. 2025 Apr 16;20(4):e0321926. doi: 10.1371/journal.pone.0321926 (PMC12002488; doi:10.1371/journal.pone.0321926)
Supplement: S7 Table — Test results of The changing trend of E and (σ1-σ3)f of RMBF. (DOCX) [file pone.0321926.s007.docx]

Table S7. The changing trend of *E* and (σ_1_-σ_3_)_f_ of RMBF (Fig.12). Test results of The changing trend of *E* and (σ_1_-σ_3_)_f_ of RMBF.

(a) E -age

| 7d | 30kPa | 2.03 |
| --- | --- | --- |
|  | 60kPa | 2.02 |
|  | 90kPa | 2.43 |
| 14d | 30kPa | 1.9 |
|  | 60kPa | 1.9 |
|  | 90kPa | 2.14 |
| 28d | 30kPa | 1.75 |
|  | 60kPa | 2.37 |
|  | 90kPa | 2.5 |

(b) E -confining pressure

| 30kPa | 7d | 2.03 |
| --- | --- | --- |
|  | 14d | 1.9 |
|  | 28d | 1.75 |
| 60kPa | 7d | 2.02 |
|  | 14d | 1.9 |
|  | 28d | 2.37 |
| 90kPa | 7d | 2.43 |
|  | 14d | 2.14 |
|  | 28d | 2.5 |

(c) (σ_1_-σ_3_)_f_ -age

| 7d | 30kPa | 2.71 |
| --- | --- | --- |
|  | 60kPa | 2.63 |
|  | 90kPa | 3.33 |
| 14d | 30kPa | 2.26 |
|  | 60kPa | 2.57 |
|  | 90kPa | 3.18 |
| 28d | 30kPa | 2.63 |
|  | 60kPa | 2.93 |
|  | 90kPa | 3.21 |

(d) (σ_1_-σ_3_)_f_ - confining pressure

| 30kPa | 7d | 2.71 |
| --- | --- | --- |
|  | 14d | 2.26 |
|  | 28d | 2.63 |
| 60kPa | 7d | 2.93 |
|  | 14d | 2.57 |
|  | 28d | 2.93 |
| 90kPa | 7d | 3.33 |
|  | 14d | 3.18 |
|  | 28d | 3.21 |
