# Supplementary material for: Material composition and constitutive model development of red mud-based filler for highway tunnel invert filling applications: A comprehensive study
Source: PLoS One. 2025 Apr 16;20(4):e0321926. doi: 10.1371/journal.pone.0321926 (PMC12002488; doi:10.1371/journal.pone.0321926)
Supplement: S8 Table — Test curve and fitting curve results. (DOCX) [file pone.0321926.s008.docx]

Table S8. The (σ1-σ3)-ε1 curves of URM with confining pressure under different ages (Fig.13). Test curve and fitting curve results.

(a) 7d

| 30kPa | | Fitting data | | 60kPa | | Fitting data | | 90kPa | | Fitting data | |
| --- | --- | --- | --- | --- | --- | --- | --- | --- | --- | --- | --- |
| ε_1_ | (σ_1_-σ_3_) | ε_1_ | (σ_1_-σ_3_) | ε_1_ | (σ_1_-σ_3_) | ε_1_ | (σ_1_-σ_3_) | ε_1_ | (σ_1_-σ_3_) | ε_1_ | (σ_1_-σ_3_) |
| 0 | 0.2223 | 3.7851 | 55.58546 | 0.0017 | -2.9 | 3.1933 | 64.58909 | 0 | 1.3112 | 0.8632 | 29.68698 |
| 0.0019 | 0.4645 | 3.9901 | 81.18062 | 0 | -1.6 | 3.4001 | 93.46422 | 0.0058 | 1.0567 | 1.0815 | 68.01646 |
| 0.0019 | 0.2871 | 4.1987 | 104.91338 | 0 | -2.6 | 3.6335 | 122.96726 | 0.0095 | 1.0475 | 1.2731 | 98.8795 |
| 0.0019 | 0.5099 | 4.3999 | 125.74362 | 0 | 1.1 | 3.8212 | 144.48375 | 0.0095 | 0.8638 | 1.4951 | 131.59532 |
| 0.0019 | 0.722 | 4.6105 | 145.53056 | -0.0019 | 1.8 | 4.0376 | 167.02161 | 0.0132 | 0.2591 | 1.6868 | 157.37886 |
| 0.0076 | 0.529 | 4.804 | 162.01954 | -0.0019 | 4.4 | 4.2463 | 186.61976 | 0.0114 | 1.3179 | 1.8955 | 183.01811 |
| 0.0095 | 1.0034 | 5.0165 | 178.39745 | -0.0019 | 5.4 | 4.4587 | 204.57393 | 0.0132 | 0.5444 | 2.1023 | 206.08647 |
| 0.0132 | 0.9784 | 5.2404 | 193.8442 | -0.0019 | 1.7 | 4.6524 | 219.32811 | 0.0171 | 1.832 | 2.328 | 228.79365 |
| 0.0209 | 1.2021 | 5.4663 | 207.70228 | -0.0019 | 0.8 | 4.8781 | 234.72635 | 0.0171 | 1.0465 | 2.5405 | 247.9875 |
| 0.0265 | 1.8452 | 5.6862 | 219.66992 | 0.0039 | 1.8 | 5.0565 | 245.638 | 0.0209 | 1.8114 | 2.7492 | 264.92897 |
| 0.0285 | 1.4944 | 5.8627 | 228.27917 | 0.0058 | 0.7 | 5.2614 | 256.91022 | 0.0228 | 1.3142 | 2.9655 | 280.64177 |
| 0.0304 | 2.04 | 6.0809 | 237.79952 | 0.0058 | 2 | 5.4795 | 267.55108 | 0.0228 | 1.8284 | 3.1439 | 292.29195 |
| 0.0304 | 1.4499 | 6.2859 | 245.70905 | 0.0115 | 1.6 | 5.694 | 276.77475 | 0.0247 | 1.7778 | 3.3659 | 305.26444 |
| 0.0304 | 1.4987 | 6.4965 | 252.88757 | 0.0229 | 12.5 | 5.8931 | 284.33706 | 0.0285 | 2.6579 | 3.5728 | 315.9518 |
| 0.0323 | 1.7994 | 6.709 | 259.25086 | 0.0343 | 13.6 | 6.1019 | 291.33277 | 0.0323 | 2.4017 | 3.7852 | 325.63578 |
| 0.0323 | 1.7783 | 6.9195 | 264.76985 | 0.0438 | 3.7 | 6.3257 | 297.87458 | 0.0361 | 3.6283 | 3.9977 | 334.13271 |
| 0.0341 | 2.5738 | 7.1206 | 269.38796 | 0.0551 | 4.4 | 6.5401 | 303.31247 | 0.0361 | 1.8266 | 4.1894 | 340.8654 |
| 0.0341 | 2.3067 | 7.3674 | 274.27975 | 0.0589 | 4.6 | 6.7489 | 307.91529 | 0.0417 | 2.8958 | 4.398 | 347.27777 |
| 0.0341 | 2.0983 | 7.5362 | 277.18762 | 0.0626 | 5.4 | 6.9633 | 312.01161 | 0.0436 | 3.3848 | 4.6371 | 353.57083 |
| 0.0379 | 2.57 | 7.7468 | 280.37546 | 0.076 | 4.6 | 7.1758 | 315.51737 | 0.0475 | 2.5924 | 4.8135 | 357.5621 |
| 0.0379 | 2.3014 | 7.9631 | 283.20063 | 0.0969 | 4.6 | 7.3845 | 318.49105 | 0.0513 | 3.3237 | 5.0318 | 361.81619 |
| 0.0417 | 2.5151 | 8.1529 | 285.35282 | 0.1252 | 4.4 | 7.5989 | 321.12595 | 0.0531 | 2.6494 | 5.231 | 365.10503 |
| 0.0455 | 2.8081 | 8.3767 | 287.55208 | 0.1575 | 4.2 | 7.8076 | 323.33921 | 0.0531 | 2.3889 | 5.453 | 368.18087 |
| 0.0455 | 2.7855 | 8.5816 | 289.29061 | 0.1633 | 2.6 | 7.9859 | 324.99438 | 0.057 | 2.8622 | 5.6465 | 370.41457 |
| 0.0455 | 3.0006 | 8.796 | 290.8732 | 0.1784 | 3.9 | 8.2023 | 326.75577 | 0.0589 | 2.8431 | 5.8627 | 372.48014 |
| 0.0494 | 3.047 | 9.0085 | 292.24339 | 0.1898 | 3.5 | 8.4299 | 328.36457 | 0.0607 | 2.8904 | 6.0771 | 374.13974 |
| 0.0494 | 3.0164 | 9.204 | 293.36111 | 0.1974 | 2.4 | 8.6367 | 329.65075 | 0.0607 | 2.6435 | 6.2745 | 375.37416 |
| 0.0513 | 2.9975 | 9.3975 | 294.35849 | 0.2164 | 2.5 | 8.8321 | 330.7435 | 0.0589 | 3.111 | 6.4965 | 376.47783 |
| 0.055 | 3.2998 | 9.6119 | 295.36403 | 0.2259 | 2.6 | 9.0446 | 331.82642 | 0.0607 | 3.3418 | 6.7128 | 377.31156 |
| 0.0587 | 4.0833 | 9.8186 | 296.25858 | 0.2354 | 2.7 | 9.1984 | 332.55733 | 0.0626 | 3.0714 | 6.9006 | 377.87662 |
| 0.0626 | 3.8775 | 10.0274 | 297.10929 | 0.2543 | 3.4 | 9.4506 | 333.68719 | 0.0645 | 3.1013 | 7.1113 | 378.36888 |
| 0.0645 | 4.0868 | 10.2456 | 297.96163 | 0.2656 | 2.5 | 9.6745 | 334.64369 | 0.0626 | 3.1191 | 7.3161 | 378.73424 |
| 0.0626 | 3.2563 | 10.441 | 298.70774 | 0.2733 | 0.7 | 9.8719 | 335.46827 | 0.0645 | 3.8196 | 7.5286 | 379.02503 |
| 0.0645 | 3.2477 | 10.6593 | 299.53594 | 0.2885 | -0.5 | 10.0881 | 336.3659 | 0.0645 | 3.5565 | 7.7487 | 379.25993 |
| 0.0645 | 3.8123 | 10.8792 | 300.37647 | 0.3055 | -0.6 | 10.2874 | 337.19874 | 0.0645 | 3.6241 | 7.9555 | 379.44238 |
| 0.0645 | 4.6257 | 11.0822 | 301.16643 | 0.3131 | 0.5 | 10.4922 | 338.06813 | 0.0645 | 3.057 | 8.17 | 379.61303 |
| 0.0645 | 4.3739 | 11.2986 | 302.02991 | 0.3169 | -0.2 | 10.6954 | 338.95015 | 0.0645 | 3.5495 | 8.3654 | 379.76696 |
| 0.0664 | 4.3695 | 11.4979 | 302.84857 | 0.3187 | 2.5873 | 10.9267 | 339.9825 | 0.0645 | 3.7987 | 8.5911 | 379.95823 |
| 0.2485 | 8.4947 | 11.6875 | 303.65015 | 0.4706 | 8.686 | 11.1051 | 340.80109 | 0.2524 | 11.0757 | 8.7923 | 380.15196 |
| 0.4591 | 10.2549 | 11.9209 | 304.66813 | 0.7173 | 9.5087 | 11.3443 | 341.92939 | 0.4364 | 16.1269 | 8.9991 | 380.3826 |
| 0.6679 | 13.2873 | 12.124 | 305.58113 | 0.9051 | 9.1073 | 11.551 | 342.93111 | 0.666 | 25.7951 | 9.223 | 380.67575 |
| 0.85 | 15.8624 | 12.3289 | 306.52566 | 1.1327 | 11.1981 | 11.7465 | 343.89854 | 0.98675 | 45.01002 | 9.4165 | 380.96972 |
| 1.1043 | 17.8062 | 12.5281 | 307.46338 | 1.313 | 15.8558 | 11.9609 | 344.97748 | 1.04012 | 73.04077 | 9.6005 | 381.28628 |
| 1.2769 | 17.0807 | 12.7595 | 308.57153 | 1.5292 | 18.5962 | 12.1676 | 346.03006 | 1.24471 | 92.6039 | 9.8282 | 381.72901 |
| 1.4838 | 11.3609 | 12.9474 | 309.48143 | 1.7494 | 19.645 | 12.3555 | 346.99211 | 1.39593 | 120.34267 | 10.0482 | 382.20965 |
| 1.6924 | 13.7066 | 13.1485 | 310.45977 | 1.9429 | 25.8746 | 12.5812 | 348.14659 | 1.56939 | 146.32951 | 10.2305 | 382.64561 |
| 1.903 | 15.6408 | 13.3572 | 311.47368 | 2.146 | 29.5913 | 12.7937 | 349.22406 | 1.79621 | 166.7686 | 10.4544 | 383.22428 |
| 2.0832 | 12.5403 | 13.5849 | 312.56994 | 2.3831 | 30.9294 | 13.0062 | 350.2834 | 2.06307 | 195.38333 | 10.6669 | 383.81284 |
| 2.3394 | 16.0364 | 13.7936 | 313.55754 | 2.5064 | 32.0954 | 13.2074 | 351.26157 | 2.28989 | 218.15831 | 10.866 | 384.39385 |
| 2.5537 | 15.5548 | 14.008 | 314.54675 | 2.7986 | 37.6391 | 13.4047 | 352.18976 | 2.48114 | 241.51728 | 11.0729 | 385.02194 |
| 2.7492 | 15.7249 | 14.211 | 315.45233 | 2.9751 | 38.4801 | 13.6096 | 353.11344 | 2.72131 | 258.74451 | 11.2721 | 385.64382 |
| 2.9731 | 15.9515 | 14.3989 | 316.25762 | 3.20946 | 41.83375 | 13.824 | 354.0277 | 2.98372 | 274.80379 | 11.5016 | 386.37258 |
| 3.142 | 10.6958 | 14.6114 | 317.12357 | 3.26497 | 56.79922 | 14.0309 | 354.8516 | 3.17496 | 299.62269 | 11.7218 | 387.07493 |
| 3.3905 | 19.901 | 14.8144 | 317.90028 | 3.28456 | 69.66017 | 14.2566 | 355.67676 | 3.4774 | 313.34608 | 11.8983 | 387.6336 |
| 3.584 | 21.3216 | 15.0174 | 318.677 | 3.35966 | 84.04105 | 14.4464 | 356.30561 | 3.76649 | 327.06947 | 12.1277 | 388.34534 |
| 3.81836 | 37.18555 |  |  | 3.43476 | 103.09864 | 14.655 | 356.9232 | 4.00666 | 343.12875 | 12.3119 | 388.89845 |
| 3.87387 | 50.48819 |  |  | 3.51313 | 122.74082 | 14.8732 | 357.48254 | 4.27796 | 357.7281 | 12.5433 | 389.56122 |
| 3.89346 | 61.92015 |  |  | 3.64375 | 135.71869 | 14.9937 | 357.7523 | 4.72272 | 357.7281 | 12.7501 | 390.1157 |
| 3.96856 | 74.70316 |  |  | 3.74171 | 152.08718 |  |  | 5.16748 | 362.3999 | 12.938 | 390.58273 |
| 4.04366 | 91.64324 |  |  | 3.9311 | 174.53538 |  |  | 5.58555 | 357.43612 | 13.1694 | 391.103 |
| 4.12203 | 109.10295 |  |  | 4.27396 | 194.87907 |  |  | 5.98583 | 366.19573 | 13.3744 | 391.50797 |
| 4.25265 | 120.63884 |  |  | 4.40457 | 205.75242 |  |  | 6.37722 | 378.75117 | 13.5869 | 391.86785 |
| 4.35061 | 135.1886 |  |  | 4.55804 | 218.73029 |  |  | 6.76861 | 381.08706 | 13.7917 | 392.15408 |
| 4.54 | 155.14256 |  |  | 4.78661 | 230.30514 |  |  | 7.21337 | 381.67104 | 14.0024 | 392.38482 |
| 4.88286 | 173.22584 |  |  | 5.01519 | 238.48938 |  |  | 7.65812 | 372.91143 | 14.2015 | 392.54332 |
| 5.01347 | 182.89104 |  |  | 5.2405 | 254.03944 |  |  | 8.10288 | 376.12329 | 14.4026 | 392.646 |
| 5.16694 | 194.42692 |  |  | 5.48866 | 262.22368 |  |  | 8.54764 | 379.91912 | 14.6133 | 392.69482 |
| 5.39551 | 204.71568 |  |  | 5.73683 | 273.79854 |  |  | 8.9924 | 382.83899 | 14.8296 | 392.68787 |
| 5.62409 | 211.99056 |  |  | 6.19398 | 283.97038 |  |  | 9.43716 | 379.33514 | 15.0403 | 392.73669 |
| 5.8494 | 225.81284 |  |  | 6.47806 | 292.85613 |  |  | 9.88192 | 377.58322 |  |  |
| 6.09756 | 233.08772 |  |  | 6.72623 | 298.93585 |  |  | 10.32667 | 388.38674 |  |  |
| 6.34573 | 243.37648 |  |  | 7.08541 | 307.1201 |  |  | 10.78454 | 386.00108 |  |  |
| 6.80288 | 252.41812 |  |  | 7.40868 | 311.21222 |  |  | 11.24169 | 386.00108 |  |  |
| 7.08696 | 260.31656 |  |  | 7.73195 | 320.09797 |  |  | 11.60087 | 388.57327 |  |  |
| 7.33513 | 265.72076 |  |  | 7.96052 | 324.89159 |  |  | 12.09394 | 389.64502 |  |  |
| 7.69431 | 272.99564 |  |  | 8.3589 | 329.5683 |  |  | 12.60986 | 389.64502 |  |  |
| 8.01758 | 276.63308 |  |  | 8.75727 | 332.37433 |  |  | 13.02783 | 389.64502 |  |  |
| 8.34085 | 284.53152 |  |  | 9.10013 | 335.76494 |  |  | 13.4262 | 389.64502 |  |  |
| 8.56942 | 288.79253 |  |  | 9.4038 | 338.45405 |  |  | 13.72987 | 390.93111 |  |  |
| 8.9678 | 292.9496 |  |  | 9.69115 | 341.84466 |  |  | 13.99763 | 390.93111 |  |  |
| 9.36617 | 295.44385 |  |  | 10.30969 | 344.99339 |  |  | 14.3209 | 393.5033 |  |  |
| 9.70903 | 298.45773 |  |  | 10.75445 | 345.63045 |  |  | 14.81397 | 393.5033 |  |  |
| 10.0127 | 300.84804 |  |  | 11.19921 | 346.42678 |  |  |  |  |  |  |
| 10.30005 | 303.86192 |  |  | 11.64396 | 345.15265 |  |  |  |  |  |  |
| 10.91859 | 306.66079 |  |  | 12.08872 | 343.87853 |  |  |  |  |  |  |
| 11.36335 | 307.22707 |  |  | 12.53348 | 343.87853 |  |  |  |  |  |  |
| 11.80811 | 307.93491 |  |  | 12.97824 | 344.03779 |  |  |  |  |  |  |
| 12.25286 | 306.80236 |  |  | 13.423 | 347.22311 |  |  |  |  |  |  |
| 12.69762 | 305.6698 |  |  | 13.86775 | 349.77136 |  |  |  |  |  |  |
| 13.14238 | 305.6698 |  |  | 14.31251 | 350.88622 |  |  |  |  |  |  |
| 13.58714 | 305.81137 |  |  | 14.75727 | 350.88622 |  |  |  |  |  |  |
| 14.0319 | 308.64276 |  |  | 15.20203 | 353.753 |  |  |  |  |  |  |
| 14.47665 | 310.90787 |  |  |  |  |  |  |  |  |  |  |
| 14.92141 | 311.89886 |  |  |  |  |  |  |  |  |  |  |

(b) 14d

| 30kPa | | Fitting data | | 60kPa | | Fitting data | | 90kPa | | Fitting data | |
| --- | --- | --- | --- | --- | --- | --- | --- | --- | --- | --- | --- |
| ε_1_ | (σ_1_-σ_3_) | ε_1_ | (σ_1_-σ_3_) | ε_1_ | (σ_1_-σ_3_) | ε_1_ | (σ_1_-σ_3_) | ε_1_ | (σ_1_-σ_3_) | ε_1_ | (σ_1_-σ_3_) |
| 0 | 1.0304 | 3.8365 | 60.07034 | 0.002 | 1.6009 | 3.8212 | 38.50159 | -0.0019 | 3.1325 | 1.9789 | 60.49315 |
| 0.002 | 1.023 | 4.0167 | 83.38118 | 0 | 1.6061 | 4.0376 | 73.38905 | 0 | 3.3791 | 2.1705 | 92.61963 |
| 0.0039 | 1.0153 | 4.2197 | 107.14532 | 0 | 1.8524 | 4.2463 | 103.59594 | 0 | 2.954 | 2.3679 | 122.71162 |
| 0.0039 | 1.0011 | 4.436 | 129.77175 | 0 | 1.5948 | 4.4587 | 131.13771 | -0.0019 | 2.6931 | 2.5785 | 151.69183 |
| 0.0039 | 0.9903 | 4.6295 | 147.84059 | -0.0019 | 1.5878 | 4.6524 | 153.65479 | -0.0019 | 2.9262 | 2.8175 | 180.98194 |
| 0.0039 | 0.9777 | 4.8325 | 164.76808 | -0.0019 | 1.5758 | 4.8781 | 177.0156 | -0.0039 | 2.6569 | 2.9864 | 199.5539 |
| 0.0076 | 0.9594 | 5.0564 | 181.23081 | -0.0019 | 1.8095 | 5.0565 | 193.46293 | -0.0039 | 2.6387 | 3.2216 | 222.72551 |
| 0.0076 | 1.8535 | 5.2556 | 194.10038 | -0.0019 | 1.5408 | 5.2614 | 210.33709 | -0.0058 | 2.868 | 3.419 | 239.94967 |
| 0.0114 | 2.0789 | 5.4739 | 206.45939 | -0.0019 | 1.6073 | 5.4795 | 226.12939 | -0.0076 | 2.9278 | 3.6068 | 254.61324 |
| 0.0134 | 1.8114 | 5.6825 | 216.72365 | 0.0039 | 1.8326 | 5.694 | 239.68053 | -0.0076 | 2.9045 | 3.8535 | 271.55086 |
| 0.0134 | 1.2086 | 5.8627 | 224.48873 | 0.0058 | 1.565 | 5.8931 | 250.66775 | -0.0058 | 3.1231 | 4.0602 | 283.8999 |
| 0.0152 | 2.1021 | 6.0848 | 232.79191 | 0.0058 | 1.5424 | 6.1019 | 260.70417 | -0.0076 | 3.1773 | 4.2785 | 295.30329 |
| 0.0152 | 2.0779 | 6.2916 | 239.39267 | 0.0115 | 1.5972 | 6.3257 | 269.94478 | -0.0076 | 3.1505 | 4.4834 | 304.6267 |
| 0.019 | 1.8005 | 6.5136 | 245.40219 | 0.0229 | 1.814 | 6.5401 | 277.48564 | -0.0114 | 3.1154 | 4.6788 | 312.39866 |
| 0.0247 | 2.5236 | 6.7222 | 250.15084 | 0.0343 | 1.6112 | 6.7489 | 283.73642 | -0.0114 | 3.4277 | 4.8856 | 319.55637 |
| 0.0266 | 2.0728 | 6.9386 | 254.2707 | 0.0438 | 1.8302 | 6.9633 | 289.16453 | -0.0152 | 3.3857 | 5.0944 | 325.78913 |
| 0.0266 | 2.3024 | 7.134 | 257.37318 | 0.0551 | 1.7909 | 7.1758 | 293.67652 | -0.0152 | 3.6856 | 5.3125 | 331.3565 |
| 0.0285 | 2.5288 | 7.3256 | 259.91908 | 0.0589 | 1.8425 | 7.3845 | 297.3761 | -0.038 | 3.4154 | 5.5308 | 336.08304 |
| 0.0323 | 2.8222 | 7.5534 | 262.39452 | 0.0626 | 1.8064 | 7.5989 | 300.52559 | -0.0417 | 3.3846 | 5.7394 | 339.91105 |
| 0.0323 | 2.302 | 7.7639 | 264.22915 | 0.076 | 1.8499 | 7.8076 | 303.04984 | -0.0513 | 3.1869 | 5.9462 | 343.1306 |
| 0.0343 | 2.3416 | 7.9897 | 265.79522 | 0.0969 | 1.7966 | 7.9859 | 304.84624 | -0.0531 | 3.394 | 6.1454 | 345.76416 |
| 0.0343 | 2.5696 | 8.1719 | 266.80751 | 0.1252 | 1.8207 | 8.2023 | 306.65208 | -0.055 | 3.3537 | 6.3428 | 347.98543 |
| 0.038 | 3.0332 | 8.3767 | 267.72682 | 0.1575 | 2.6717 | 8.4299 | 308.18738 | -0.0607 | 3.4001 | 6.559 | 350.04201 |
| 0.0417 | 3.4093 | 8.595 | 268.50301 | 0.1633 | 2.6217 | 8.6367 | 309.32497 | -0.0626 | 3.4299 | 6.7582 | 351.64511 |
| 0.0417 | 3.0612 | 8.8094 | 269.10871 | 0.1784 | 2.6517 | 8.8321 | 310.22473 | -0.0664 | 3.6393 | 6.9651 | 353.06595 |
| 0.0475 | 3.3527 | 9.001 | 269.55337 | 0.1898 | 2.8443 | 9.0446 | 311.05789 | -0.0684 | 3.6769 | 7.1681 | 354.26299 |
| 0.0513 | 3.3311 | 9.2249 | 269.99381 | 0.1974 | 2.8806 | 9.1984 | 311.59166 | -0.0703 | 3.6271 | 7.3616 | 355.2585 |
| 0.0531 | 2.802 | 9.4165 | 270.32878 | 0.2164 | 2.8929 | 9.4506 | 312.38527 | -0.0759 | 3.668 | 7.595 | 356.31298 |
| 0.0531 | 3.5882 | 9.6499 | 270.71364 | 0.2259 | 2.8501 | 9.6745 | 313.04503 | -0.0759 | 3.6051 | 7.8207 | 357.21986 |
| 0.0531 | 3.3892 | 9.8586 | 271.05791 | 0.2354 | 2.8726 | 9.8719 | 313.62024 | -0.0759 | 3.8863 | 8.0067 | 357.9081 |
| 0.057 | 3.3646 | 10.0369 | 271.36499 | 0.2543 | 3.1332 | 10.0881 | 314.267 | -0.0759 | 3.9106 | 8.2344 | 358.70222 |
| 0.0645 | 3.8197 | 10.2532 | 271.76553 | 0.2656 | 3.0856 | 10.2874 | 314.8958 | -0.0759 | 3.8803 | 8.4487 | 359.42012 |
| 0.0645 | 3.889 | 10.4734 | 272.21486 | 0.2733 | 3.3829 | 10.4922 | 315.588 | -0.0759 | 3.8478 | 8.6518 | 360.08785 |
| 0.0721 | 4.116 | 10.6915 | 272.70825 | 0.2885 | 3.3522 | 10.6954 | 316.3307 | -0.0759 | 4.2417 | 8.8377 | 360.69627 |
| 0.076 | 4.1 | 10.8775 | 273.16975 | 0.3055 | 3.6432 | 10.9267 | 317.25221 | -0.0759 | 3.8951 | 9.0408 | 361.36382 |
| 0.0779 | 3.3481 | 11.1004 | 273.77554 | 0.3131 | 3.6061 | 11.1051 | 318.02159 | -0.0759 | 4.4516 | 9.259 | 362.08825 |
| 0.0797 | 4.0725 | 11.3005 | 274.35856 | 0.3169 | 3.8462 | 11.3443 | 319.1345 | -0.0759 | 4.1852 | 9.4544 | 362.7445 |
| 0.0816 | 4.0604 | 11.5111 | 275.01672 | 0.3187 | 3.5708 | 11.551 | 320.16954 | -0.0759 | 4.1746 | 9.6764 | 363.49737 |
| 0.2619 | 6.3666 | 11.7122 | 275.67959 | 0.4706 | 5.3761 | 11.7465 | 321.20751 | 0.1139 | 11.7253 | 9.904 | 364.27316 |
| 0.4857 | 8.9729 | 11.8944 | 276.30363 | 0.7173 | 6.6807 | 11.9609 | 322.40596 | 0.3149 | 17.9279 | 10.109 | 364.96972 |
| 0.666 | 8.6852 | 12.124 | 277.11261 | 0.9051 | 7.3892 | 12.1676 | 323.61355 | 0.5141 | 25.1195 | 10.3157 | 365.6632 |
| 0.8899 | 9.6688 | 12.3497 | 277.92076 | 1.1327 | 8.2228 | 12.3555 | 324.74845 | 0.7285 | 32.9787 | 10.5245 | 366.34681 |
| 1.1024 | 8.8705 | 12.5548 | 278.65503 | 1.313 | 9.2877 | 12.5812 | 326.14792 | 0.9467 | 35.9758 | 10.7331 | 367.00399 |
| 1.2902 | 9.5788 | 12.7653 | 279.39715 | 1.5292 | 9.9652 | 12.7937 | 327.49032 | 1.1574 | 41.0068 | 10.9476 | 367.64304 |
| 1.499 | 10.1284 | 12.9589 | 280.05956 | 1.7494 | 11.7296 | 13.0062 | 328.84476 | 1.3395 | 43.8018 | 11.1468 | 368.19412 |
| 1.7057 | 10.855 | 13.1809 | 280.78344 | 1.9429 | 13.7064 | 13.2074 | 330.12747 | 1.5615 | 58.9569 | 11.3346 | 368.66861 |
| 1.9373 | 12.6266 | 13.3744 | 281.37341 | 2.146 | 16.2531 | 13.4047 | 331.37578 | 1.7759 | 67.5242 | 11.5661 | 369.18325 |
| 2.127 | 12.8078 | 13.6039 | 282.01165 | 2.3831 | 19.9732 | 13.6096 | 332.65238 | 2.20688 | 79.45534 | 11.7673 | 369.55905 |
| 2.3319 | 13.8547 | 13.7994 | 282.49361 | 2.5064 | 20.9853 | 13.824 | 333.95642 | 2.32226 | 107.12551 | 11.9684 | 369.86116 |
| 2.5576 | 14.7602 | 14.0194 | 282.95884 | 2.7986 | 25.1302 | 14.0309 | 335.17504 | 2.40658 | 134.79568 | 12.1751 | 370.0892 |
| 2.7759 | 17.7381 | 14.2091 | 283.28818 | 2.9751 | 25.911 | 14.2566 | 336.45098 | 2.55855 | 155.74233 | 12.3801 | 370.22832 |
| 2.9789 | 18.4505 | 14.4292 | 283.58111 | 3.1933 | 29.7835 | 14.4464 | 337.47544 | 2.72797 | 177.07079 | 12.6172 | 370.2777 |
| 3.1933 | 23.1805 | 14.6436 | 283.7704 | 3.4001 | 31.98 | 14.655 | 338.54637 | 2.91694 | 199.41489 | 12.8108 | 370.22879 |
| 3.3981 | 28.1347 | 14.8296 | 283.85642 | 3.6335 | 40.3847 | 14.8732 | 338.54637 | 3.10591 | 221.96212 | 13.0271 | 370.08146 |
| 3.5803 | 35.9199 |  |  | 3.97957 | 56.41266 |  |  | 3.33398 | 239.63427 | 13.2358 | 369.85162 |
| 3.86484 | 43.76082 |  |  | 4.14899 | 78.95988 |  |  | 3.60114 | 258.52519 | 13.4218 | 369.5808 |
| 3.9669 | 71.43099 |  |  | 4.24347 | 102.52275 |  |  | 3.95953 | 275.1817 | 13.6531 | 369.17009 |
| 4.10003 | 99.10116 |  |  | 4.35751 | 119.17926 |  |  | 4.24625 | 289.40067 | 13.8637 | 368.74125 |
| 4.22937 | 107.1947 |  |  | 4.47154 | 135.63264 |  |  | 4.51015 | 301.1821 | 14.0667 | 368.29764 |
| 4.3434 | 128.52316 |  |  | 4.64096 | 156.9611 |  |  | 4.8718 | 312.96354 | 14.2736 | 367.838 |
| 4.49328 | 145.17967 |  |  | 4.79409 | 175.85202 |  |  | 5.28884 | 323.5262 | 14.4804 | 367.39863 |
| 4.68225 | 166.305 |  |  | 4.98306 | 192.3054 |  |  | 5.68632 | 331.85446 | 14.691 | 367.00552 |
| 4.89402 | 181.74274 |  |  | 5.21113 | 210.18068 |  |  | 5.95349 | 338.96395 | 14.9016 | 366.70694 |
| 5.21332 | 197.18048 |  |  | 5.41965 | 221.96212 |  |  | 6.37052 | 346.07343 |  |  |
| 5.46093 | 208.96191 |  |  | 5.62816 | 236.18109 |  |  | 6.67353 | 353.18292 |  |  |
| 5.70855 | 217.29017 |  |  | 5.89533 | 244.30622 |  |  | 7.09056 | 356.6361 |  |  |
| 6.06694 | 225.4153 |  |  | 6.19833 | 256.29078 |  |  | 7.45221 | 359.07363 |  |  |
| 6.44813 | 234.96232 |  |  | 6.65121 | 273.96293 |  |  | 7.88554 | 360.2924 |  |  |
| 6.71204 | 242.07181 |  |  | 7.01285 | 283.30683 |  |  | 8.30258 | 362.52681 |  |  |
| 7.11279 | 249.18129 |  |  | 7.28002 | 291.63508 |  |  | 8.68377 | 364.96435 |  |  |
| 7.43534 | 259.74396 |  |  | 7.77199 | 296.30703 |  |  | 9.06171 | 364.96435 |  |  |
| 7.62431 | 265.63467 |  |  | 8.20858 | 301.1821 |  |  | 9.42336 | 364.96435 |  |  |
| 7.88821 | 270.30662 |  |  | 8.51158 | 305.85405 |  |  | 9.8404 | 364.96435 |  |  |
| 8.30525 | 273.96293 |  |  | 8.92862 | 307.07282 |  |  | 10.37147 | 364.96435 |  |  |
| 8.72554 | 273.96293 |  |  | 9.34565 | 305.85405 |  |  | 10.71357 | 364.96435 |  |  |
| 9.25661 | 277.41611 |  |  | 9.76269 | 307.07282 |  |  | 11.1697 | 366.18312 |  |  |
| 9.80397 | 278.63488 |  |  | 10.12434 | 310.526 |  |  | 11.58674 | 368.41753 |  |  |
| 10.12652 | 278.63488 |  |  | 10.44689 | 310.526 |  |  | 11.98422 | 368.41753 |  |  |
| 10.41324 | 282.29118 |  |  | 10.71079 | 311.74477 |  |  | 12.34587 | 367.40189 |  |  |
| 10.86611 | 281.07242 |  |  | 11.07244 | 314.18231 |  |  | 12.72381 | 367.40189 |  |  |
| 11.26686 | 281.07242 |  |  | 11.45038 | 314.18231 |  |  | 13.1604 | 367.40189 |  |  |
| 11.62525 | 284.52559 |  |  | 11.81203 | 315.40108 |  |  | 13.53834 | 367.40189 |  |  |
| 12.00645 | 284.52559 |  |  | 12.13458 | 316.41672 |  |  | 13.95537 | 367.40189 |  |  |
| 12.44303 | 284.52559 |  |  | 12.43758 | 316.41672 |  |  | 14.33657 | 367.40189 |  |  |
| 12.74603 | 288.1819 |  |  | 12.76014 | 316.41672 |  |  | 14.65912 | 367.40189 |  |  |
| 13.08813 | 286.96313 |  |  | 13.21627 | 316.41672 |  |  | 14.98167 | 369.6363 |  |  |
| 13.44652 | 286.96313 |  |  | 13.49972 | 317.63549 |  |  |  |  |  |  |
| 13.95804 | 289.40067 |  |  | 13.78318 | 318.85426 |  |  |  |  |  |  |
| 14.2806 | 291.63508 |  |  | 14.06989 | 318.85426 |  |  |  |  |  |  |
| 14.77583 | 294.07262 |  |  | 14.42828 | 317.63549 |  |  |  |  |  |  |
|  |  |  |  | 14.80948 | 321.29179 |  |  |  |  |  |  |

(c) 28d

| 30kPa | | Fitting data | | 60kPa | | Fitting data | | 90kPa | | Fitting data | |
| --- | --- | --- | --- | --- | --- | --- | --- | --- | --- | --- | --- |
| ε_1_ | (σ_1_-σ_3_) | ε_1_ | (σ_1_-σ_3_) | ε_1_ | (σ_1_-σ_3_) | ε_1_ | (σ_1_-σ_3_) | ε_1_ | (σ_1_-σ_3_) | ε_1_ | (σ_1_-σ_3_) |
| 0 | 2.3863 | 3.1989 | 42.83788 | -0.0019 | 1.3017 | 3.1116 | 52.51174 | 0 | 1.3703 | 1.2199 | 32.69049 |
| 0.0038 | 2.125 | 3.4 | 67.76928 | 0 | 1.2959 | 3.3241 | 80.24229 | 0 | 1.6147 | 1.421 | 63.96365 |
| 0.0038 | 1.6045 | 3.5993 | 90.52098 | 0 | 1.1177 | 3.5271 | 104.44793 | 0.0019 | 1.3576 | 1.6544 | 97.18954 |
| 0.0038 | 1.5826 | 3.8364 | 115.21365 | -0.0019 | 1.3579 | 3.7282 | 126.37382 | 0.0038 | 1.5937 | 1.8214 | 119.06257 |
| 0.0056 | 1.5547 | 4.0431 | 134.77478 | -0.0019 | 1.099 | 3.9389 | 147.30502 | 0.0095 | 1.5869 | 2.0452 | 146.05041 |
| 0.0095 | 2.4423 | 4.2481 | 152.49035 | -0.0019 | 1.086 | 4.1723 | 168.23261 | 0.0114 | 1.3285 | 2.2426 | 167.7829 |
| 0.0095 | 1.5839 | 4.415 | 165.75208 | -0.0019 | 1.0699 | 4.379 | 184.93538 | 0.0228 | 1.312 | 2.4665 | 190.24668 |
| 0.0095 | 2.1375 | 4.6731 | 184.35084 | -0.0019 | 1.048 | 4.582 | 199.79522 | 0.0265 | 1.5495 | 2.6733 | 209.07455 |
| 0.0132 | 2.4274 | 4.8534 | 196.06161 | 0.0019 | 1.3569 | 4.7546 | 211.31335 | 0.036 | 1.2915 | 2.8896 | 226.93943 |
| 0.0114 | 2.145 | 5.081 | 209.45544 | 0.0075 | 1.3266 | 4.9994 | 226.02864 | 0.0455 | 1.6048 | 3.1003 | 242.67765 |
| 0.017 | 2.6174 | 5.2745 | 219.71089 | 0.0114 | 1.2872 | 5.1968 | 236.62102 | 0.0664 | 1.5926 | 3.3033 | 256.40447 |
| 0.019 | 2.4142 | 5.4851 | 229.78208 | 0.0228 | 1.327 | 5.4111 | 246.94629 | 0.0759 | 1.5728 | 3.5232 | 269.80737 |
| 0.019 | 2.1278 | 5.709 | 239.34115 | 0.0323 | 1.3668 | 5.6161 | 255.77737 | 0.0796 | 1.5641 | 3.7093 | 280.05015 |
| 0.019 | 2.1691 | 5.912 | 247.0696 | 0.0398 | 1.3267 | 5.8324 | 264.08657 | 0.0854 | 1.548 | 3.9672 | 292.72068 |
| 0.0228 | 2.6289 | 6.0961 | 253.36989 | 0.0436 | 1.3606 | 6.0411 | 271.21293 | 0.0967 | 1.6158 | 4.1438 | 300.46026 |
| 0.0265 | 2.9151 | 6.3256 | 260.36003 | 0.0569 | 1.3147 | 6.246 | 277.4397 | 0.1195 | 1.8483 | 4.3581 | 308.92049 |
| 0.0265 | 2.6267 | 6.5571 | 266.52855 | 0.0721 | 1.3416 | 6.4509 | 282.9777 | 0.1404 | 1.8265 | 4.5555 | 315.88511 |
| 0.0265 | 2.6753 | 6.762 | 271.32095 | 0.091 | 1.2894 | 6.6596 | 287.98135 | 0.1574 | 1.8064 | 4.7641 | 322.45955 |
| 0.0284 | 2.8709 | 6.9594 | 275.40238 | 0.11 | 1.302 | 6.8759 | 292.56035 | 0.1745 | 1.7925 | 4.9729 | 328.30677 |
| 0.0304 | 2.6604 | 7.1681 | 279.20096 | 0.129 | 1.3158 | 7.0751 | 296.29025 | 0.203 | 1.8469 | 5.1796 | 333.44221 |
| 0.0341 | 3.1156 | 7.3598 | 282.2681 | 0.165 | 1.5598 | 7.2932 | 299.89953 | 0.222 | 2.0713 | 5.3941 | 338.15379 |
| 0.0398 | 2.9045 | 7.5855 | 285.41618 | 0.184 | 1.5714 | 7.5001 | 302.91856 | 0.2448 | 2.046 | 5.6028 | 342.19714 |
| 0.0474 | 3.1655 | 7.7943 | 287.93179 | 0.2106 | 1.809 | 7.6955 | 305.45155 | 0.2561 | 2.6097 | 5.766 | 345.02484 |
| 0.055 | 3.3691 | 7.9782 | 289.86755 | 0.239 | 1.7969 | 7.8871 | 307.67127 | 0.2731 | 2.6685 | 6.0164 | 348.85616 |
| 0.0569 | 3.1365 | 8.1984 | 291.88119 | 0.2599 | 1.7966 | 8.1091 | 309.95811 | 0.2845 | 2.8931 | 6.2326 | 351.72723 |
| 0.0569 | 3.0936 | 8.3995 | 293.46572 | 0.2846 | 2.0435 | 8.3274 | 311.94963 | 0.3016 | 2.8756 | 6.4395 | 354.14433 |
| 0.0587 | 2.8837 | 8.6234 | 294.98291 | 0.2979 | 2.6142 | 8.5361 | 313.65033 | 0.3035 | 2.8581 | 6.6293 | 356.11219 |
| 0.0626 | 3.4014 | 8.8396 | 296.23604 | 0.3187 | 2.6423 | 8.741 | 315.15587 | 0.3244 | 3.17 | 6.8474 | 358.11628 |
| 0.0683 | 3.3342 | 9.0426 | 297.25177 | 0.3357 | 2.6401 | 8.9496 | 316.54729 | 0.3396 | 3.3919 | 7.0447 | 359.72324 |
| 0.0721 | 3.3689 | 9.2476 | 298.14404 | 0.3471 | 2.6448 | 9.1584 | 317.82016 | 0.3415 | 3.3721 | 7.2763 | 361.39602 |
| 0.0777 | 3.397 | 9.4411 | 298.88295 | 0.3661 | 2.8842 | 9.369 | 319.00286 | 0.3452 | 3.1037 | 7.4679 | 362.63089 |
| 0.0854 | 3.6759 | 9.6669 | 299.64083 | 0.3851 | 3.0908 | 9.5796 | 320.10155 | 0.3585 | 3.4203 | 7.6727 | 363.82445 |
| 0.0949 | 3.7117 | 9.8851 | 300.2872 | 0.3984 | 3.1234 | 9.7599 | 320.9867 | 0.3661 | 3.658 | 7.8625 | 364.83138 |
| 0.0986 | 3.6863 | 10.0919 | 300.83866 | 0.406 | 3.3388 | 9.9989 | 322.09505 | 0.3756 | 3.6495 | 8.0655 | 365.81925 |
| 0.0986 | 3.6674 | 10.2779 | 301.29524 | 0.4136 | 3.3959 | 10.2076 | 323.01265 | 0.3851 | 3.1434 | 8.3008 | 366.86853 |
| 0.1024 | 3.9685 | 10.4827 | 301.76531 | 0.4211 | 3.3722 | 10.3879 | 323.77374 | 0.3965 | 3.3663 | 8.4981 | 367.68295 |
| 0.1081 | 3.6985 | 10.6971 | 302.23083 | 0.4306 | 3.8336 | 10.6099 | 324.67604 | 0.4098 | 3.3668 | 8.7239 | 368.55514 |
| 0.1081 | 3.6873 | 10.9134 | 302.68204 | 0.4345 | 3.8209 | 10.8375 | 325.56548 | 0.4174 | 3.347 | 8.9231 | 369.28058 |
| 0.294 | 5.986 | 11.1221 | 303.10679 | 0.6204 | 5.9946 | 11.0178 | 326.24617 | 0.5939 | 4.2182 | 9.1413 | 370.0358 |
| 0.499 | 8.2087 | 11.3365 | 303.53773 | 0.8064 | 7.168 | 11.2265 | 327.00813 | 0.8139 | 5.1404 | 9.3537 | 370.73718 |
| 0.6981 | 10.0073 | 11.5339 | 303.93305 | 1.0378 | 10.0282 | 11.458 | 327.81976 | 1.0189 | 6.4884 | 9.5398 | 371.32759 |
| 0.8936 | 10.9981 | 11.754 | 304.3747 | 1.237 | 11.7684 | 11.6629 | 328.50655 | 1.2157 | 25.02977 | 9.7485 | 371.96506 |
| 1.1308 | 14.5558 | 11.9436 | 304.7569 | 1.459 | 14.8222 | 11.8621 | 329.14297 | 1.3116 | 41.35905 | 9.9515 | 372.56109 |
| 1.3186 | 17.0962 | 12.1619 | 305.19908 | 1.6659 | 17.904 | 12.0784 | 329.79519 | 1.38766 | 55.10384 | 10.1791 | 373.20101 |
| 1.5444 | 19.345 | 12.3763 | 305.63464 | 1.8708 | 21.116 | 12.2927 | 330.39672 | 1.50341 | 70.84573 | 10.3898 | 373.76537 |
| 1.755 | 24.3746 | 12.5793 | 306.04656 | 2.0795 | 26.2019 | 12.4958 | 330.92092 | 1.57947 | 81.77107 | 10.5776 | 374.24382 |
| 1.9637 | 29.0913 | 12.7785 | 306.44809 | 2.29 | 32.7498 | 12.7045 | 331.40783 | 1.69521 | 97.51296 | 10.7901 | 374.75454 |
| 2.1515 | 30.3294 | 12.9986 | 306.88548 | 2.4987 | 36.1525 | 12.8999 | 331.81144 | 1.73489 | 111.84513 | 11.0064 | 375.23731 |
| 2.383 | 29.1954 | 13.2072 | 307.29029 | 2.6847 | 40.5371 | 13.1219 | 332.20296 | 1.81095 | 125.47244 | 11.2207 | 375.67445 |
| 2.5804 | 35.4391 | 13.4028 | 307.65774 | 2.8973 | 55.0691 | 13.3211 | 332.48869 | 1.90686 | 142.62406 | 11.4295 | 376.05662 |
| 2.7795 | 37.0801 | 13.6229 | 308.05281 | 3.22638 | 61.87113 | 13.528 | 332.71516 | 2.08212 | 157.66109 | 11.6288 | 376.37708 |
| 3.0054 | 47.2347 | 13.8391 | 308.41717 | 3.36197 | 81.9693 | 13.7215 | 332.85851 | 2.23424 | 174.69522 | 11.8299 | 376.65267 |
| 3.39401 | 62.29092 | 14.0213 | 308.70242 | 3.47771 | 98.40375 | 13.9624 | 332.9402 | 2.3103 | 184.91571 | 12.0499 | 376.89483 |
| 3.54613 | 82.05163 | 14.2509 | 309.02884 | 3.66951 | 116.09433 | 14.1655 | 332.92289 | 2.50541 | 197.95563 | 12.2396 | 377.05054 |
| 3.68171 | 106.26818 |  |  | 3.84478 | 136.82057 | 14.3741 | 332.82145 | 2.67738 | 211.58294 | 12.4598 | 377.1661 |
| 3.95288 | 122.63857 |  |  | 3.97706 | 158.69827 | 14.5866 | 332.63064 | 2.84934 | 226.03259 | 12.6704 | 377.20832 |
| 4.105 | 137.26536 |  |  | 4.17217 | 175.13272 | 14.7669 | 332.40036 | 3.04445 | 238.25017 | 12.8771 | 377.18285 |
| 4.22074 | 155.28248 |  |  | 4.38381 | 194.70751 | 14.9965 | 332.01905 | 3.21641 | 249.88038 | 13.0877 | 377.08775 |
| 4.45223 | 175.62438 |  |  | 4.59546 | 208.10629 |  |  | 3.48758 | 262.9203 | 13.306 | 376.91565 |
| 4.72009 | 191.41358 |  |  | 4.92285 | 223.2846 |  |  | 3.65954 | 272.43592 | 13.5185 | 376.67774 |
| 5.04748 | 207.20277 |  |  | 5.25024 | 239.09098 |  |  | 3.85135 | 283.36127 | 13.7234 | 376.38542 |
| 5.41456 | 225.21988 |  |  | 5.69006 | 251.96637 |  |  | 4.08283 | 292.2895 | 13.9226 | 376.04603 |
| 5.83785 | 240.42788 |  |  | 5.96123 | 262.22481 |  |  | 4.3507 | 303.91971 | 14.1351 | 375.63003 |
| 6.16523 | 252.82675 |  |  | 6.44405 | 275.6236 |  |  | 4.60202 | 312.14308 | 14.3533 | 375.15358 |
| 6.64805 | 265.22563 |  |  | 6.78797 | 287.24286 |  |  | 4.90957 | 321.65871 | 14.5676 | 374.64806 |
| 7.03166 | 275.97777 |  |  | 7.15504 | 295.72177 |  |  | 5.18074 | 330.58694 | 14.765 | 374.16093 |
| 7.3392 | 284.40513 |  |  | 7.61802 | 304.30537 |  |  | 5.41223 | 338.81032 | 14.9717 | 373.64262 |
| 7.80218 | 289.44218 |  |  | 8.11737 | 312.2609 |  |  | 5.66025 | 345.62397 |  |  |
| 8.32137 | 294.57609 |  |  | 8.58034 | 318.33222 |  |  | 6.02732 | 352.43763 |  |  |
| 8.91993 | 297.38521 |  |  | 9.02347 | 322.62402 |  |  | 6.43077 | 355.1396 |  |  |
| 9.5747 | 300.19433 |  |  | 9.4666 | 324.40355 |  |  | 6.89374 | 359.95614 |  |  |
| 10.22618 | 301.84105 |  |  | 10.11808 | 326.18307 |  |  | 7.22113 | 363.36297 |  |  |
| 10.86442 | 303.58464 |  |  | 10.75632 | 328.06728 |  |  | 7.52868 | 366.06494 |  |  |
| 11.28933 | 303.11192 |  |  | 11.18123 | 327.55643 |  |  | 8.06771 | 370.17663 |  |  |
| 11.73976 | 306.54265 |  |  | 11.63166 | 331.26384 |  |  | 8.62659 | 373.58345 |  |  |
| 12.57742 | 306.39376 |  |  | 12.17831 | 333.51053 |  |  | 9.22184 | 375.69804 |  |  |
| 13.6158 | 310.36528 |  |  | 12.89261 | 333.51053 |  |  | 9.75326 | 371.76692 |  |  |
| 14.65419 | 308.04049 |  |  | 13.45149 | 334.1386 |  |  | 10.20368 | 374.80744 |  |  |
| 15.42471 | 310.36528 |  |  | 14.06658 | 332.88246 |  |  | 10.80257 | 374.99318 |  |  |
|  |  |  |  | 14.76104 | 336.0228 |  |  | 11.30192 | 376.4029 |  |  |
|  |  |  |  | 15.20087 | 334.1386 |  |  | 11.58962 | 376.4029 |  |  |
|  |  |  |  |  |  |  |  | 11.95669 | 376.4029 |  |  |
|  |  |  |  |  |  |  |  | 12.22786 | 372.29121 |  |  |
|  |  |  |  |  |  |  |  | 12.57179 | 377.69514 |  |  |
|  |  |  |  |  |  |  |  | 13.01492 | 377.10776 |  |  |
|  |  |  |  |  |  |  |  | 13.42167 | 374.28831 |  |  |
|  |  |  |  |  |  |  |  | 13.72922 | 378.4 |  |  |
|  |  |  |  |  |  |  |  | 14.13267 | 377.10776 |  |  |
|  |  |  |  |  |  |  |  | 14.42037 | 377.10776 |  |  |
|  |  |  |  |  |  |  |  | 14.78745 | 374.28831 |  |  |
|  |  |  |  |  |  |  |  | 15.15121 | 374.99318 |  |  |
