# Supplementary material for: Material composition and constitutive model development of red mud-based filler for highway tunnel invert filling applications: A comprehensive study
Source: PLoS One. 2025 Apr 16;20(4):e0321926. doi: 10.1371/journal.pone.0321926 (PMC12002488; doi:10.1371/journal.pone.0321926)
Supplement: S9 Table — Test curve and fitting curve results. (DOCX) [file pone.0321926.s009.docx]

Table S9. The (σ1-σ3)-ε1 curves of MRM with confining pressure under different ages (Fig.14). Test curve and fitting curve results.

(a) 7d

| 30kPa | | Fitting data | | 60kPa | | Fitting data | | 90kPa | | Fitting data | |
| --- | --- | --- | --- | --- | --- | --- | --- | --- | --- | --- | --- |
| ε_1_ | (σ_1_-σ_3_) | ε_1_ | (σ_1_-σ_3_) | ε_1_ | (σ_1_-σ_3_) | ε_1_ | (σ_1_-σ_3_) | ε_1_ | (σ_1_-σ_3_) | ε_1_ | (σ_1_-σ_3_) |
| 0 | 0.2223 | 3.7851 | 117.19942 | 0.0017 | -2.9 | 3.4001 | 107.05903 | 0 | 1.3112 | 0.8632 | 103.50521 |
| 0.0019 | 0.4645 | 3.9901 | 177.87754 | 0 | -1.6 | 3.6335 | 186.42631 | 0.0058 | 1.0567 | 1.0815 | 178.70644 |
| 0.0019 | 0.2871 | 4.1987 | 234.45103 | 0 | -2.6 | 3.8212 | 244.44921 | 0.0095 | 1.0475 | 1.2731 | 240.02711 |
| 0.0019 | 0.5099 | 4.3999 | 284.38952 | 0 | 1.1 | 4.0376 | 305.38794 | 0.0095 | 0.8638 | 1.4951 | 305.87992 |
| 0.0019 | 0.722 | 4.6105 | 332.11089 | -0.0019 | 1.8 | 4.2463 | 358.54691 | 0.0132 | 0.2591 | 1.6868 | 358.47885 |
| 0.0076 | 0.529 | 4.804 | 372.12213 | -0.0019 | 4.4 | 4.4587 | 407.42083 | 0.0114 | 1.3179 | 1.8955 | 411.48218 |
| 0.0095 | 1.0034 | 5.0165 | 412.11733 | -0.0019 | 5.4 | 4.6524 | 447.73997 | 0.0132 | 0.5444 | 2.1023 | 459.85102 |
| 0.0132 | 0.9784 | 5.2404 | 450.10678 | -0.0019 | 1.7 | 4.8781 | 490.00798 | 0.0171 | 1.832 | 2.328 | 508.18942 |
| 0.0209 | 1.2021 | 5.4663 | 484.44733 | -0.0019 | 0.8 | 5.0565 | 520.1052 | 0.0171 | 1.0465 | 2.5405 | 549.70176 |
| 0.0265 | 1.8452 | 5.6862 | 514.33209 | 0.0039 | 1.8 | 5.2614 | 551.35398 | 0.0209 | 1.8114 | 2.7492 | 586.92278 |
| 0.0285 | 1.4944 | 5.8627 | 535.98094 | 0.0058 | 0.7 | 5.4795 | 581.03378 | 0.0228 | 1.3142 | 2.9655 | 622.01303 |
| 0.0304 | 2.04 | 6.0809 | 560.0873 | 0.0058 | 2 | 5.694 | 606.93893 | 0.0228 | 1.8284 | 3.1439 | 648.44232 |
| 0.0304 | 1.4499 | 6.2859 | 580.26391 | 0.0115 | 1.6 | 5.8931 | 628.33179 | 0.0247 | 1.7778 | 3.3659 | 678.35597 |
| 0.0304 | 1.4987 | 6.4965 | 598.70565 | 0.0229 | 12.5 | 6.1019 | 648.2741 | 0.0285 | 2.6579 | 3.5728 | 703.45192 |
| 0.0323 | 1.7994 | 6.709 | 615.16448 | 0.0343 | 13.6 | 6.3257 | 667.08525 | 0.0323 | 2.4017 | 3.7852 | 726.61286 |
| 0.0323 | 1.7783 | 6.9195 | 629.5271 | 0.0438 | 3.7 | 6.5401 | 682.86824 | 0.0361 | 3.6283 | 3.9977 | 747.33007 |
| 0.0341 | 2.5738 | 7.1206 | 641.60493 | 0.0551 | 4.4 | 6.7489 | 696.35209 | 0.0361 | 1.8266 | 4.1894 | 764.06085 |
| 0.0341 | 2.3067 | 7.3674 | 654.44529 | 0.0589 | 4.6 | 6.9633 | 708.4642 | 0.0417 | 2.8958 | 4.398 | 780.30816 |
| 0.0341 | 2.0983 | 7.5362 | 662.08796 | 0.0626 | 5.4 | 7.1758 | 718.92373 | 0.0436 | 3.3848 | 4.6371 | 796.61576 |
| 0.0379 | 2.57 | 7.7468 | 670.45331 | 0.076 | 4.6 | 7.3845 | 727.86634 | 0.0475 | 2.5924 | 4.8135 | 807.18688 |
| 0.0379 | 2.3014 | 7.9631 | 677.82419 | 0.0969 | 4.6 | 7.5989 | 735.83919 | 0.0513 | 3.3237 | 5.0318 | 818.6955 |
| 0.0417 | 2.5151 | 8.1529 | 683.37869 | 0.1252 | 4.4 | 7.8076 | 742.55829 | 0.0531 | 2.6494 | 5.231 | 827.80209 |
| 0.0455 | 2.8081 | 8.3767 | 688.95161 | 0.1575 | 4.2 | 7.9859 | 747.57959 | 0.0531 | 2.3889 | 5.453 | 836.52536 |
| 0.0455 | 2.7855 | 8.5816 | 693.23071 | 0.1633 | 2.6 | 8.2023 | 752.8909 | 0.057 | 2.8622 | 5.6465 | 843.01546 |
| 0.0455 | 3.0006 | 8.796 | 696.96731 | 0.1784 | 3.9 | 8.4299 | 757.66867 | 0.0589 | 2.8431 | 5.8627 | 849.16152 |
| 0.0494 | 3.047 | 9.0085 | 700.01541 | 0.1898 | 3.5 | 8.6367 | 761.38944 | 0.0607 | 2.8904 | 6.0771 | 854.22248 |
| 0.0494 | 3.0164 | 9.204 | 702.31652 | 0.1974 | 2.4 | 8.8321 | 764.43634 | 0.0607 | 2.6435 | 6.2745 | 858.07099 |
| 0.0513 | 2.9975 | 9.3975 | 704.18016 | 0.2164 | 2.5 | 9.0446 | 767.30313 | 0.0589 | 3.111 | 6.4965 | 861.57816 |
| 0.055 | 3.2998 | 9.6119 | 705.8286 | 0.2259 | 2.6 | 9.1984 | 769.1246 | 0.0607 | 3.3418 | 6.7128 | 864.26323 |
| 0.0587 | 4.0833 | 9.8186 | 707.06242 | 0.2354 | 2.7 | 9.4506 | 771.71526 | 0.0626 | 3.0714 | 6.9006 | 866.08539 |
| 0.0626 | 3.8775 | 10.0274 | 708.00876 | 0.2543 | 3.4 | 9.6745 | 773.66131 | 0.0645 | 3.1013 | 7.1113 | 867.64458 |
| 0.0645 | 4.0868 | 10.2456 | 708.72761 | 0.2656 | 2.5 | 9.8719 | 775.14235 | 0.0626 | 3.1191 | 7.3161 | 868.74086 |
| 0.0626 | 3.2563 | 10.441 | 709.17637 | 0.2733 | 0.7 | 10.0881 | 776.54678 | 0.0645 | 3.8196 | 7.5286 | 869.51446 |
| 0.0645 | 3.2477 | 10.6593 | 709.49893 | 0.2885 | -0.5 | 10.2874 | 777.66498 | 0.0645 | 3.5565 | 7.7487 | 869.99833 |
| 0.0645 | 3.8123 | 10.8792 | 709.6693 | 0.3055 | -0.6 | 10.4922 | 778.65799 | 0.0645 | 3.6241 | 7.9555 | 870.22116 |
| 0.0645 | 4.6257 | 11.0822 | 709.71692 | 0.3131 | 0.5 | 10.6954 | 779.50223 | 0.0645 | 3.057 | 8.17 | 870.27331 |
| 0.0645 | 4.3739 | 11.2986 | 709.67668 | 0.3169 | -0.2 | 10.9267 | 780.30654 | 0.0645 | 3.5495 | 8.3654 | 870.20705 |
| 0.0664 | 4.3695 | 11.4979 | 709.57489 | 0.3187 | 2.5873 | 11.1051 | 780.81976 | 0.0645 | 3.7987 | 8.5911 | 870.04453 |
| 0.2485 | 8.4947 | 11.6875 | 709.43359 | 0.4706 | 8.686 | 11.3443 | 781.36724 | 0.2524 | 11.0757 | 8.7923 | 869.86021 |
| 0.4591 | 10.2549 | 11.9209 | 709.21452 | 0.7173 | 9.5087 | 11.551 | 781.71272 | 0.4364 | 16.1269 | 8.9991 | 869.66533 |
| 0.6679 | 13.2873 | 12.124 | 708.99346 | 0.9051 | 9.1073 | 11.7465 | 781.93047 | 0.666 | 25.7951 | 9.223 | 869.48029 |
| 0.85 | 15.8624 | 12.3289 | 708.74895 | 1.1327 | 11.1981 | 11.9609 | 782.04564 | 0.8667 | 108.53541 | 9.4165 | 869.36419 |
| 1.1043 | 17.8062 | 12.5281 | 708.49508 | 1.313 | 15.8558 | 12.1676 | 782.0314 | 0.98234 | 155.90258 | 9.6005 | 869.30613 |
| 1.2769 | 17.0807 | 12.7595 | 708.18378 | 1.5292 | 18.5962 | 12.3555 | 781.90893 | 1.16406 | 201.54825 | 9.8282 | 869.3198 |
| 1.4838 | 11.3609 | 12.9474 | 707.91941 | 1.7494 | 19.645 | 12.5812 | 781.62037 | 1.27836 | 241.88433 | 10.0482 | 869.43385 |
| 1.6924 | 13.7066 | 13.1485 | 707.62521 | 1.9429 | 25.8746 | 12.7937 | 781.20464 | 1.48736 | 294.73851 | 10.2305 | 869.60768 |
| 1.903 | 15.6408 | 13.3572 | 707.30693 | 2.146 | 29.5913 | 13.0062 | 780.64746 | 1.65718 | 351.24962 | 10.4544 | 869.91985 |
| 2.0832 | 12.5403 | 13.5849 | 706.94313 | 2.3831 | 30.9294 | 13.2074 | 779.98968 | 1.77148 | 373.67205 | 10.6669 | 870.31221 |
| 2.3394 | 16.0364 | 13.7936 | 706.59281 | 2.5064 | 32.0954 | 13.4047 | 779.22376 | 1.98048 | 424.09215 | 10.866 | 870.75642 |
| 2.5537 | 15.5548 |  |  | 2.7986 | 37.6391 | 13.6096 | 778.30633 | 2.16989 | 466.51458 | 11.0729 | 871.28398 |
| 2.7492 | 15.7249 |  |  | 2.9751 | 38.4801 | 13.824 | 777.22191 | 2.37889 | 515.19606 | 11.2721 | 871.83975 |
| 2.9731 | 15.9515 |  |  | 3.1933 | 48.4347 |  |  | 2.58789 | 561.79119 | 11.5016 | 872.5133 |
| 3.142 | 10.6958 |  |  | 3.394 | 98.15702 |  |  | 2.79689 | 604.21362 | 11.7218 | 873.16228 |
| 3.3905 | 19.901 |  |  | 3.50964 | 140.99484 |  |  | 3.04508 | 644.89743 | 11.8983 | 873.66014 |
| 3.584 | 21.3216 |  |  | 3.65217 | 184.16262 |  |  | 3.3292 | 683.14716 | 12.1277 | 874.24075 |
| 3.85142 | 115.65489 |  |  | 3.78607 | 229.76145 |  |  | 3.69168 | 717.57192 | 12.3119 | 874.62103 |
| 3.90898 | 146.72384 |  |  | 3.91669 | 271.90106 |  |  | 3.9758 | 743.99901 | 12.5433 | 874.94232 |
| 4.00369 | 186.97482 |  |  | 4.01466 | 310.26696 |  |  | 4.35788 | 774.25107 | 12.7501 | 875.03434 |
| 4.13758 | 235.56609 |  |  | 4.16488 | 350.51972 |  |  | 4.7171 | 798.59181 | 12.938 | 874.91582 |
| 4.34658 | 285.97046 |  |  | 4.33796 | 389.2001 |  |  | 5.0404 | 818.75985 | 13.1694 | 874.44565 |
| 4.55558 | 332.386 |  |  | 4.46859 | 420.3331 |  |  | 5.45841 | 833.01657 | 13.3744 | 873.67145 |
| 4.7254 | 366.10979 |  |  | 4.60248 | 453.03846 |  |  | 5.99071 | 851.09826 | 13.5869 | 872.44985 |
| 4.99318 | 412.52533 |  |  | 4.84414 | 488.88857 |  |  | 6.42831 | 859.09594 | 13.7917 | 870.80586 |
| 5.163 | 440.08456 |  |  | 5.04008 | 520.96499 |  |  | 6.86591 | 867.44133 | 14.0024 | 868.56785 |
| 5.372 | 469.4569 |  |  | 5.28827 | 550.21113 |  |  | 7.2088 | 871.26631 | 14.2015 | 868.56785 |
| 5.62019 | 507.53214 |  |  | 5.57238 | 583.23097 |  |  | 7.78029 | 867.44133 | 14.4026 | 868.56785 |
| 6.0186 | 545.60739 |  |  | 5.8565 | 617.82318 |  |  | 8.21789 | 867.44133 | 14.6133 | 868.56785 |
| 6.32231 | 573.16662 |  |  | 6.21898 | 648.95617 |  |  | 8.46282 | 867.44133 | 14.8296 | 868.56785 |
| 6.59009 | 598.18749 |  |  | 6.5031 | 672.85625 |  |  | 9.01471 | 869.17996 | 15.0307 | 868.56785 |
| 6.8742 | 621.39526 |  |  | 6.88518 | 700.21554 |  |  | 9.60253 | 871.26631 | 15.2318 | 868.56785 |
| 7.1975 | 648.95449 |  |  | 7.2444 | 722.22877 |  |  | 10.04013 | 871.26631 |  |  |
| 7.6547 | 676.51372 |  |  | 7.5677 | 740.4683 |  |  | 10.51692 | 871.26631 |  |  |
| 8.0923 | 691.01857 |  |  | 7.98571 | 753.36176 |  |  | 10.89574 | 871.26631 |  |  |
| 8.52989 | 705.88605 |  |  | 8.51801 | 769.71444 |  |  | 11.46723 | 879.6117 |  |  |
| 8.96749 | 714.22634 |  |  | 8.95561 | 776.94736 |  |  | 11.73501 | 871.26631 |  |  |
| 9.42034 | 710.88128 |  |  | 9.39321 | 784.49475 |  |  | 12.24772 | 871.26631 |  |  |
| 9.86514 | 708.41146 |  |  | 9.7361 | 787.95397 |  |  | 12.64613 | 871.26631 |  |  |
| 10.30994 | 709.39939 |  |  | 10.30759 | 784.49475 |  |  | 13.02495 | 871.26631 |  |  |
| 10.75475 | 709.39939 |  |  | 10.74519 | 784.49475 |  |  | 13.48214 | 871.26631 |  |  |
| 11.19955 | 709.39939 |  |  | 10.99012 | 784.49475 |  |  | 14.01444 | 871.26631 |  |  |
| 11.64435 | 713.3511 |  |  | 11.54201 | 786.06713 |  |  | 14.52715 | 871.26631 |  |  |
| 12.08915 | 712.36317 |  |  | 12.12983 | 787.95397 |  |  | 15.03986 | 871.26631 |  |  |
| 12.53395 | 712.36317 |  |  | 12.56743 | 787.95397 |  |  |  |  |  |  |
| 12.97876 | 712.36317 |  |  | 13.04422 | 787.95397 |  |  |  |  |  |  |
| 13.42356 | 712.36317 |  |  | 13.50141 | 786.06713 |  |  |  |  |  |  |
| 13.86836 | 712.36317 |  |  | 13.97493 | 789.84082 |  |  |  |  |  |  |

(b) 14d

| 30kPa | | Fitting data | | 60kPa | | Fitting data | | 90kPa | | Fitting data | |
| --- | --- | --- | --- | --- | --- | --- | --- | --- | --- | --- | --- |
| ε_1_ | (σ_1_-σ_3_) | ε_1_ | (σ_1_-σ_3_) | ε_1_ | (σ_1_-σ_3_) | ε_1_ | (σ_1_-σ_3_) | ε_1_ | (σ_1_-σ_3_) | ε_1_ | (σ_1_-σ_3_) |
| 0 | 1.0304 | 3.8365 | 134.42916 | 0.002 | 1.6009 | 3.8212 | 129.50859 | -0.0019 | 3.1325 | 1.9789 | 136.76374 |
| 0.002 | 1.023 | 4.0167 | 184.06001 | 0 | 1.6061 | 4.0376 | 195.69709 | 0 | 3.3791 | 2.1705 | 197.28968 |
| 0.0039 | 1.0153 | 4.2197 | 234.84932 | 0 | 1.8524 | 4.2463 | 253.76877 | 0 | 2.954 | 2.3679 | 255.35704 |
| 0.0039 | 1.0011 | 4.436 | 283.4538 | 0 | 1.5948 | 4.4587 | 307.4731 | -0.0019 | 2.6931 | 2.5785 | 312.75562 |
| 0.0039 | 0.9903 | 4.6295 | 322.50357 | -0.0019 | 1.5878 | 4.6524 | 352.03534 | -0.0019 | 2.9262 | 2.8175 | 372.52716 |
| 0.0039 | 0.9777 | 4.8325 | 359.34488 | -0.0019 | 1.5758 | 4.8781 | 399.03879 | -0.0039 | 2.6569 | 2.9864 | 411.51812 |
| 0.0076 | 0.9594 | 5.0564 | 395.50167 | -0.0019 | 1.8095 | 5.0565 | 432.71201 | -0.0039 | 2.6387 | 3.2216 | 461.59706 |
| 0.0076 | 1.8535 | 5.2556 | 424.07486 | -0.0019 | 1.5408 | 5.2614 | 467.87875 | -0.0058 | 2.868 | 3.419 | 500.05256 |
| 0.0114 | 2.0789 | 5.4739 | 451.86728 | -0.0019 | 1.6073 | 5.4795 | 501.50024 | -0.0076 | 2.9278 | 3.6068 | 533.78986 |
| 0.0134 | 1.8114 | 5.6825 | 475.31232 | 0.0039 | 1.8326 | 5.694 | 531.0472 | -0.0076 | 2.9045 | 3.8535 | 574.14943 |
| 0.0134 | 1.2086 | 5.8627 | 493.35048 | 0.0058 | 1.565 | 5.8931 | 555.60954 | -0.0058 | 3.1231 | 4.0602 | 604.72642 |
| 0.0152 | 2.1021 | 6.0848 | 513.03786 | 0.0058 | 1.5424 | 6.1019 | 578.65678 | -0.0076 | 3.1773 | 4.2785 | 634.03427 |
| 0.0152 | 2.0779 | 6.2916 | 529.10039 | 0.0115 | 1.5972 | 6.3257 | 600.54785 | -0.0076 | 3.1505 | 4.4834 | 658.93836 |
| 0.019 | 1.8005 | 6.5136 | 544.18275 | 0.0229 | 1.814 | 6.5401 | 619.04319 | -0.0114 | 3.1154 | 4.6788 | 680.49541 |
| 0.0247 | 2.5236 | 6.7222 | 556.54924 | 0.0343 | 1.6112 | 6.7489 | 634.9484 | -0.0114 | 3.4277 | 4.8856 | 701.13579 |
| 0.0266 | 2.0728 | 6.9386 | 567.75236 | 0.0438 | 1.8302 | 6.9633 | 649.32614 | -0.0152 | 3.3857 | 5.0944 | 719.86486 |
| 0.0266 | 2.3024 | 7.134 | 576.6172 | 0.0551 | 1.7909 | 7.1758 | 661.81726 | -0.0152 | 3.6856 | 5.3125 | 737.3306 |
| 0.0285 | 2.5288 | 7.3256 | 584.2973 | 0.0589 | 1.8425 | 7.3845 | 672.55537 | -0.038 | 3.4154 | 5.5308 | 752.83223 |
| 0.0323 | 2.8222 | 7.5534 | 592.29192 | 0.0626 | 1.8064 | 7.5989 | 682.17555 | -0.0417 | 3.3846 | 5.7394 | 765.94159 |
| 0.0323 | 2.302 | 7.7639 | 598.73221 | 0.076 | 1.8499 | 7.8076 | 690.31578 | -0.0513 | 3.1869 | 5.9462 | 777.42732 |
| 0.0343 | 2.3416 | 7.9897 | 604.78107 | 0.0969 | 1.7966 | 7.9859 | 696.41638 | -0.0531 | 3.394 | 6.1454 | 787.18391 |
| 0.0343 | 2.5696 | 8.1719 | 609.10853 | 0.1252 | 1.8207 | 8.2023 | 702.88097 | -0.055 | 3.3537 | 6.3428 | 795.69087 |
| 0.038 | 3.0332 | 8.3767 | 613.47201 | 0.1575 | 2.6717 | 8.4299 | 708.69955 | -0.0607 | 3.4001 | 6.559 | 803.79545 |
| 0.0417 | 3.4093 | 8.595 | 617.62999 | 0.1633 | 2.6217 | 8.6367 | 713.22579 | -0.0626 | 3.4299 | 6.7582 | 810.24048 |
| 0.0417 | 3.0612 | 8.8094 | 621.30185 | 0.1784 | 2.6517 | 8.8321 | 716.9223 | -0.0664 | 3.6393 | 6.9651 | 815.9938 |
| 0.0475 | 3.3527 | 9.001 | 624.29695 | 0.1898 | 2.8443 | 9.0446 | 720.38504 | -0.0684 | 3.6769 | 7.1681 | 820.79562 |
| 0.0513 | 3.3311 | 9.2249 | 627.5149 | 0.1974 | 2.8806 | 9.1984 | 722.57347 | -0.0703 | 3.6271 | 7.3616 | 824.67167 |
| 0.0531 | 2.802 | 9.4165 | 630.06879 | 0.2164 | 2.8929 | 9.4506 | 725.66402 | -0.0759 | 3.668 | 7.595 | 828.53345 |
| 0.0531 | 3.5882 | 9.6499 | 632.97589 | 0.2259 | 2.8501 | 9.6745 | 727.96388 | -0.0759 | 3.6051 | 7.8207 | 831.51922 |
| 0.0531 | 3.3892 | 9.8586 | 635.41779 | 0.2354 | 2.8726 | 9.8719 | 729.69998 | -0.0759 | 3.8863 | 8.0067 | 833.49291 |
| 0.057 | 3.3646 | 10.0369 | 637.40414 | 0.2543 | 3.1332 | 10.0881 | 731.33595 | -0.0759 | 3.9106 | 8.2344 | 835.38508 |
| 0.0645 | 3.8197 | 10.2532 | 639.70571 | 0.2656 | 3.0856 | 10.2874 | 732.63496 | -0.0759 | 3.8803 | 8.4487 | 836.70743 |
| 0.0645 | 3.889 | 10.4734 | 641.93835 | 0.2733 | 3.3829 | 10.4922 | 733.7926 | -0.0759 | 3.8478 | 8.6518 | 837.60846 |
| 0.0721 | 4.116 | 10.6915 | 644.04593 | 0.2885 | 3.3522 | 10.6954 | 734.78974 | -0.0759 | 4.2417 | 8.8377 | 838.17539 |
| 0.076 | 4.1 | 10.8775 | 645.76235 | 0.3055 | 3.6432 | 10.9267 | 735.76853 | -0.0759 | 3.8951 | 9.0408 | 838.55512 |
| 0.0779 | 3.3481 | 11.1014 | 647.72628 | 0.3131 | 3.6061 | 11.1051 | 736.42522 | -0.0759 | 4.4516 | 9.259 | 838.73005 |
| 0.0797 | 4.0725 | 11.3005 | 649.37297 | 0.3169 | 3.8462 | 11.3443 | 737.18872 | -0.0759 | 4.1852 | 9.4544 | 838.71906 |
| 0.0816 | 4.0604 | 11.5111 | 651.00392 | 0.3187 | 3.5708 | 11.551 | 737.75194 | -0.0759 | 4.1746 | 9.6764 | 838.55397 |
| 0.2619 | 6.3666 | 11.7122 | 652.44525 | 0.4706 | 5.3761 | 11.7465 | 738.20939 | 0.1139 | 11.7253 | 9.904 | 838.25578 |
| 0.4857 | 8.9729 | 11.8944 | 653.6445 | 0.7173 | 6.6807 | 11.9609 | 738.63186 | 0.3149 | 17.9279 | 10.109 | 837.90626 |
| 0.666 | 8.6852 | 12.124 | 654.99875 | 0.9051 | 7.3892 | 12.1676 | 738.96337 | 0.5141 | 25.1195 | 10.3157 | 837.50142 |
| 0.8899 | 9.6688 | 12.3497 | 656.14542 | 1.1327 | 8.2228 | 12.3555 | 739.20097 | 0.7285 | 32.9787 | 10.5245 | 837.06064 |
| 1.1024 | 8.8705 | 12.5548 | 657.01736 | 1.313 | 9.2877 | 12.5812 | 739.40575 | 0.9467 | 35.9758 | 10.7331 | 836.60698 |
| 1.2902 | 9.5788 | 12.7653 | 657.73472 | 1.5292 | 9.9652 | 12.7937 | 739.51684 | 1.1574 | 41.0068 | 10.9476 | 836.14312 |
| 1.499 | 10.1284 | 12.9589 | 658.23004 | 1.7494 | 11.7296 | 13.0062 | 739.54692 | 1.3395 | 43.8018 | 11.1468 | 835.72677 |
| 1.7057 | 10.855 | 13.1809 | 658.60042 | 1.9429 | 13.7064 | 13.2074 | 739.49925 | 1.5615 | 58.9569 | 11.3346 | 835.35514 |
| 1.9373 | 12.6266 | 13.3744 | 658.75127 | 2.146 | 16.2531 | 13.4047 | 739.37967 | 1.7759 | 67.5242 | 11.5661 | 834.93336 |
| 2.127 | 12.8078 | 13.6039 | 658.72795 | 2.3831 | 19.9732 | 13.6096 | 739.17906 | 1.90578 | 98.36001 | 11.7673 | 834.60461 |
| 2.3319 | 13.8547 | 13.7994 | 658.5443 | 2.5064 | 20.9853 |  |  | 2.0479 | 155.82805 | 11.9684 | 834.31408 |
| 2.5576 | 14.7602 |  |  | 2.7986 | 25.1302 |  |  | 2.21817 | 212.19029 | 12.1751 | 834.05608 |
| 2.7759 | 17.7381 |  |  | 2.9751 | 25.911 |  |  | 2.33277 | 245.57684 | 12.3801 | 833.84008 |
| 2.9789 | 18.4505 |  |  | 3.1933 | 29.7835 |  |  | 2.40808 | 275.01445 | 12.6172 | 833.63653 |
| 3.1933 | 23.1805 |  |  | 3.4001 | 31.98 |  |  | 2.50304 | 306.24703 | 12.8108 | 833.50306 |
| 3.3981 | 28.1347 |  |  | 3.6335 | 40.3847 |  |  | 2.61764 | 335.32564 | 13.0271 | 833.3828 |
| 3.5803 | 35.9199 |  |  | 3.82582 | 105.57122 |  |  | 2.79119 | 362.60928 | 13.2358 | 833.28866 |
| 3.77262 | 92.22358 |  |  | 3.96407 | 173.86023 |  |  | 2.8665 | 391.68789 | 13.4218 | 833.21634 |
| 3.91087 | 151.87864 |  |  | 4.15139 | 230.76774 |  |  | 3.02039 | 421.12549 | 13.6531 | 833.13176 |
| 4.09819 | 201.59118 |  |  | 4.30749 | 295.26292 |  |  | 3.15137 | 448.05013 | 13.8637 | 833.04955 |
| 4.25429 | 257.93207 |  |  | 4.5702 | 352.07071 |  |  | 3.36093 | 485.74463 | 14.0667 | 832.95543 |
| 4.41485 | 304.33045 |  |  | 4.89437 | 400.53098 |  |  | 3.57049 | 517.3362 | 14.2736 | 832.83414 |
| 4.69054 | 358.16285 |  |  | 5.27747 | 459.01752 |  |  | 3.79232 | 549.72931 | 14.4804 | 832.67671 |
| 5.01471 | 389.3046 |  |  | 5.60164 | 507.4778 |  |  | 3.9841 | 584.93905 | 14.691 | 832.46803 |
| 5.35852 | 426.28543 |  |  | 5.9258 | 549.81091 |  |  | 4.17142 | 618.1927 | 14.8788 | 832.1993 |
| 5.69906 | 471.53829 |  |  | 6.19103 | 588.24492 |  |  | 4.40779 | 645.57805 | 15.0894 | 832.11709 |
| 6.02322 | 497.32756 |  |  | 6.51519 | 617.7667 |  |  | 4.63525 | 672.9634 |  |  |
| 6.44235 | 533.8218 |  |  | 6.93432 | 643.38937 |  |  | 4.84487 | 703.2829 |  |  |
| 6.82545 | 556.69152 |  |  | 7.31742 | 669.56906 |  |  | 5.17044 | 731.6463 |  |  |
| 7.33953 | 576.15511 |  |  | 7.7169 | 689.06458 |  |  | 5.4648 | 757.56458 |  |  |
| 7.71936 | 593.18576 |  |  | 8.19496 | 698.53382 |  |  | 5.86619 | 782.50481 |  |  |
| 8.21707 | 607.29687 |  |  | 8.57563 | 724.72492 |  |  | 6.39302 | 796.99346 |  |  |
| 8.57725 | 624.32751 |  |  | 9.02163 | 728.51875 |  |  | 6.90711 | 811.71226 |  |  |
| 8.96843 | 636.41026 |  |  | 9.46762 | 728.51875 |  |  | 7.46048 | 822.12312 |  |  |
| 9.41442 | 636.41026 |  |  | 9.91361 | 730.79505 |  |  | 7.99421 | 830.38001 |  |  |
| 9.86041 | 638.39876 |  |  | 10.3596 | 734.58889 |  |  | 8.50829 | 834.68795 |  |  |
| 10.3064 | 641.71293 |  |  | 10.80559 | 730.03629 |  |  | 8.9667 | 838.9959 |  |  |
| 10.75239 | 637.73593 |  |  | 11.25159 | 739.14149 |  |  | 9.33016 | 840.79087 |  |  |
| 11.19839 | 645.68994 |  |  | 11.69758 | 740.65902 |  |  | 9.67942 | 840.20967 |  |  |
| 11.64438 | 647.01561 |  |  | 12.14357 | 739.14149 |  |  | 10.12541 | 838.74259 |  |  |
| 12.09037 | 645.68994 |  |  | 12.58956 | 741.41779 |  |  | 10.57141 | 836.7865 |  |  |
| 12.53636 | 647.67844 |  |  | 13.03555 | 745.21162 |  |  | 11.0174 | 834.34138 |  |  |
| 12.98235 | 650.99261 |  |  | 13.48155 | 742.93532 |  |  | 11.46339 | 834.34138 |  |  |
| 13.42835 | 649.00411 |  |  |  |  |  |  | 11.90938 | 833.85235 |  |  |
| 13.87434 | 649.00411 |  |  |  |  |  |  | 12.35537 | 833.85235 |  |  |
|  |  |  |  |  |  |  |  | 12.80137 | 833.85235 |  |  |
|  |  |  |  |  |  |  |  | 13.24736 | 831.89626 |  |  |
|  |  |  |  |  |  |  |  | 13.69335 | 835.31942 |  |  |
|  |  |  |  |  |  |  |  | 14.13934 | 834.34138 |  |  |
|  |  |  |  |  |  |  |  | 14.58533 | 831.89626 |  |  |
|  |  |  |  |  |  |  |  | 15.03133 | 831.89626 |  |  |

(c) 28d

| 30kPa | | Fitting data | | 60kPa | | Fitting data | | 90kPa | | Fitting data | |
| --- | --- | --- | --- | --- | --- | --- | --- | --- | --- | --- | --- |
| ε_1_ | (σ_1_-σ_3_) | ε_1_ | (σ_1_-σ_3_) | ε_1_ | (σ_1_-σ_3_) | ε_1_ | (σ_1_-σ_3_) | ε_1_ | (σ_1_-σ_3_) | ε_1_ | (σ_1_-σ_3_) |
| 0 | 2.3863 | 3.1989 | 61.99774 | -0.0019 | 1.3017 | 3.1116 | 82.26077 | 0 | 1.3703 | 1.2199 | 117.5874 |
| 0.0038 | 2.125 | 3.4 | 128.88705 | 0 | 1.2959 | 3.3241 | 158.95115 | 0 | 1.6147 | 1.421 | 182.78021 |
| 0.0038 | 1.6045 | 3.5993 | 188.97535 | 0 | 1.1177 | 3.5271 | 225.27763 | 0.0019 | 1.3576 | 1.6544 | 252.74788 |
| 0.0038 | 1.5826 | 3.8364 | 253.03373 | -0.0019 | 1.3579 | 3.7282 | 284.79044 | 0.0038 | 1.5937 | 1.8214 | 299.25398 |
| 0.0056 | 1.5547 | 4.0431 | 302.81661 | -0.0019 | 1.099 | 3.9389 | 341.02634 | 0.0095 | 1.5869 | 2.0452 | 357.18622 |
| 0.0095 | 2.4423 | 4.2481 | 347.07095 | -0.0019 | 1.086 | 4.1723 | 396.59879 | 0.0114 | 1.3285 | 2.2426 | 404.33408 |
| 0.0095 | 1.5839 | 4.415 | 379.6203 | -0.0019 | 1.0699 | 4.379 | 440.40505 | 0.0228 | 1.312 | 2.4665 | 453.59756 |
| 0.0095 | 2.1375 | 4.6731 | 424.32253 | -0.0019 | 1.048 | 4.582 | 478.90002 | 0.0265 | 1.5495 | 2.6733 | 495.35622 |
| 0.0132 | 2.4274 | 4.8534 | 451.82873 | 0.0019 | 1.3569 | 4.7546 | 508.37994 | 0.036 | 1.2915 | 2.8896 | 535.42767 |
| 0.0114 | 2.145 | 5.081 | 482.59119 | 0.0075 | 1.3266 | 4.9994 | 545.50909 | 0.0455 | 1.6048 | 3.1003 | 571.13795 |
| 0.017 | 2.6174 | 5.2745 | 505.57458 | 0.0114 | 1.2872 | 5.1968 | 571.79963 | 0.0664 | 1.5926 | 3.3033 | 602.63678 |
| 0.019 | 2.4142 | 5.4851 | 527.59246 | 0.0228 | 1.327 | 5.4111 | 597.00754 | 0.0759 | 1.5728 | 3.5232 | 633.7481 |
| 0.019 | 2.1278 | 5.709 | 547.90884 | 0.0323 | 1.3668 | 5.6161 | 618.17726 | 0.0796 | 1.5641 | 3.7093 | 657.78774 |
| 0.019 | 2.1691 | 5.912 | 563.85581 | 0.0398 | 1.3267 | 5.8324 | 637.70206 | 0.0854 | 1.548 | 3.9672 | 687.87717 |
| 0.0228 | 2.6289 | 6.0961 | 576.49103 | 0.0436 | 1.3606 | 6.0411 | 654.08178 | 0.0967 | 1.6158 | 4.1438 | 706.46636 |
| 0.0265 | 2.9151 | 6.3256 | 590.06614 | 0.0569 | 1.3147 | 6.246 | 668.06021 | 0.1195 | 1.8483 | 4.3581 | 726.98317 |
| 0.0265 | 2.6267 | 6.5571 | 601.59294 | 0.0721 | 1.3416 | 6.4509 | 680.17697 | 0.1404 | 1.8265 | 4.5555 | 744.035 |
| 0.0265 | 2.6753 | 6.762 | 610.20286 | 0.091 | 1.2894 | 6.6596 | 690.81562 | 0.1574 | 1.8064 | 4.7641 | 760.26855 |
| 0.0284 | 2.8709 | 6.9594 | 617.256 | 0.11 | 1.302 | 6.8759 | 700.23874 | 0.1745 | 1.7925 | 4.9729 | 774.81454 |
| 0.0304 | 2.6604 | 7.1681 | 623.55023 | 0.129 | 1.3158 | 7.0751 | 707.64607 | 0.203 | 1.8469 | 5.1796 | 787.66357 |
| 0.0341 | 3.1156 | 7.3598 | 628.41043 | 0.165 | 1.5598 | 7.2932 | 714.5344 | 0.222 | 2.0713 | 5.3941 | 799.49447 |
| 0.0398 | 2.9045 | 7.5855 | 633.15565 | 0.184 | 1.5714 | 7.5001 | 720.04053 | 0.2448 | 2.046 | 5.6028 | 809.65456 |
| 0.0474 | 3.1655 | 7.7943 | 636.73845 | 0.2106 | 1.809 | 7.6955 | 724.44369 | 0.2561 | 2.6097 | 5.766 | 816.74297 |
| 0.055 | 3.3691 | 7.9782 | 639.34623 | 0.239 | 1.7969 | 7.8871 | 728.10946 | 0.2731 | 2.6685 | 6.0164 | 826.27942 |
| 0.0569 | 3.1365 | 8.1984 | 641.89699 | 0.2599 | 1.7966 | 8.1091 | 731.6638 | 0.2845 | 2.8931 | 6.2326 | 833.32214 |
| 0.0569 | 3.0936 | 8.3995 | 643.76833 | 0.2846 | 2.0435 | 8.3274 | 734.54383 | 0.3016 | 2.8756 | 6.4395 | 839.12703 |
| 0.0587 | 2.8837 | 8.6234 | 645.42806 | 0.2979 | 2.6142 | 8.5361 | 736.81993 | 0.3035 | 2.8581 | 6.6293 | 843.72043 |
| 0.0626 | 3.4014 | 8.8396 | 646.68528 | 0.3187 | 2.6423 | 8.741 | 738.67601 | 0.3244 | 3.17 | 6.8474 | 848.21442 |
| 0.0683 | 3.3342 | 9.0426 | 647.61722 | 0.3357 | 2.6401 | 8.9496 | 740.2459 | 0.3396 | 3.3919 | 7.0447 | 851.62544 |
| 0.0721 | 3.3689 | 9.2476 | 648.36297 | 0.3471 | 2.6448 | 9.1584 | 741.55142 | 0.3415 | 3.3721 | 7.2763 | 854.91866 |
| 0.0777 | 3.397 | 9.4411 | 648.9233 | 0.3661 | 2.8842 | 9.369 | 742.64853 | 0.3452 | 3.1037 | 7.4679 | 857.12283 |
| 0.0854 | 3.6759 | 9.6669 | 649.43924 | 0.3851 | 3.0908 | 9.5796 | 743.56718 | 0.3585 | 3.4203 | 7.6727 | 859.01439 |
| 0.0949 | 3.7117 | 9.8851 | 649.8289 | 0.3984 | 3.1234 | 9.7599 | 744.23849 | 0.3661 | 3.658 | 7.8625 | 860.38405 |
| 0.0986 | 3.6863 | 10.0919 | 650.12263 | 0.406 | 3.3388 | 9.9989 | 744.99681 | 0.3756 | 3.6495 | 8.0655 | 861.48628 |
| 0.0986 | 3.6674 | 10.2779 | 650.33757 | 0.4136 | 3.3959 | 10.2076 | 745.56019 | 0.3851 | 3.1434 | 8.3008 | 862.35186 |
| 0.1024 | 3.9685 | 10.4827 | 650.5307 | 0.4211 | 3.3722 | 10.3879 | 745.98679 | 0.3965 | 3.3663 | 8.4981 | 862.78069 |
| 0.1081 | 3.6985 | 10.6971 | 650.69178 | 0.4306 | 3.8336 | 10.6099 | 746.44859 | 0.4098 | 3.3668 | 8.7239 | 862.98584 |
| 0.1081 | 3.6873 | 10.9134 | 650.81642 | 0.4345 | 3.8209 | 10.8375 | 746.86067 | 0.4174 | 3.347 | 8.9231 | 862.94955 |
| 0.294 | 5.986 | 11.1221 | 650.90221 | 0.6204 | 5.9946 | 11.0178 | 747.14887 | 0.5939 | 4.2182 | 9.1413 | 862.71248 |
| 0.499 | 8.2087 | 11.3365 | 650.95445 | 0.8064 | 7.168 | 11.2265 | 747.44469 | 0.8139 | 5.1404 | 9.3537 | 862.31617 |
| 0.6981 | 10.0073 | 11.5339 | 650.96847 | 1.0378 | 10.0282 | 11.458 | 747.72946 | 1.0189 | 6.4884 | 9.5398 | 861.85836 |
| 0.8936 | 10.9981 | 11.754 | 650.94266 | 1.237 | 11.7684 | 11.6629 | 747.94653 | 1.13986 | 12.5921 | 9.7485 | 861.24571 |
| 1.1308 | 14.5558 | 11.9436 | 650.88297 | 1.459 | 14.8222 | 11.8621 | 748.12889 | 1.14878 | 101.76535 | 9.9515 | 860.5706 |
| 1.3186 | 17.0962 | 12.1619 | 650.7691 | 1.6659 | 17.904 | 12.0784 | 748.29888 | 1.19338 | 112.68943 | 10.1791 | 859.74541 |
| 1.5444 | 19.345 | 12.3763 | 650.6094 | 1.8708 | 21.116 | 12.2927 | 748.44435 | 1.21231 | 153.01584 | 10.3898 | 858.93828 |
| 1.755 | 24.3746 | 12.5793 | 650.41567 | 2.0795 | 26.2019 | 12.4958 | 748.56879 | 1.34656 | 200.09003 | 10.5776 | 858.19914 |
| 1.9637 | 29.0913 | 12.7785 | 650.18891 | 2.29 | 32.7498 | 12.7045 | 748.69373 | 1.57577 | 243.11879 | 10.7901 | 857.35589 |
| 2.1515 | 30.3294 | 12.9986 | 649.90386 | 2.4987 | 36.1525 | 12.8999 | 748.82078 | 1.72967 | 279.52773 | 11.0064 | 856.50584 |
| 2.383 | 29.1954 |  |  | 2.6847 | 40.5371 |  |  | 1.93818 | 314.679 | 11.2207 | 855.68714 |
| 2.5804 | 35.4391 |  |  | 2.8973 | 55.0691 |  |  | 2.0809 | 356.76076 | 11.4295 | 854.9256 |
| 2.7795 | 37.0801 |  |  | 3.13021 | 65.55016 |  |  | 2.27714 | 401.34738 | 11.6288 | 854.24364 |
| 3.0054 | 47.2347 |  |  | 3.24155 | 111.42681 |  |  | 2.49567 | 445.43304 | 11.8299 | 853.61091 |
| 3.23831 | 57.30213 |  |  | 3.31297 | 160.05584 |  |  | 2.66515 | 488.51674 | 12.0499 | 852.99507 |
| 3.34965 | 97.40622 |  |  | 3.40671 | 208.68488 |  |  | 2.84801 | 527.09168 | 12.2396 | 852.53852 |
| 3.42107 | 139.91637 |  |  | 3.57634 | 248.0744 |  |  | 3.03978 | 563.66274 | 12.4598 | 852.10775 |
| 3.51481 | 182.42652 |  |  | 3.73978 | 287.95386 |  |  | 3.2271 | 600.23379 | 12.6704 | 851.80851 |
| 3.68444 | 216.85974 |  |  | 3.87414 | 329.3645 |  |  | 3.41888 | 633.799 | 12.8771 | 851.63539 |
| 3.82821 | 253.59367 |  |  | 4.02817 | 375.059 |  |  | 3.65971 | 661.35253 | 13.0877 | 851.59615 |
| 4.00191 | 297.28336 |  |  | 4.21825 | 418.96856 |  |  | 3.95953 | 694.73681 | 13.306 | 851.71822 |
| 4.15266 | 333.48339 |  |  | 4.428 | 460.3792 |  |  | 4.26405 | 716.0673 | 13.5185 | 852.014 |
| 4.38207 | 375.30067 |  |  | 4.67707 | 491.43718 |  |  | 4.50963 | 735.19119 | 13.7234 | 852.48251 |
| 4.61148 | 409.62828 |  |  | 4.9032 | 526.77902 |  |  | 4.81742 | 758.72829 | 13.9226 | 853.12856 |
| 4.84088 | 442.39555 |  |  | 5.22765 | 562.12086 |  |  | 5.14159 | 780.05878 | 14.1351 | 854.04632 |
| 5.07029 | 478.59558 |  |  | 5.57176 | 597.4627 |  |  | 5.50177 | 799.55044 | 14.3533 | 855.26014 |
| 5.33575 | 509.49044 |  |  | 5.84049 | 626.73574 |  |  | 5.86523 | 814.26112 | 14.5676 | 856.74873 |
| 5.69952 | 544.13012 |  |  | 6.20099 | 653.50986 |  |  | 6.24506 | 831.54618 | 14.765 | 858.40695 |
| 6.02397 | 565.97497 |  |  | 6.52544 | 680.64097 |  |  | 6.66419 | 844.41803 | 14.9717 | 860.46824 |
| 6.30909 | 580.33015 |  |  | 6.90888 | 701.34629 |  |  | 7.04729 | 855.08327 |  |  |
| 6.63354 | 594.9974 |  |  | 7.26938 | 715.98281 |  |  | 7.46641 | 863.54192 |  |  |
| 7.01698 | 613.09742 |  |  | 7.61349 | 730.61933 |  |  | 7.86589 | 867.95512 |  |  |
| 7.37748 | 625.89226 |  |  | 8.01336 | 738.25507 |  |  | 8.28501 | 867.95512 |  |  |
| 7.72159 | 638.6871 |  |  | 8.45974 | 744.57684 |  |  | 8.72378 | 865.74852 |  |  |
| 8.12146 | 645.36204 |  |  | 8.90613 | 744.57684 |  |  | 9.04795 | 861.33532 |  |  |
| 8.56784 | 650.88836 |  |  | 9.35251 | 744.57684 |  |  | 9.36841 | 859.23699 |  |  |
| 9.01423 | 650.88836 |  |  | 9.79889 | 743.11797 |  |  | 9.81441 | 857.23309 |  |  |
| 9.46061 | 650.88836 |  |  | 10.24527 | 738.74136 |  |  | 10.2604 | 856.73212 |  |  |
| 9.90699 | 649.61306 |  |  | 10.69165 | 736.7962 |  |  | 10.70639 | 854.22725 |  |  |
| 10.35337 | 645.78715 |  |  | 11.13803 | 738.25507 |  |  | 11.15238 | 854.22725 |  |  |
| 10.79975 | 644.08674 |  |  | 11.58441 | 739.71394 |  |  | 11.59837 | 854.22725 |  |  |
| 11.24613 | 645.36204 |  |  | 12.03079 | 741.17281 |  |  | 12.04437 | 854.22725 |  |  |
| 11.69251 | 646.63735 |  |  | 12.47717 | 742.63168 |  |  | 12.49036 | 852.22336 |  |  |
| 12.13889 | 647.91265 |  |  |  |  |  |  | 12.93635 | 855.73017 |  |  |
| 12.58527 | 649.18796 |  |  |  |  |  |  | 13.38234 | 854.72823 |  |  |
|  |  |  |  |  |  |  |  | 13.82833 | 852.22336 |  |  |
|  |  |  |  |  |  |  |  | 14.27433 | 852.22336 |  |  |
|  |  |  |  |  |  |  |  | 14.72032 | 851.22141 |  |  |
