# Supplementary material for: Material composition and constitutive model development of red mud-based filler for highway tunnel invert filling applications: A comprehensive study
Source: PLoS One. 2025 Apr 16;20(4):e0321926. doi: 10.1371/journal.pone.0321926 (PMC12002488; doi:10.1371/journal.pone.0321926)
Supplement: S10 Table — Data of sp-ε1 curves. (DOCX) [file pone.0321926.s010.docx]

Table S10. The s_p_-ε_1_ curves of RMBF (Fig.15). Data of s_p_-ε_1_ curves.

(a) 7d.

| 30kPa | | 60kPa | | 90kPa | |
| --- | --- | --- | --- | --- | --- |
| ε_1_ | s_p_ | ε_1_ | s_p_ | ε_1_ | s_p_ |
| 0.2011 | 3.77509 | 0.2182 | 0.7807 | 0.1972 | 34.74324 |
| 0.4061 | 20.63149 | 0.425 | 1.36811 | 0.4155 | 74.20716 |
| 0.6147 | 37.82715 | 0.6584 | 5.49857 | 0.6071 | 84.82219 |
| 0.8159 | 52.12359 | 0.8461 | 12.65936 | 0.8291 | 115.87888 |
| 1.0265 | 76.98961 | 1.0625 | 29.78377 | 1.0208 | 124.44936 |
| 1.22 | 89.55203 | 1.2712 | 40.24083 | 1.2295 | 121.04729 |
| 1.4325 | 96.6171 | 1.4836 | 45.75143 | 1.4363 | 102.8331 |
| 1.6564 | 67.94908 | 1.6773 | 33.5585 | 1.662 | 95.52691 |
| 1.8823 | 51.07193 | 1.903 | 26.70595 | 1.8745 | 78.59464 |
| 2.1022 | 35.33743 | 2.0814 | 25.89569 | 2.0832 | 65.31047 |
| 2.2787 | 25.36071 | 2.2863 | 28.403 | 2.2995 | 38.97776 |
| 2.4969 | 23.29954 | 2.5044 | 26.61669 | 2.4779 | 26.79139 |
| 2.7019 | 21.02493 | 2.7189 | 27.75677 | 2.6999 | 19.73081 |
| 2.9125 | 21.89197 | 2.918 | 32.38149 | 2.9068 | 15.04107 |
| 3.125 | 21.24579 | 3.1268 | 32.97128 | 3.1192 | 12.58964 |
| 3.3355 | 20.7555 | 3.3506 | 34.70906 | 3.3317 | 10.6571 |
| 3.5366 | 23.20215 | 3.565 | 30.59062 | 3.5234 | 9.67839 |
| 3.7834 | 23.51485 | 3.7738 | 15.01314 | 3.732 | 8.46126 |
| 3.9522 | 27.66687 | 3.9882 | 10.84728 | 3.9711 | 8.14296 |
| 4.1628 | 30.11746 | 4.2007 | 9.03967 | 4.1475 | 7.68918 |
| 4.3791 | 32.7668 | 4.4094 | 7.92441 | 4.3658 | 6.82755 |
| 4.5689 | 34.85548 | 4.6238 | 6.84122 | 4.565 | 6.54952 |
| 4.7927 | 37.17295 | 4.8325 | 6.23016 | 4.787 | 6.27625 |
| 4.9976 | 19.23327 | 5.0108 | 5.84009 | 4.9805 | 6.17536 |
| 5.212 | 12.95996 | 5.2272 | 5.11258 | 5.1967 | 5.93628 |
| 5.4245 | 6.68853 | 5.4548 | 4.83025 | 5.4111 | 5.65934 |
| 5.62 | 5.96298 | 5.6616 | 4.70273 | 5.6085 | 5.48983 |
| 5.8135 | 5.12302 | 5.857 | 4.43398 | 5.8305 | 5.68564 |
| 6.0279 | 5.51502 | 6.0695 | 4.21618 | 6.0468 | 5.37731 |
| 6.2346 | 4.66449 | 6.2233 | 4.32884 | 6.2346 | 5.02869 |
| 6.4434 | 4.50812 | 6.4755 | 3.9268 | 6.4453 | 4.90422 |
| 6.6616 | 4.67219 | 6.6994 | 3.60446 | 6.6501 | 4.87532 |
| 6.857 | 4.56085 | 6.8968 | 3.67318 | 6.8626 | 4.87414 |
| 7.0753 | 4.42657 | 7.113 | 3.33161 | 7.0827 | 5.03354 |
| 7.2952 | 4.91543 | 7.3123 | 3.42054 | 7.2895 | 4.75929 |
| 7.4982 | 4.73282 | 7.5171 | 3.21795 | 7.504 | 4.68966 |
| 7.7146 | 4.5563 | 7.7203 | 3.19969 | 7.6994 | 4.48219 |
| 7.9139 | 4.51173 | 7.9516 | 3.12692 | 7.9251 | 4.45917 |
| 8.1035 | 4.38298 | 8.13 | 3.22775 | 8.1263 | 4.59218 |
| 8.3369 | 4.44967 | 8.3692 | 3.25893 | 8.3331 | 4.22551 |
| 8.54 | 4.40281 | 8.5759 | 2.74704 | 8.557 | 4.449 |
| 8.7449 | 4.4154 | 8.7714 | 2.81606 | 8.7505 | 4.37803 |
| 8.9441 | 4.06558 | 8.9858 | 2.68147 | 8.9345 | 4.11126 |
| 9.1755 | 4.04659 | 9.1925 | 2.62888 | 9.1622 | 4.45013 |
| 9.3634 | 3.87744 | 9.3804 | 2.97284 | 9.3822 | 4.65908 |
| 9.5645 | 3.76229 | 9.6061 | 2.58126 | 9.5645 | 4.16354 |
| 9.7732 | 3.58451 | 9.8186 | 2.51817 | 9.7884 | 4.31247 |
| 10.0009 | 3.56828 | 10.0311 | 2.47905 | 10.0009 | 4.0059 |
| 10.2096 | 3.44232 | 10.2323 | 2.74359 | 10.2 | 3.84254 |
| 10.424 | 3.46717 | 10.4296 | 2.72637 | 10.4069 | 3.7518 |
| 10.627 | 3.29006 | 10.6345 | 2.38337 | 10.6061 | 3.66464 |
| 10.8149 | 3.17593 | 10.8489 | 2.37975 | 10.8356 | 3.90788 |
| 11.0274 | 3.1406 | 11.0558 | 2.37506 | 11.0558 | 3.56283 |
| 11.2304 | 3.05523 | 11.2815 | 2.39154 | 11.2323 | 3.59465 |
|  |  | 11.4713 | 2.6041 | 11.4617 | 3.68801 |
|  |  | 11.6799 | 2.31036 | 11.6459 | 3.66214 |
|  |  | 11.8981 | 2.59193 | 11.8773 | 3.63571 |
|  |  | 12.0186 | 2.23418 | 12.0841 | 3.61461 |
|  |  |  |  | 12.272 | 3.45929 |
|  |  |  |  | 12.5034 | 3.48368 |
|  |  |  |  | 12.7084 | 3.52376 |
|  |  |  |  | 12.9209 | 3.43373 |
|  |  |  |  | 13.1257 | 3.49568 |
|  |  |  |  | 13.3364 | 3.3822 |
|  |  |  |  | 13.5355 | 3.18568 |
|  |  |  |  | 13.7366 | 3.21516 |
|  |  |  |  | 13.9473 | 3.23197 |
|  |  |  |  | 14.1636 | 3.15346 |

(b) 14d

| 30kPa | | 60kPa | | 90kPa | |
| --- | --- | --- | --- | --- | --- |
| ε_1_ | s_p_ | ε_1_ | s_p_ | ε_1_ | s_p_ |
| 0.2562 | 11.08904 | 0.1877 | 18.32508 | 0.203 | 8.06748 |
| 0.4364 | 23.48123 | 0.4041 | 29.08396 | 0.3946 | 25.83144 |
| 0.6394 | 48.12617 | 0.6128 | 57.46725 | 0.592 | 41.97884 |
| 0.8557 | 67.72545 | 0.8252 | 65.03679 | 0.8026 | 63.28591 |
| 1.0492 | 82.202 | 1.0189 | 72.05987 | 1.0416 | 77.89267 |
| 1.2522 | 48.4953 | 1.2446 | 36.39402 | 1.2105 | 44.75039 |
| 1.4761 | 35.19278 | 1.423 | 28.15066 | 1.4457 | 52.7038 |
| 1.6753 | 25.31924 | 1.6279 | 25.08635 | 1.6431 | 51.00487 |
| 1.8936 | 25.53591 | 1.846 | 34.61155 | 1.8309 | 49.05748 |
| 2.1022 | 24.46519 | 2.0605 | 29.36524 | 2.0776 | 63.95908 |
| 2.2824 | 25.29561 | 2.2596 | 33.16261 | 2.2843 | 58.78181 |
| 2.5045 | 27.5355 | 2.4684 | 35.39557 | 2.5026 | 49.38372 |
| 2.7113 | 30.60315 | 2.6922 | 37.4042 | 2.7075 | 38.6602 |
| 2.9333 | 34.30063 | 2.9066 | 38.33748 | 2.9029 | 26.99681 |
| 3.1419 | 36.78478 | 3.1154 | 32.47724 | 3.1097 | 19.65943 |
| 3.3583 | 31.2863 | 3.3298 | 14.33328 | 3.3185 | 15.01106 |
| 3.5537 | 11.60884 | 3.5423 | 7.95521 | 3.5366 | 12.0901 |
| 3.7453 | 7.49276 | 3.751 | 7.40819 | 3.7549 | 10.47621 |
| 3.9731 | 5.1866 | 3.9654 | 5.56576 | 3.9635 | 10.01851 |
| 4.1836 | 3.50828 | 4.1741 | 4.4799 | 4.1703 | 8.64699 |
| 4.4094 | 2.79693 | 4.3524 | 3.77366 | 4.3695 | 7.94842 |
| 4.5916 | 2.26119 | 4.5688 | 3.3659 | 4.5669 | 7.61491 |
| 4.7964 | 2.09577 | 4.7964 | 3.302 | 4.7831 | 6.89343 |
| 5.0147 | 2.20775 | 5.0032 | 3.04654 | 4.9823 | 6.91799 |
| 5.2291 | 2.00802 | 5.1986 | 2.81525 | 5.1892 | 6.36284 |
| 5.4207 | 1.85716 | 5.4111 | 3.1268 | 5.3922 | 6.12537 |
| 5.6446 | 1.88176 | 5.5649 | 1.86291 | 5.5857 | 5.77458 |
| 5.8362 | 1.81884 | 5.8171 | 2.9355 | 5.8191 | 5.88074 |
| 6.0696 | 1.92222 | 6.041 | 2.69942 | 6.0448 | 5.65796 |
| 6.2783 | 2.00934 | 6.2384 | 3.04158 | 6.2308 | 5.31217 |
| 6.4566 | 2.00114 | 6.4546 | 2.51929 | 6.4585 | 5.61078 |
| 6.6729 | 2.05696 | 6.6539 | 2.37164 | 6.6728 | 5.36785 |
| 6.8931 | 2.24847 | 6.8587 | 2.79704 | 6.8759 | 4.94621 |
| 7.1112 | 2.19316 | 7.0619 | 2.33511 | 7.0618 | 5.0199 |
| 7.2972 | 2.15762 | 7.2932 | 2.29266 | 7.2649 | 4.85778 |
| 7.5211 | 2.42356 | 7.4716 | 2.21261 | 7.4831 | 4.85293 |
| 7.7202 | 2.69915 | 7.7108 | 2.23856 | 7.6785 | 4.85369 |
| 7.9308 | 2.42018 | 7.9175 | 2.37077 | 7.9005 | 4.93691 |
| 8.1319 | 2.13017 | 8.113 | 2.45597 | 8.1281 | 5.1305 |
| 8.3141 | 2.05577 | 8.3274 | 2.57629 | 8.3331 | 4.9562 |
| 8.5437 | 2.04534 | 8.5341 | 2.72801 | 8.5398 | 4.63552 |
| 8.7694 | 2.17355 | 8.722 | 2.64367 | 8.7486 | 4.98886 |
| 8.9745 | 2.13223 | 8.9477 | 2.55293 | 8.9572 | 4.5569 |
| 9.185 | 1.99993 | 9.1602 | 2.78769 | 9.1717 | 4.50691 |
| 9.3786 | 2.00906 | 9.3727 | 2.87444 | 9.3709 | 4.09488 |
| 9.6006 | 2.15015 | 9.5739 | 2.6955 | 9.5587 | 4.43077 |
| 9.7941 | 1.9884 | 9.7712 | 2.79902 | 9.7902 | 4.32976 |
| 10.0236 | 1.92573 | 9.9761 | 2.93539 | 9.9914 | 4.43744 |
| 10.2191 | 1.90463 | 10.1905 | 2.81621 | 10.1925 | 4.55009 |
| 10.4391 | 2.02997 | 10.3974 | 2.95538 | 10.3992 | 4.01284 |
| 10.6288 | 1.98048 | 10.6231 | 3.14018 | 10.6042 | 4.10293 |
| 10.8489 | 2.08605 | 10.8129 | 3.01593 | 10.8413 | 4.02737 |
| 11.0633 | 2.12638 | 11.0215 | 3.11494 | 11.0349 | 4.0244 |
| 11.2493 | 2.11467 | 11.2397 | 3.48327 | 11.2512 | 4.22114 |
|  |  |  |  | 11.4599 | 4.14573 |
|  |  |  |  | 11.6459 | 4.09795 |
|  |  |  |  | 11.8772 | 4.09975 |
|  |  |  |  | 12.0878 | 3.84463 |
|  |  |  |  | 12.2908 | 3.41686 |
|  |  |  |  | 12.4977 | 3.53618 |
|  |  |  |  | 12.7045 | 3.21744 |
|  |  |  |  | 12.9151 | 3.24631 |
|  |  |  |  | 13.1257 | 3.49568 |

(c) 28d

| 30kPa | | 60kPa | | 90kPa | |
| --- | --- | --- | --- | --- | --- |
| ε_1_ | s_p_ | ε_1_ | s_p_ | ε_1_ | s_p_ |
| 0.1935 | 5.20749 | 0.2143 | 45.20727 | 0.201 | 5.25315 |
| 0.3946 | 14.5453 | 0.4268 | 72.11188 | 0.4021 | 28.44236 |
| 0.5939 | 26.12974 | 0.6298 | 106.10594 | 0.6355 | 52.24979 |
| 0.831 | 52.05092 | 0.8309 | 119.76627 | 0.8025 | 82.73177 |
| 1.0377 | 66.08002 | 1.0416 | 126.66929 | 1.0263 | 116.18461 |
| 1.2427 | 71.97682 | 1.275 | 92.78063 | 1.2237 | 124.64669 |
| 1.4096 | 52.2239 | 1.4817 | 80.71041 | 1.4476 | 119.51603 |
| 1.6677 | 39.25841 | 1.6847 | 70.03006 | 1.6544 | 92.66745 |
| 1.848 | 37.32169 | 1.8573 | 68.41904 | 1.8707 | 71.10437 |
| 2.0756 | 39.78218 | 2.1021 | 64.83094 | 2.0814 | 53.58814 |
| 2.2691 | 37.22062 | 2.2995 | 63.61151 | 2.2844 | 38.73316 |
| 2.4797 | 38.48996 | 2.5138 | 57.75712 | 2.5043 | 34.71556 |
| 2.7036 | 44.15244 | 2.7188 | 49.80301 | 2.6904 | 28.16007 |
| 2.9066 | 44.73828 | 2.9351 | 29.43527 | 2.9483 | 28.25989 |
| 3.0907 | 47.25783 | 3.1438 | 17.61458 | 3.1249 | 22.13301 |
| 3.3202 | 41.20275 | 3.3487 | 12.79535 | 3.3392 | 19.82026 |
| 3.5517 | 19.92224 | 3.5536 | 10.20017 | 3.5366 | 18.02998 |
| 3.7566 | 14.42112 | 3.7623 | 8.52367 | 3.7452 | 16.6377 |
| 3.954 | 12.07903 | 3.9786 | 7.15472 | 3.954 | 14.35987 |
| 4.1627 | 10.55026 | 4.1778 | 6.49975 | 4.1607 | 14.51011 |
| 4.3544 | 9.125 | 4.3959 | 6.00222 | 4.3752 | 12.64667 |
| 4.5801 | 8.26803 | 4.6028 | 5.60505 | 4.5839 | 12.27456 |
| 4.7889 | 7.71334 | 4.7982 | 5.14044 | 4.7471 | 11.14452 |
| 4.9728 | 6.79156 | 4.9898 | 5.0105 | 4.9975 | 10.13189 |
| 5.193 | 6.38835 | 5.2118 | 4.92168 | 5.2137 | 10.05099 |
| 5.3941 | 6.23166 | 5.4301 | 4.69278 | 5.4206 | 9.16332 |
| 5.618 | 5.86263 | 5.6388 | 4.82996 | 5.6104 | 7.89546 |
| 5.8342 | 5.57982 | 5.8437 | 4.59752 | 5.8285 | 8.25517 |
| 6.0372 | 5.71017 | 6.0523 | 4.82197 | 6.0258 | 7.5749 |
| 6.2422 | 5.21549 | 6.2611 | 4.18666 | 6.2574 | 7.15797 |
| 6.4357 | 4.80948 | 6.4717 | 4.0647 | 6.449 | 6.23386 |
| 6.6615 | 4.88929 | 6.6823 | 4.34401 | 6.6538 | 6.16492 |
| 6.8797 | 4.44339 | 6.8626 | 3.98709 | 6.8436 | 5.93322 |
| 7.0865 | 4.57063 | 7.1016 | 3.9163 | 7.0466 | 5.82295 |
| 7.2725 | 4.30718 | 7.3103 | 3.86759 | 7.2819 | 5.57991 |
| 7.4773 | 4.29152 | 7.4906 | 3.81523 | 7.4792 | 5.7817 |
| 7.6917 | 4.20336 | 7.7126 | 3.76003 | 7.705 | 5.69205 |
| 7.908 | 4.3414 | 7.9402 | 3.7171 | 7.9042 | 5.25225 |
| 8.1167 | 4.41038 | 8.1205 | 3.7134 | 8.1224 | 5.56726 |
| 8.3311 | 3.71395 | 8.3292 | 3.74281 | 8.3348 | 5.52614 |
| 8.5285 | 3.82299 | 8.5607 | 3.7472 | 8.5209 | 5.0831 |
| 8.7486 | 3.64699 | 8.7656 | 4.05302 | 8.7296 | 5.28632 |
| 8.9382 | 3.58295 | 8.9648 | 3.47742 | 8.9326 | 4.95964 |
| 9.1565 | 4.05651 | 9.1811 | 3.60924 | 9.1602 | 5.18621 |
| 9.3709 | 3.52059 | 9.3954 | 3.56646 | 9.3709 | 4.98618 |
| 9.5739 | 3.44958 | 9.5985 | 3.45256 | 9.5587 | 4.55462 |
| 9.7731 | 3.40884 | 9.8072 | 3.52723 | 9.7712 | 4.60726 |
| 9.9932 | 3.75672 | 10.0026 | 3.36806 | 9.9875 | 4.41142 |
| 10.2018 | 3.72655 | 10.2246 | 3.4121 | 10.2018 | 4.33125 |
| 10.3974 | 3.27075 | 10.4238 | 3.46262 | 10.4106 | 4.20899 |
| 10.6175 | 3.26939 | 10.6307 | 3.65638 | 10.6099 | 4.24723 |
| 10.8337 | 3.25657 | 10.8242 | 3.44189 | 10.811 | 4.17266 |
| 11.0159 | 3.29089 | 11.0651 | 3.47315 | 11.031 | 3.95241 |
| 11.2455 | 3.56455 | 11.2682 | 3.59929 | 11.2207 | 3.7786 |
| 11.4485 | 3.15903 | 11.4768 | 3.60638 | 11.4409 | 4.03659 |
| 11.6552 | 3.53515 | 11.6893 | 3.82481 | 11.6515 | 4.04537 |
| 11.8563 | 3.04201 | 11.8696 | 3.72045 | 11.8582 | 3.82577 |
|  |  | 12.0992 | 3.84506 | 12.0688 | 3.86152 |
|  |  |  |  | 12.2871 | 3.93309 |
|  |  |  |  | 12.4996 | 3.56226 |
|  |  |  |  | 12.7045 | 3.86043 |
|  |  |  |  | 12.9037 | 3.49517 |
|  |  |  |  | 13.1162 | 3.33376 |
|  |  |  |  | 13.3344 | 3.61078 |
|  |  |  |  | 13.5487 | 3.57023 |
|  |  |  |  | 13.7461 | 3.66379 |
|  |  |  |  | 13.9528 | 3.26716 |
