# Supplementary material for: Material composition and constitutive model development of red mud-based filler for highway tunnel invert filling applications: A comprehensive study
Source: PLoS One. 2025 Apr 16;20(4):e0321926. doi: 10.1371/journal.pone.0321926 (PMC12002488; doi:10.1371/journal.pone.0321926)
Supplement: S11 Table — Data of strengthening stress-strain curve. (DOCX) [file pone.0321926.s011.docx]

Table S11. Strengthening stress-strain curve of RMBF (Fig.16). Data of strengthening stress-strain curve.

(a) 7d

| 30kPa | | 60kPa | | 90kPa | |
| --- | --- | --- | --- | --- | --- |
| ε_1_ | (σ_1_-σ_3_)_s_ | ε_1_ | (σ_1_-σ_3_)_s_ | ε_1_ | (σ_1_-σ_3_)_s_ |
| 0.2011 | 11.54124 | 0.2182 | 12.03991 | 0.1972 | 15.40435 |
| 0.4061 | 20.45583 | 0.425 | 25.52123 | 0.4155 | 32.79836 |
| 0.6147 | 30.49997 | 0.6584 | 44.56891 | 0.6071 | 45.7274 |
| 0.8159 | 40.19284 | 0.8461 | 52.81995 | 0.8291 | 58.63775 |
| 1.0265 | 45.05547 | 1.0625 | 61.38305 | 1.0208 | 71.29162 |
| 1.22 | 60.94709 | 1.2712 | 75.77729 | 1.2295 | 84.94279 |
| 1.4325 | 67.5853 | 1.4836 | 82.68197 | 1.4363 | 90.35756 |
| 1.6564 | 75.83376 | 1.6773 | 94.09521 | 1.662 | 107.75156 |
| 1.8823 | 84.38669 | 1.903 | 105.62638 | 1.8745 | 125.14557 |
| 2.1022 | 90.5444 | 2.0814 | 100.23921 | 2.0832 | 132.53957 |
| 2.2787 | 100.45877 | 2.2863 | 115.61937 | 2.2995 | 149.93357 |
| 2.4969 | 100.60659 | 2.5044 | 126.42352 | 2.4779 | 147.32757 |
| 2.7019 | 112.34857 | 2.7189 | 137.79492 | 2.6999 | 154.72158 |
| 2.9125 | 113.16258 | 2.918 | 144.27845 | 2.9068 | 162.11558 |
| 3.125 | 106.64016 | 3.1268 | 155.68435 | 3.1192 | 169.50958 |
| 3.3355 | 99.6138 | 3.3506 | 167.66343 | 3.3317 | 176.90359 |
| 3.5366 | 96.30954 | 3.565 | 172.28477 | 3.5234 | 180.04157 |
| 3.7834 | 97.37002 | 3.7738 | 179.50728 | 3.732 | 178.95925 |
| 3.9522 | 99.44506 | 3.9882 | 182.75256 | 3.9711 | 185.98226 |
| 4.1628 | 109.00327 | 4.2007 | 198.40786 | 4.1475 | 193.74151 |
| 4.3791 | 113.54002 | 4.4094 | 201.03736 | 4.3658 | 208.29202 |
| 4.5689 | 114.79736 | 4.6238 | 204.28265 | 4.565 | 214.81644 |
| 4.7927 | 119.00273 | 4.8325 | 207.52793 | 4.787 | 221.52357 |
| 4.9976 | 121.11264 | 5.0108 | 210.77321 | 4.9805 | 224.86947 |
| 5.212 | 135.07086 | 5.2272 | 219.36008 | 5.1967 | 230.82853 |
| 5.4245 | 174.88829 | 5.4548 | 226.95155 | 5.4111 | 237.64008 |
| 5.62 | 185.8815 | 5.6616 | 231.02318 | 5.6085 | 242.22242 |
| 5.8135 | 201.14859 | 5.857 | 238.79186 | 5.8305 | 238.85112 |
| 6.0279 | 194.42617 | 6.0695 | 245.74189 | 6.0468 | 246.25814 |
| 6.2346 | 211.9153 | 6.2233 | 243.07816 | 6.2346 | 255.11015 |
| 6.4434 | 216.01282 | 6.4755 | 256.08179 | 6.4453 | 258.72808 |
| 6.6616 | 212.59832 | 6.6994 | 268.0075 | 6.6501 | 259.78299 |
| 6.857 | 215.51503 | 6.8968 | 266.06959 | 6.8626 | 260.03 |
| 7.0753 | 219.11224 | 7.113 | 280.00298 | 7.0827 | 256.02994 |
| 7.2952 | 208.24769 | 7.3123 | 276.88015 | 7.2895 | 263.40065 |
| 7.4982 | 212.51309 | 7.5171 | 286.01326 | 7.504 | 265.41649 |
| 7.7146 | 216.89476 | 7.7203 | 287.35755 | 7.6994 | 271.53445 |
| 7.9139 | 218.24303 | 7.9516 | 291.27469 | 7.9251 | 272.2773 |
| 8.1035 | 221.69612 | 8.13 | 287.1283 | 8.1263 | 268.34538 |
| 8.3369 | 220.36288 | 8.3692 | 286.32438 | 8.3331 | 279.79927 |
| 8.54 | 221.82967 | 8.5759 | 312.38818 | 8.557 | 272.75722 |
| 8.7449 | 221.81688 | 8.7714 | 309.01395 | 8.7505 | 275.04678 |
| 8.9441 | 231.47498 | 8.9858 | 317.19383 | 8.9345 | 283.93876 |
| 9.1755 | 232.38404 | 9.1925 | 320.83616 | 9.1622 | 273.07434 |
| 9.3634 | 237.70412 | 9.3804 | 302.10116 | 9.3822 | 267.06626 |
| 9.5645 | 241.64532 | 9.6061 | 324.68551 | 9.5645 | 282.70176 |
| 9.7732 | 247.91317 | 9.8186 | 329.14865 | 9.7884 | 278.03745 |
| 10.0009 | 248.84941 | 10.0311 | 332.1196 | 10.0009 | 288.76675 |
| 10.2096 | 253.69822 | 10.2323 | 316.00973 | 10.2 | 295.13962 |
| 10.424 | 253.11821 | 10.4296 | 317.26851 | 10.4069 | 299.02168 |
| 10.627 | 260.14634 | 10.6345 | 339.57561 | 10.6061 | 302.89755 |
| 10.8149 | 265.04834 | 10.8489 | 340.03651 | 10.8356 | 293.70989 |
| 11.0274 | 266.81795 | 11.0558 | 340.51494 | 11.0558 | 307.99752 |
| 11.2304 | 270.76893 | 11.2815 | 339.43577 | 11.2323 | 306.93993 |
|  |  | 11.4713 | 325.32093 | 11.4617 | 303.40921 |
|  |  | 11.6799 | 345.37683 | 11.6459 | 304.76171 |
|  |  | 11.8981 | 326.03005 | 11.8773 | 306.18416 |
|  |  | 12.0186 | 351.1181 | 12.0841 | 307.31145 |
|  |  |  |  | 12.272 | 314.3011 |
|  |  |  |  | 12.5034 | 313.32329 |
|  |  |  |  | 12.7084 | 311.5595 |
|  |  |  |  | 12.9209 | 315.54173 |
|  |  |  |  | 13.1257 | 312.55269 |
|  |  |  |  | 13.3364 | 317.43768 |
|  |  |  |  | 13.5355 | 327.14813 |
|  |  |  |  | 13.7366 | 325.68744 |
|  |  |  |  | 13.9473 | 324.85958 |
|  |  |  |  | 14.1636 | 328.87608 |

(b) 14d

| 30kPa | | 60kPa | | 90kPa | |
| --- | --- | --- | --- | --- | --- |
| ε_1_ | (σ_1_-σ_3_)_s_ | ε_1_ | (σ_1_-σ_3_)_s_ | ε_1_ | (σ_1_-σ_3_)_s_ |
| 0.2562 | 40.17301 | 0.1877 | 16.49551 | 0.203 | 12.02356 |
| 0.4364 | 65.20082 | 0.4041 | 32.22189 | 0.3946 | 26.5968 |
| 0.6394 | 90.98046 | 0.6128 | 51.3885 | 0.592 | 37.32131 |
| 0.8557 | 104.32935 | 0.8252 | 70.89934 | 0.8026 | 47.37979 |
| 1.0492 | 120.68847 | 1.0189 | 77.39804 | 1.0416 | 59.42038 |
| 1.2522 | 135.62697 | 1.2446 | 94.05532 | 1.2105 | 72.8377 |
| 1.4761 | 145.1298 | 1.423 | 104.53234 | 1.4457 | 84.16676 |
| 1.6753 | 157.01762 | 1.6279 | 112.63338 | 1.6431 | 98.50227 |
| 1.8936 | 160.44315 | 1.846 | 127.24052 | 1.8309 | 102.63481 |
| 2.1022 | 174.88859 | 2.0605 | 142.96691 | 2.0776 | 117.20805 |
| 2.2824 | 189.82709 | 2.2596 | 158.69329 | 2.2843 | 121.7813 |
| 2.5045 | 204.76559 | 2.4684 | 154.41968 | 2.5026 | 126.35454 |
| 2.7113 | 209.70408 | 2.6922 | 170.14607 | 2.7075 | 130.92779 |
| 2.9333 | 204.64258 | 2.9066 | 175.87245 | 2.9029 | 141.50103 |
| 3.1419 | 216.07986 | 3.1154 | 181.59884 | 3.1097 | 150.07427 |
| 3.3583 | 213.50965 | 3.3298 | 194.45404 | 3.3185 | 154.99368 |
| 3.5537 | 220.93944 | 3.5423 | 206.30682 | 3.5366 | 159.15561 |
| 3.7453 | 218.36923 | 3.751 | 214.3089 | 3.7549 | 175.40694 |
| 3.9731 | 225.79903 | 3.9654 | 211.92284 | 3.9635 | 161.20518 |
| 4.1836 | 233.22882 | 4.1741 | 216.09608 | 4.1703 | 175.64154 |
| 4.4094 | 240.65861 | 4.3524 | 237.18853 | 4.3695 | 185.04945 |
| 4.5916 | 268.0884 | 4.5688 | 253.05407 | 4.5669 | 190.68664 |
| 4.7964 | 279.94427 | 4.7964 | 257.18755 | 4.7831 | 202.02974 |
| 5.0147 | 274.07146 | 5.0032 | 269.10257 | 4.9823 | 202.94079 |
| 5.2291 | 288.55649 | 5.1986 | 281.07066 | 5.1892 | 212.78737 |
| 5.4207 | 301.01849 | 5.4111 | 267.70262 | 5.3922 | 217.87843 |
| 5.6446 | 300.05895 | 5.5649 | 281.64645 | 5.5857 | 225.24358 |
| 5.8362 | 306.01524 | 5.8171 | 277.8893 | 5.8191 | 224.05509 |
| 6.0696 | 298.57073 | 6.041 | 290.55133 | 6.0448 | 229.12574 |
| 6.2783 | 292.77474 | 6.2384 | 274.29921 | 6.2308 | 236.97407 |
| 6.4566 | 293.99866 | 6.4546 | 302.043 | 6.4585 | 231.09933 |
| 6.6729 | 290.71934 | 6.6539 | 311.8909 | 6.6728 | 236.69423 |
| 6.8931 | 278.77826 | 6.8587 | 287.73846 | 6.8759 | 246.93827 |
| 7.1112 | 282.99054 | 7.0619 | 315.49923 | 7.0618 | 245.40922 |
| 7.2972 | 285.93347 | 7.2932 | 319.08263 | 7.2649 | 249.75787 |
| 7.5211 | 270.49947 | 7.4716 | 325.34234 | 7.4831 | 250.15922 |
| 7.7202 | 256.9172 | 7.7108 | 324.18442 | 7.6785 | 250.36432 |
| 7.9308 | 271.98679 | 7.9175 | 315.64583 | 7.9005 | 248.47839 |
| 8.1319 | 290.58083 | 8.113 | 310.72145 | 8.1281 | 243.96182 |
| 8.3141 | 296.39893 | 8.3274 | 304.03148 | 8.3331 | 248.39997 |
| 8.5437 | 297.89691 | 8.5341 | 296.07518 | 8.5398 | 257.03014 |
| 8.7694 | 289.6512 | 8.722 | 301.33603 | 8.7486 | 247.92703 |
| 8.9745 | 293.02498 | 8.9477 | 307.34697 | 8.9572 | 259.57418 |
| 9.185 | 303.12997 | 9.1602 | 294.74809 | 9.1717 | 261.16436 |
| 9.3786 | 302.91245 | 9.3727 | 290.87168 | 9.3709 | 274.12596 |
| 9.6006 | 293.26641 | 9.5739 | 300.94707 | 9.5587 | 263.64181 |
| 9.7941 | 305.31642 | 9.7712 | 295.86327 | 9.7902 | 266.81804 |
| 10.0236 | 310.59096 | 9.9761 | 289.42573 | 9.9914 | 263.64278 |
| 10.2191 | 312.53008 | 10.1905 | 296.00688 | 10.1925 | 260.41988 |
| 10.4391 | 302.89183 | 10.3974 | 289.40951 | 10.3992 | 277.34787 |
| 10.6288 | 306.72864 | 10.6231 | 281.20912 | 10.6042 | 274.3022 |
| 10.8489 | 298.88088 | 10.8129 | 287.29195 | 10.8413 | 276.84807 |
| 11.0633 | 295.9801 | 11.0215 | 283.03289 | 11.0349 | 276.90974 |
| 11.2493 | 296.70726 | 11.2397 | 267.95787 | 11.2512 | 270.30609 |
|  |  |  |  | 11.4599 | 272.65358 |
|  |  |  |  | 11.6459 | 274.12596 |
|  |  |  |  | 11.8772 | 273.89959 |
|  |  |  |  | 12.0878 | 282.66296 |
|  |  |  |  | 12.2908 | 299.63784 |
|  |  |  |  | 12.4977 | 294.33392 |
|  |  |  |  | 12.7045 | 308.35542 |
|  |  |  |  | 12.9151 | 306.77849 |
|  |  |  |  | 13.1257 | 309.78723 |

(c) 28d

| 30kPa | | 60kPa | | 90kPa | |
| --- | --- | --- | --- | --- | --- |
| ε_1_ | (σ_1_-σ_3_)_s_ | ε_1_ | (σ_1_-σ_3_)_s_ | ε_1_ | (σ_1_-σ_3_)_s_ |
| 0.1935 | 8.58331 | 0.2143 | 19.77508 | 0.201 | 12.05088 |
| 0.3946 | 24.50529 | 0.4268 | 33.29934 | 0.4021 | 20.27442 |
| 0.5939 | 25.58643 | 0.6298 | 44.89152 | 0.6355 | 31.6826 |
| 0.831 | 43.66612 | 0.8309 | 57.33501 | 0.8025 | 40.75255 |
| 1.0377 | 50.85187 | 1.0416 | 79.91439 | 1.0263 | 51.18968 |
| 1.2427 | 57.11652 | 1.275 | 86.8165 | 1.2237 | 63.3294 |
| 1.4096 | 64.71119 | 1.4817 | 101.76665 | 1.4476 | 76.87081 |
| 1.6677 | 70.63795 | 1.6847 | 106.96347 | 1.6544 | 83.43072 |
| 1.848 | 82.71947 | 1.8573 | 119.62499 | 1.8707 | 91.33874 |
| 2.0756 | 90.40705 | 2.1021 | 123.61055 | 2.0814 | 100.85704 |
| 2.2691 | 94.62942 | 2.2995 | 136.11909 | 2.2844 | 103.16102 |
| 2.4797 | 96.12207 | 2.5138 | 145.52292 | 2.5043 | 110.18158 |
| 2.7036 | 112.04404 | 2.7188 | 159.04717 | 2.6904 | 120.88052 |
| 2.9066 | 117.96601 | 2.9351 | 162.57143 | 2.9483 | 124.41058 |
| 3.0907 | 123.88799 | 3.1438 | 166.09568 | 3.1249 | 130.93074 |
| 3.3202 | 129.80996 | 3.3487 | 179.61994 | 3.3392 | 136.44636 |
| 3.5517 | 135.73193 | 3.5536 | 183.1442 | 3.5366 | 141.17306 |
| 3.7566 | 137.14683 | 3.7623 | 184.66845 | 3.7452 | 151.38766 |
| 3.954 | 138.63162 | 3.9786 | 189.21326 | 3.954 | 163.09554 |
| 4.1627 | 138.4584 | 4.1778 | 190.60492 | 4.1607 | 164.53809 |
| 4.3544 | 139.42355 | 4.3959 | 188.94846 | 4.3752 | 176.20992 |
| 4.5801 | 147.84052 | 4.6028 | 197.2658 | 4.5839 | 177.23983 |
| 4.7889 | 154.17153 | 4.7982 | 207.47868 | 4.7471 | 179.01469 |
| 4.9728 | 165.18976 | 4.9898 | 211.44679 | 4.9975 | 181.6714 |
| 5.193 | 171.25427 | 5.2118 | 214.65965 | 5.2137 | 190.7674 |
| 5.3941 | 174.11719 | 5.4301 | 220.97136 | 5.4206 | 195.08497 |
| 5.618 | 180.20881 | 5.6388 | 218.74184 | 5.6104 | 195.0761 |
| 5.8342 | 185.2914 | 5.8437 | 225.02352 | 5.8285 | 191.82349 |
| 6.0372 | 183.61019 | 6.0523 | 220.44225 | 6.0258 | 201.10367 |
| 6.2422 | 192.5195 | 6.2611 | 237.26167 | 6.2574 | 207.75871 |
| 6.4357 | 200.81588 | 6.4717 | 241.42078 | 6.449 | 223.29319 |
| 6.6615 | 199.50177 | 6.6823 | 234.07702 | 6.6538 | 225.15548 |
| 6.8797 | 209.5612 | 6.8626 | 244.77741 | 6.8436 | 230.01016 |
| 7.0865 | 206.86003 | 7.1016 | 247.53209 | 7.0466 | 232.64081 |
| 7.2725 | 213.28937 | 7.3103 | 249.53471 | 7.2819 | 238.11351 |
| 7.4773 | 213.87643 | 7.4906 | 251.60896 | 7.4792 | 234.23908 |
| 7.6917 | 216.30103 | 7.7126 | 253.88064 | 7.705 | 236.38444 |
| 7.908 | 213.01374 | 7.9402 | 255.76261 | 7.9042 | 246.31918 |
| 8.1167 | 211.50368 | 8.1205 | 256.20664 | 8.1224 | 239.4606 |
| 8.3311 | 230.65539 | 8.3292 | 255.54637 | 8.3348 | 240.52233 |
| 8.5285 | 227.49252 | 8.5607 | 255.76225 | 8.5209 | 250.91814 |
| 8.7486 | 233.0819 | 8.7656 | 246.21685 | 8.7296 | 246.1715 |
| 8.9382 | 235.29232 | 8.9648 | 266.10441 | 8.9326 | 254.25345 |
| 9.1565 | 221.27309 | 9.1811 | 261.48795 | 9.1602 | 248.73184 |
| 9.3709 | 237.65879 | 9.3954 | 263.31687 | 9.3709 | 253.74453 |
| 9.5739 | 240.21848 | 9.5985 | 267.8596 | 9.5587 | 265.54926 |
| 9.7731 | 241.76571 | 9.8072 | 265.22635 | 9.7712 | 264.07831 |
| 9.9932 | 230.41368 | 10.0026 | 271.60917 | 9.9875 | 269.91615 |
| 10.2018 | 231.44509 | 10.2246 | 270.04172 | 10.2018 | 272.43118 |
| 10.3974 | 247.14083 | 10.4238 | 268.21727 | 10.4106 | 276.37693 |
| 10.6175 | 247.29455 | 10.6307 | 261.15482 | 10.6099 | 275.13752 |
| 10.8337 | 247.88099 | 10.8242 | 269.29617 | 10.811 | 277.58385 |
| 11.0159 | 246.6727 | 11.0651 | 268.23534 | 11.031 | 285.20178 |
| 11.2455 | 237.13243 | 11.2682 | 263.62555 | 11.2207 | 291.66975 |
| 11.4485 | 252.02276 | 11.4768 | 263.5174 | 11.4409 | 282.16718 |
| 11.6552 | 238.39023 | 11.6893 | 256.05878 | 11.6515 | 281.82699 |
| 11.8563 | 257.18601 | 11.8696 | 259.80982 | 11.8582 | 289.76325 |
|  |  | 12.0992 | 255.85661 | 12.0688 | 288.37591 |
|  |  |  |  | 12.2871 | 285.6954 |
|  |  |  |  | 12.4996 | 300.15498 |
|  |  |  |  | 12.7045 | 288.29762 |
|  |  |  |  | 12.9037 | 302.96577 |
|  |  |  |  | 13.1162 | 310.20871 |
|  |  |  |  | 13.3344 | 298.09412 |
|  |  |  |  | 13.5487 | 299.84049 |
|  |  |  |  | 13.7461 | 296.0809 |
|  |  |  |  | 13.9528 | 313.69718 |
