# Supplementary material for: Material composition and constitutive model development of red mud-based filler for highway tunnel invert filling applications: A comprehensive study
Source: PLoS One. 2025 Apr 16;20(4):e0321926. doi: 10.1371/journal.pone.0321926 (PMC12002488; doi:10.1371/journal.pone.0321926)
Supplement: S12 Table — Data of “ab line” of RMBF considering Sp. (DOCX) [file pone.0321926.s012.docx]

Table S12. The "ab line" of RMBF considering Sp (Fig.17). Data of "ab line" of RMBF considering Sp.

(a) 7d

| 30kPa | | 60kPa | | 90kPa | |
| --- | --- | --- | --- | --- | --- |
| ε_1_ | ε_1_ /(σ_1_-σ_3_)_s_ | ε_1_ | ε_1_ /(σ_1_-σ_3_)_s_ | ε_1_ | ε_1_ /(σ_1_-σ_3_)_s_ |
| 5.4245 | 0.03102 | 5.2272 | 0.02383 | 3.732 | 0.02085 |
| 5.62 | 0.03023 | 5.4548 | 0.02404 | 3.9711 | 0.02135 |
| 5.8135 | 0.0289 | 5.6616 | 0.02451 | 4.1475 | 0.02141 |
| 6.0279 | 0.031 | 5.857 | 0.02453 | 4.3658 | 0.02096 |
| 6.2346 | 0.02942 | 6.0695 | 0.0247 | 4.565 | 0.02125 |
| 6.4434 | 0.02983 | 6.2233 | 0.0256 | 4.787 | 0.02161 |
| 6.6616 | 0.03133 | 6.4755 | 0.02529 | 4.9805 | 0.02215 |
| 6.857 | 0.03182 | 6.6994 | 0.025 | 5.1967 | 0.02251 |
| 7.0753 | 0.03229 | 6.8968 | 0.02592 | 5.4111 | 0.02277 |
| 7.2952 | 0.03503 | 7.113 | 0.0254 | 5.6085 | 0.02315 |
| 7.4982 | 0.03528 | 7.3123 | 0.02641 | 5.8305 | 0.02441 |
| 7.7146 | 0.03557 | 7.5171 | 0.02628 | 6.0468 | 0.02455 |
| 7.9139 | 0.03626 | 7.7203 | 0.02687 | 6.2346 | 0.02444 |
| 8.1035 | 0.03655 | 7.9516 | 0.0273 | 6.4453 | 0.02491 |
| 8.3369 | 0.03783 | 8.13 | 0.02831 | 6.6501 | 0.0256 |
| 8.54 | 0.0385 | 8.3692 | 0.02923 | 6.8626 | 0.02639 |
| 8.7449 | 0.03942 | 8.5759 | 0.02745 | 7.0827 | 0.02766 |
| 8.9441 | 0.03864 | 8.7714 | 0.02839 | 7.2895 | 0.02767 |
| 9.1755 | 0.03948 | 8.9858 | 0.02833 | 7.504 | 0.02827 |
| 9.3634 | 0.03939 | 9.1925 | 0.02865 | 7.6994 | 0.02836 |
| 9.5645 | 0.03958 | 9.3804 | 0.03105 | 7.9251 | 0.02911 |
| 9.7732 | 0.03942 | 9.6061 | 0.02959 | 8.1263 | 0.03028 |
| 10.0009 | 0.04019 | 9.8186 | 0.02983 | 8.3331 | 0.02978 |
| 10.2096 | 0.04024 | 10.0311 | 0.0302 | 8.557 | 0.03137 |
| 10.424 | 0.04118 | 10.2323 | 0.03238 | 8.7505 | 0.03181 |
| 10.627 | 0.04085 | 10.4296 | 0.03287 | 8.9345 | 0.03147 |
| 10.8149 | 0.0408 | 10.6345 | 0.03132 | 9.1622 | 0.03355 |
| 11.0274 | 0.04133 | 10.8489 | 0.03191 | 9.3822 | 0.03513 |
| 11.2304 | 0.04148 | 11.0558 | 0.03247 | 9.5645 | 0.03383 |
|  |  | 11.2815 | 0.03324 | 9.7884 | 0.03521 |
|  |  | 11.4713 | 0.03526 | 10.0009 | 0.03463 |
|  |  | 11.6799 | 0.03382 | 10.2 | 0.03456 |
|  |  | 11.8981 | 0.03649 | 10.4069 | 0.0348 |
|  |  | 12.0186 | 0.03423 | 10.6061 | 0.03502 |
|  |  |  |  | 10.8356 | 0.03689 |
|  |  |  |  | 11.0558 | 0.0359 |
|  |  |  |  | 11.2323 | 0.03659 |
|  |  |  |  | 11.4617 | 0.03778 |
|  |  |  |  | 11.6459 | 0.03821 |
|  |  |  |  | 11.8773 | 0.03879 |
|  |  |  |  | 12.0841 | 0.03932 |
|  |  |  |  | 12.272 | 0.03905 |
|  |  |  |  | 12.5034 | 0.03991 |
|  |  |  |  | 12.7084 | 0.04079 |
|  |  |  |  | 12.9209 | 0.04095 |
|  |  |  |  | 13.1257 | 0.042 |
|  |  |  |  | 13.3364 | 0.04201 |
|  |  |  |  | 13.5355 | 0.04137 |
|  |  |  |  | 13.7366 | 0.04218 |
|  |  |  |  | 13.9473 | 0.04293 |
|  |  |  |  | 14.1636 | 0.04307 |

(b) 14d

| 30kPa | | 60kPa | | 90kPa | |
| --- | --- | --- | --- | --- | --- |
| ε_1_ | ε_1_ /(σ_1_-σ_3_)_s_ | ε_1_ | ε_1_ /(σ_1_-σ_3_)_s_ | ε_1_ | ε_1_ /(σ_1_-σ_3_)_s_ |
| 4.3544 | 0.03123 | 6.0258 | 0.02996 | 3.9786 | 0.02351 |
| 4.5801 | 0.03098 | 6.2574 | 0.03012 | 4.1778 | 0.02326 |
| 4.7889 | 0.03106 | 6.449 | 0.02888 | 4.3959 | 0.02327 |
| 4.9728 | 0.0301 | 6.6538 | 0.02955 | 4.6028 | 0.02333 |
| 5.193 | 0.03032 | 6.8436 | 0.02975 | 4.7982 | 0.02313 |
| 5.3941 | 0.03098 | 7.0466 | 0.03029 | 4.9898 | 0.0236 |
| 5.618 | 0.03117 | 7.2819 | 0.03058 | 5.2118 | 0.02428 |
| 5.8342 | 0.03149 | 7.4792 | 0.03193 | 5.4301 | 0.02457 |
| 6.0372 | 0.03288 | 7.705 | 0.0326 | 5.6388 | 0.02578 |
| 6.2422 | 0.03242 | 7.9042 | 0.03209 | 5.8437 | 0.02597 |
| 6.4357 | 0.03205 | 8.1224 | 0.03392 | 6.0523 | 0.02746 |
| 6.6615 | 0.03339 | 8.3348 | 0.03465 | 6.2611 | 0.02639 |
| 6.8797 | 0.03283 | 8.5209 | 0.03396 | 6.4717 | 0.02681 |
| 7.0865 | 0.03426 | 8.7296 | 0.03546 | 6.6823 | 0.02855 |
| 7.2725 | 0.0341 | 8.9326 | 0.03513 | 6.8626 | 0.02804 |
| 7.4773 | 0.03496 | 9.1602 | 0.03683 | 7.1016 | 0.02869 |
| 7.6917 | 0.03556 | 9.3709 | 0.03693 | 7.3103 | 0.0293 |
| 7.908 | 0.03712 | 9.5587 | 0.036 | 7.4906 | 0.02977 |
| 8.1167 | 0.03838 | 9.7712 | 0.037 | 7.7126 | 0.03038 |
| 8.3311 | 0.03612 | 9.9875 | 0.037 | 7.9402 | 0.03105 |
| 8.5285 | 0.03749 | 10.2018 | 0.03745 | 8.1205 | 0.0317 |
| 8.7486 | 0.03753 | 10.4106 | 0.03767 | 8.3292 | 0.03259 |
| 8.9382 | 0.03799 | 10.6099 | 0.03856 | 8.5607 | 0.03347 |
| 9.1565 | 0.04138 | 10.811 | 0.03895 | 8.7656 | 0.0356 |
| 9.3709 | 0.03943 | 11.031 | 0.03868 | 8.9648 | 0.03369 |
| 9.5739 | 0.03985 | 11.2207 | 0.03847 | 9.1811 | 0.03511 |
| 9.7731 | 0.04042 | 11.4409 | 0.04055 | 9.3954 | 0.03568 |
| 9.9932 | 0.04337 | 11.6515 | 0.04134 | 9.5985 | 0.03583 |
| 10.2018 | 0.04408 | 11.8582 | 0.04092 | 9.8072 | 0.03698 |
| 10.3974 | 0.04207 | 12.0688 | 0.04185 | 10.0026 | 0.03683 |
| 10.6175 | 0.04293 | 12.2871 | 0.04301 | 10.2246 | 0.03786 |
| 10.8337 | 0.04371 | 12.4996 | 0.04164 | 10.4238 | 0.03886 |
| 11.0159 | 0.04466 | 12.7045 | 0.04407 | 10.6307 | 0.04071 |
| 11.2455 | 0.04742 | 12.9037 | 0.04259 | 10.8242 | 0.04019 |
| 11.4485 | 0.04543 | 13.1162 | 0.04228 | 11.0651 | 0.04125 |
| 11.6552 | 0.04889 | 13.3344 | 0.04473 | 11.2682 | 0.04274 |
| 11.8563 | 0.0461 | 13.5487 | 0.04519 | 11.4768 | 0.04355 |
|  |  | 13.7461 | 0.04643 |  |  |
|  |  | 13.9528 | 0.04448 |  |  |

(c) 28d

| 30kPa | | 60kPa | | 90kPa | |
| --- | --- | --- | --- | --- | --- |
| ε_1_ | ε_1_ /(σ_1_-σ_3_)_s_ | ε_1_ | ε_1_ /(σ_1_-σ_3_)_s_ | ε_1_ | ε_1_ /(σ_1_-σ_3_)_s_ |
| 4.4094 | 0.02524 | 3.7549 | 0.02416 | 4.1741 | 0.01945 |
| 4.5916 | 0.02571 | 3.9635 | 0.02459 | 4.3524 | 0.01935 |
| 4.7964 | 0.02213 | 4.1703 | 0.02374 | 4.5688 | 0.01985 |
| 5.0147 | 0.02483 | 4.3695 | 0.02361 | 4.7964 | 0.02065 |
| 5.2291 | 0.02409 | 4.5669 | 0.02395 | 5.0032 | 0.02159 |
| 5.4207 | 0.02565 | 4.7831 | 0.02368 | 5.1986 | 0.02133 |
| 5.6446 | 0.02681 | 4.9823 | 0.02455 | 5.4111 | 0.022 |
| 5.8362 | 0.02691 | 5.1892 | 0.02439 | 5.8171 | 0.02333 |
| 6.0696 | 0.02695 | 5.3922 | 0.02475 | 6.041 | 0.02379 |
| 6.2783 | 0.02751 | 5.5857 | 0.0248 | 6.2384 | 0.02374 |
| 6.4566 | 0.0282 | 5.8191 | 0.02597 | 6.4546 | 0.02537 |
| 6.6729 | 0.0283 | 6.0448 | 0.02638 | 6.6539 | 0.02133 |
| 6.8931 | 0.02707 | 6.2308 | 0.02629 | 6.8587 | 0.02384 |
| 7.1112 | 0.02625 | 6.4585 | 0.02795 | 7.0619 | 0.02238 |
| 7.2972 | 0.02752 | 6.6728 | 0.02819 | 7.2932 | 0.02586 |
| 7.5211 | 0.0278 | 6.8759 | 0.02784 | 7.4716 | 0.02697 |
| 7.7202 | 0.029 | 7.0618 | 0.02878 | 7.7108 | 0.02548 |
| 7.9308 | 0.02916 | 7.2649 | 0.02909 | 7.9175 | 0.02508 |
| 8.1319 | 0.03198 | 7.4831 | 0.02991 | 8.113 | 0.02811 |
| 8.3141 | 0.03305 | 7.6785 | 0.03067 | 8.3274 | 0.02739 |
| 8.5437 | 0.03268 | 7.9005 | 0.0318 | 8.5341 | 0.02882 |
| 8.7694 | 0.03403 | 8.1281 | 0.03332 | 8.722 | 0.02894 |
| 8.9745 | 0.03456 | 8.3331 | 0.03355 | 8.9477 | 0.02911 |
| 9.185 | 0.0353 | 8.5398 | 0.03322 | 9.1602 | 0.03108 |
| 9.3786 | 0.0351 | 8.7486 | 0.03529 | 9.3727 | 0.03122 |
| 9.6006 | 0.03474 | 8.9572 | 0.03451 | 9.5739 | 0.03018 |
| 9.7941 | 0.03208 | 9.1717 | 0.03512 | 9.7712 | 0.03103 |
| 10.0236 | 0.03523 | 9.3709 | 0.03418 | 9.9761 | 0.03047 |
| 10.2191 | 0.03727 | 9.5587 | 0.03626 | 10.1905 | 0.03243 |
| 10.4391 | 0.03745 | 9.7902 | 0.03669 | 10.3974 | 0.03293 |
| 10.6288 | 0.03465 | 9.9914 | 0.0379 |  |  |
| 10.8489 | 0.03763 | 10.1925 | 0.03914 |  |  |
| 11.0633 | 0.03738 | 10.3992 | 0.0375 |  |  |
| 11.2493 | 0.03791 | 10.6042 | 0.03866 |  |  |
|  |  | 10.8413 | 0.03916 |  |  |
|  |  | 11.0349 | 0.03985 |  |  |
|  |  | 11.2512 | 0.04162 |  |  |
|  |  | 11.4599 | 0.04203 |  |  |
|  |  | 11.6459 | 0.04248 |  |  |
|  |  | 11.8772 | 0.04336 |  |  |
|  |  | 12.0878 | 0.04276 |  |  |
|  |  | 12.2908 | 0.04102 |  |  |
|  |  | 12.4977 | 0.04246 |  |  |
|  |  | 12.7045 | 0.0412 |  |  |
|  |  | 12.9151 | 0.0421 |  |  |
|  |  | 13.1257 | 0.04237 |  |  |
