# Supplementary material for: Material composition and constitutive model development of red mud-based filler for highway tunnel invert filling applications: A comprehensive study
Source: PLoS One. 2025 Apr 16;20(4):e0321926. doi: 10.1371/journal.pone.0321926 (PMC12002488; doi:10.1371/journal.pone.0321926)
Supplement: S13 Table — Data of “ Kn line” of RMBF considering Sp. (DOCX) [file pone.0321926.s013.docx]

Table S13. The "Kn line" of RMBF considering Sp (Fig.18). Data of " Kn line" of RMBF considering Sp.

| 7d | | 14d | | 28d | |
| --- | --- | --- | --- | --- | --- |
| lg(σ_3_/p_a_) | lg(E_0_/p_a_) | lg(σ_3_/p_a_) | lg(E_0_/p_a_) | lg(σ_3_/p_a_) | lg(E_0_/p_a_) |
| -0.52288 | -0.23172 | -0.52288 | -0.25358 | -0.52288 | -0.14301 |
| -0.22185 | -0.15564 | -0.22185 | -0.20925 | -0.22185 | -0.10517 |
| -0.04576 | -0.04021 | -0.04576 | -0.00432 | -0.04576 | 0.02365 |
