# Supplementary material for: Material composition and constitutive model development of red mud-based filler for highway tunnel invert filling applications: A comprehensive study
Source: PLoS One. 2025 Apr 16;20(4):e0321926. doi: 10.1371/journal.pone.0321926 (PMC12002488; doi:10.1371/journal.pone.0321926)
Supplement: S14 Table — Data of mohr stress circle of RMBF considering Sp. (DOCX) [file pone.0321926.s014.docx]

Table S14. Mohr stress circle of RMBF considering Sp (Fig.19). Data of mohr stress circle of RMBF considering Sp.

(a) 7d

| 30kPa | | 60kPa | | 90kPa | |
| --- | --- | --- | --- | --- | --- |
| Normal stress-σ | Shear stress-τ | Normal stress-σ | Shear stress-τ | Normal stress-σ | Shear stress-τ |
| 150.385 | 0 | 205.56 | 0 | 209.44 | 0 |
| 153.12005 | 27.07562 | 209.10667 | 35.11021 | 212.76202 | 32.88632 |
| 155.8551 | 38.09485 | 212.65333 | 49.39935 | 216.08404 | 46.27039 |
| 158.59015 | 46.41535 | 216.2 | 60.18893 | 219.40606 | 56.37655 |
| 161.3252 | 53.31595 | 219.74667 | 69.13726 | 222.72808 | 64.7581 |
| 164.06025 | 59.29448 | 223.29333 | 76.8899 | 226.0501 | 72.01968 |
| 166.7953 | 64.60743 | 226.84 | 83.77944 | 229.37212 | 78.47284 |
| 169.53035 | 69.4078 | 230.38667 | 90.00431 | 232.69414 | 84.30342 |
| 172.2654 | 73.7957 | 233.93333 | 95.6943 | 236.01616 | 89.63301 |
| 175.00045 | 77.8409 | 237.48 | 100.9399 | 239.33818 | 94.54635 |
| 177.73551 | 81.5944 | 241.02667 | 105.80724 | 242.6602 | 99.10539 |
| 180.47056 | 85.0948 | 244.57333 | 110.34637 | 245.98222 | 103.35701 |
| 183.20561 | 88.37219 | 248.12 | 114.59631 | 249.30424 | 107.33776 |
| 185.94066 | 91.45054 | 251.66667 | 118.58814 | 252.62626 | 111.07675 |
| 188.67571 | 94.34933 | 255.21333 | 122.34715 | 255.94828 | 114.59766 |
| 191.41076 | 97.08466 | 258.76 | 125.89418 | 259.2703 | 117.92002 |
| 194.14581 | 99.66998 | 262.30667 | 129.24668 | 262.59232 | 121.06018 |
| 196.88086 | 102.11669 | 265.85333 | 132.41944 | 265.91434 | 124.03197 |
| 199.61591 | 104.43453 | 269.4 | 135.42509 | 269.23636 | 126.84724 |
| 202.35096 | 106.6319 | 272.94667 | 138.27452 | 272.55838 | 129.51619 |
| 205.08601 | 108.71611 | 276.49333 | 140.97721 | 275.8804 | 132.04769 |
| 207.82106 | 110.69355 | 280.04 | 143.54145 | 279.20242 | 134.44951 |
| 210.55611 | 112.56984 | 283.58667 | 145.97453 | 282.52444 | 136.72848 |
| 213.29116 | 114.34997 | 287.13333 | 148.28291 | 285.84646 | 138.89064 |
| 216.02621 | 116.03837 | 290.68 | 150.47232 | 289.16848 | 140.94138 |
| 218.76126 | 117.63897 | 294.22667 | 152.5479 | 292.49051 | 142.88549 |
| 221.49631 | 119.15532 | 297.77333 | 154.51423 | 295.81253 | 144.72727 |
| 224.23136 | 120.59061 | 301.32 | 156.37543 | 299.13455 | 146.47058 |
| 226.96641 | 121.94768 | 304.86667 | 158.1352 | 302.45657 | 148.11889 |
| 229.70146 | 123.22913 | 308.41333 | 159.79692 | 305.77859 | 149.67535 |
| 232.43652 | 124.43728 | 311.96 | 161.36359 | 309.10061 | 151.14279 |
| 235.17157 | 125.57427 | 315.50667 | 162.83797 | 312.42263 | 152.52378 |
| 237.90662 | 126.64199 | 319.05333 | 164.22254 | 315.74465 | 153.82066 |
| 240.64167 | 127.6422 | 322.6 | 165.51956 | 319.06667 | 155.03552 |
| 243.37672 | 128.57647 | 326.14667 | 166.73106 | 322.38869 | 156.17029 |
| 246.11177 | 129.44622 | 329.69333 | 167.8589 | 325.71071 | 157.22669 |
| 248.84682 | 130.25274 | 333.24 | 168.90476 | 329.03273 | 158.20631 |
| 251.58187 | 130.99721 | 336.78667 | 169.87015 | 332.35475 | 159.11055 |
| 254.31692 | 131.68068 | 340.33333 | 170.75644 | 335.67677 | 159.9407 |
| 257.05197 | 132.30409 | 343.88 | 171.56484 | 338.99879 | 160.6979 |
| 259.78702 | 132.86829 | 347.42667 | 172.29647 | 342.32081 | 161.38318 |
| 262.52207 | 133.37403 | 350.97333 | 172.95228 | 345.64283 | 161.99746 |
| 265.25712 | 133.82197 | 354.52 | 173.53315 | 348.96485 | 162.54153 |
| 267.99217 | 134.21269 | 358.06667 | 174.03982 | 352.28687 | 163.0161 |
| 270.72722 | 134.54669 | 361.61333 | 174.47293 | 355.60889 | 163.42179 |
| 273.46227 | 134.8244 | 365.16 | 174.83304 | 358.93091 | 163.75909 |
| 276.19732 | 135.04615 | 368.70667 | 175.12059 | 362.25293 | 164.02843 |
| 278.93237 | 135.21222 | 372.25333 | 175.33595 | 365.57495 | 164.23014 |
| 281.66742 | 135.32283 | 375.8 | 175.47938 | 368.89697 | 164.36448 |
| 284.40247 | 135.37809 | 379.34667 | 175.55104 | 372.21899 | 164.43161 |
| 287.13753 | 135.37809 | 382.89333 | 175.55104 | 375.54101 | 164.43161 |
| 289.87258 | 135.32283 | 386.44 | 175.47938 | 378.86303 | 164.36448 |
| 292.60763 | 135.21222 | 389.98667 | 175.33595 | 382.18505 | 164.23014 |
| 295.34268 | 135.04615 | 393.53333 | 175.12059 | 385.50707 | 164.02843 |
| 298.07773 | 134.8244 | 397.08 | 174.83304 | 388.82909 | 163.75909 |
| 300.81278 | 134.54669 | 400.62667 | 174.47293 | 392.15111 | 163.42179 |
| 303.54783 | 134.21269 | 404.17333 | 174.03982 | 395.47313 | 163.0161 |
| 306.28288 | 133.82197 | 407.72 | 173.53315 | 398.79515 | 162.54153 |
| 309.01793 | 133.37403 | 411.26667 | 172.95228 | 402.11717 | 161.99746 |
| 311.75298 | 132.86829 | 414.81333 | 172.29647 | 405.43919 | 161.38318 |
| 314.48803 | 132.30409 | 418.36 | 171.56484 | 408.76121 | 160.6979 |
| 317.22308 | 131.68068 | 421.90667 | 170.75644 | 412.08323 | 159.9407 |
| 319.95813 | 130.99721 | 425.45333 | 169.87015 | 415.40525 | 159.11055 |
| 322.69318 | 130.25274 | 429 | 168.90476 | 418.72727 | 158.20631 |
| 325.42823 | 129.44622 | 432.54667 | 167.8589 | 422.04929 | 157.22669 |
| 328.16328 | 128.57647 | 436.09333 | 166.73106 | 425.37131 | 156.17029 |
| 330.89833 | 127.6422 | 439.64 | 165.51956 | 428.69333 | 155.03552 |
| 333.63338 | 126.64199 | 443.18667 | 164.22254 | 432.01535 | 153.82066 |
| 336.36843 | 125.57427 | 446.73333 | 162.83797 | 435.33737 | 152.52378 |
| 339.10348 | 124.43728 | 450.28 | 161.36359 | 438.65939 | 151.14279 |
| 341.83854 | 123.22913 | 453.82667 | 159.79692 | 441.98141 | 149.67535 |
| 344.57359 | 121.94768 | 457.37333 | 158.1352 | 445.30343 | 148.11889 |
| 347.30864 | 120.59061 | 460.92 | 156.37543 | 448.62545 | 146.47058 |
| 350.04369 | 119.15532 | 464.46667 | 154.51423 | 451.94747 | 144.72727 |
| 352.77874 | 117.63897 | 468.01333 | 152.5479 | 455.26949 | 142.88549 |
| 355.51379 | 116.03837 | 471.56 | 150.47232 | 458.59152 | 140.94138 |
| 358.24884 | 114.34997 | 475.10667 | 148.28291 | 461.91354 | 138.89064 |
| 360.98389 | 112.56984 | 478.65333 | 145.97453 | 465.23556 | 136.72848 |
| 363.71894 | 110.69355 | 482.2 | 143.54145 | 468.55758 | 134.44951 |
| 366.45399 | 108.71611 | 485.74667 | 140.97721 | 471.8796 | 132.04769 |
| 369.18904 | 106.6319 | 489.29333 | 138.27452 | 475.20162 | 129.51619 |
| 371.92409 | 104.43453 | 492.84 | 135.42509 | 478.52364 | 126.84724 |
| 374.65914 | 102.11669 | 496.38667 | 132.41944 | 481.84566 | 124.03197 |
| 377.39419 | 99.66998 | 499.93333 | 129.24668 | 485.16768 | 121.06018 |
| 380.12924 | 97.08466 | 503.48 | 125.89418 | 488.4897 | 117.92002 |
| 382.86429 | 94.34933 | 507.02667 | 122.34715 | 491.81172 | 114.59766 |
| 385.59934 | 91.45054 | 510.57333 | 118.58814 | 495.13374 | 111.07675 |
| 388.33439 | 88.37219 | 514.12 | 114.59631 | 498.45576 | 107.33776 |
| 391.06944 | 85.0948 | 517.66667 | 110.34637 | 501.77778 | 103.35701 |
| 393.80449 | 81.5944 | 521.21333 | 105.80724 | 505.0998 | 99.10539 |
| 396.53955 | 77.8409 | 524.76 | 100.9399 | 508.42182 | 94.54635 |
| 399.2746 | 73.7957 | 528.30667 | 95.6943 | 511.74384 | 89.63301 |
| 402.00965 | 69.4078 | 531.85333 | 90.00431 | 515.06586 | 84.30342 |
| 404.7447 | 64.60743 | 535.4 | 83.77944 | 518.38788 | 78.47284 |
| 407.47975 | 59.29448 | 538.94667 | 76.8899 | 521.7099 | 72.01968 |
| 410.2148 | 53.31595 | 542.49333 | 69.13726 | 525.03192 | 64.7581 |
| 412.94985 | 46.41535 | 546.04 | 60.18893 | 528.35394 | 56.37655 |
| 415.6849 | 38.09485 | 549.58667 | 49.39935 | 531.67596 | 46.27039 |
| 418.41995 | 27.07562 | 553.13333 | 35.11021 | 534.99798 | 32.88632 |
| 421.155 | 0 | 556.68 | 0 | 538.32 | 0 |

(b) 14d

| 30kPa | | 60kPa | | 90kPa | |
| --- | --- | --- | --- | --- | --- |
| Normal stress-σ | Shear stress-τ | Normal stress-σ | Shear stress-τ | Normal stress-σ | Shear stress-τ |
| 143.595 | 0 | 164.955 | 0 | 199.85 | 0 |
| 146.19288 | 25.71769 | 167.68136 | 26.98962 | 202.97828 | 30.96842 |
| 148.79076 | 36.18426 | 170.40773 | 37.97385 | 206.10657 | 43.57194 |
| 151.38864 | 44.08746 | 173.13409 | 46.26793 | 209.23485 | 53.08872 |
| 153.98652 | 50.64198 | 175.86045 | 53.14661 | 212.36313 | 60.98146 |
| 156.58439 | 56.32067 | 178.58682 | 59.10616 | 215.49141 | 67.81956 |
| 159.18227 | 61.36715 | 181.31318 | 64.40223 | 218.6197 | 73.89637 |
| 161.78015 | 65.92677 | 184.03955 | 69.18735 | 221.74798 | 79.38692 |
| 164.37803 | 70.0946 | 186.76591 | 73.56131 | 224.87626 | 84.40569 |
| 166.97591 | 73.93693 | 189.49227 | 77.59367 | 228.00455 | 89.03249 |
| 169.57379 | 77.50217 | 192.21864 | 81.33525 | 231.13283 | 93.32565 |
| 172.17167 | 80.82702 | 194.945 | 84.82453 | 234.26111 | 97.32932 |
| 174.76955 | 83.94003 | 197.67136 | 88.09151 | 237.38939 | 101.07791 |
| 177.36742 | 86.86399 | 200.39773 | 91.16008 | 240.51768 | 104.59885 |
| 179.9653 | 89.61741 | 203.12409 | 94.04967 | 243.64596 | 107.91442 |
| 182.56318 | 92.21555 | 205.85045 | 96.77631 | 246.77424 | 111.04302 |
| 185.16106 | 94.67121 | 208.57682 | 99.35342 | 249.90253 | 114.00005 |
| 187.75894 | 96.99521 | 211.30318 | 101.79236 | 253.03081 | 116.79854 |
| 190.35682 | 99.1968 | 214.02955 | 104.10283 | 256.15909 | 119.44962 |
| 192.9547 | 101.28396 | 216.75591 | 106.29322 | 259.28737 | 121.96292 |
| 195.55258 | 103.26364 | 219.48227 | 108.37081 | 262.41566 | 124.34678 |
| 198.15045 | 105.1419 | 222.20864 | 110.34197 | 265.54394 | 126.60853 |
| 200.74833 | 106.9241 | 224.935 | 112.21231 | 268.67222 | 128.75459 |
| 203.34621 | 108.61495 | 227.66136 | 113.98678 | 271.80051 | 130.79066 |
| 205.94409 | 110.21866 | 230.38773 | 115.66981 | 274.92879 | 132.7218 |
| 208.54197 | 111.73899 | 233.11409 | 117.26533 | 278.05707 | 134.55253 |
| 211.13985 | 113.1793 | 235.84045 | 118.77687 | 281.18535 | 136.2869 |
| 213.73773 | 114.54259 | 238.56682 | 120.2076 | 284.31364 | 137.92854 |
| 216.33561 | 115.8316 | 241.29318 | 121.56036 | 287.44192 | 139.48073 |
| 218.93348 | 117.04878 | 244.01955 | 122.83773 | 290.5702 | 140.94641 |
| 221.53136 | 118.19635 | 246.74591 | 124.04206 | 293.69848 | 142.32827 |
| 224.12924 | 119.27631 | 249.47227 | 125.17543 | 296.82677 | 143.62873 |
| 226.72712 | 120.29048 | 252.19864 | 126.23976 | 299.95505 | 144.84997 |
| 229.325 | 121.24053 | 254.925 | 127.23679 | 303.08333 | 145.99398 |
| 231.92288 | 122.12794 | 257.65136 | 128.16809 | 306.21162 | 147.06257 |
| 234.52076 | 122.95407 | 260.37773 | 129.03508 | 309.3399 | 148.05737 |
| 237.11864 | 123.72014 | 263.10409 | 129.83904 | 312.46818 | 148.97985 |
| 239.71652 | 124.42728 | 265.83045 | 130.58115 | 315.59646 | 149.83136 |
| 242.31439 | 125.07647 | 268.55682 | 131.26245 | 318.72475 | 150.61309 |
| 244.91227 | 125.66861 | 271.28318 | 131.88388 | 321.85303 | 151.32613 |
| 247.51015 | 126.20451 | 274.00955 | 132.44629 | 324.98131 | 151.97145 |
| 250.10803 | 126.68489 | 276.73591 | 132.95042 | 328.1096 | 152.5499 |
| 252.70591 | 127.11036 | 279.46227 | 133.39694 | 331.23788 | 153.06225 |
| 255.30379 | 127.48149 | 282.18864 | 133.78642 | 334.36616 | 153.50914 |
| 257.90167 | 127.79874 | 284.915 | 134.11936 | 337.49444 | 153.89117 |
| 260.49955 | 128.06251 | 287.64136 | 134.39618 | 340.62273 | 154.2088 |
| 263.09742 | 128.27314 | 290.36773 | 134.61722 | 343.75101 | 154.46243 |
| 265.6953 | 128.43089 | 293.09409 | 134.78277 | 346.87929 | 154.65238 |
| 268.29318 | 128.53594 | 295.82045 | 134.89302 | 350.00758 | 154.77889 |
| 270.89106 | 128.58844 | 298.54682 | 134.94812 | 353.13586 | 154.8421 |
| 273.48894 | 128.58844 | 301.27318 | 134.94812 | 356.26414 | 154.8421 |
| 276.08682 | 128.53594 | 303.99955 | 134.89302 | 359.39242 | 154.77889 |
| 278.6847 | 128.43089 | 306.72591 | 134.78277 | 362.52071 | 154.65238 |
| 281.28258 | 128.27314 | 309.45227 | 134.61722 | 365.64899 | 154.46243 |
| 283.88045 | 128.06251 | 312.17864 | 134.39618 | 368.77727 | 154.2088 |
| 286.47833 | 127.79874 | 314.905 | 134.11936 | 371.90556 | 153.89117 |
| 289.07621 | 127.48149 | 317.63136 | 133.78642 | 375.03384 | 153.50914 |
| 291.67409 | 127.11036 | 320.35773 | 133.39694 | 378.16212 | 153.06225 |
| 294.27197 | 126.68489 | 323.08409 | 132.95042 | 381.2904 | 152.5499 |
| 296.86985 | 126.20451 | 325.81045 | 132.44629 | 384.41869 | 151.97145 |
| 299.46773 | 125.66861 | 328.53682 | 131.88388 | 387.54697 | 151.32613 |
| 302.06561 | 125.07647 | 331.26318 | 131.26245 | 390.67525 | 150.61309 |
| 304.66348 | 124.42728 | 333.98955 | 130.58115 | 393.80354 | 149.83136 |
| 307.26136 | 123.72014 | 336.71591 | 129.83904 | 396.93182 | 148.97985 |
| 309.85924 | 122.95407 | 339.44227 | 129.03508 | 400.0601 | 148.05737 |
| 312.45712 | 122.12794 | 342.16864 | 128.16809 | 403.18838 | 147.06257 |
| 315.055 | 121.24053 | 344.895 | 127.23679 | 406.31667 | 145.99398 |
| 317.65288 | 120.29048 | 347.62136 | 126.23976 | 409.44495 | 144.84997 |
| 320.25076 | 119.27631 | 350.34773 | 125.17543 | 412.57323 | 143.62873 |
| 322.84864 | 118.19635 | 353.07409 | 124.04206 | 415.70152 | 142.32827 |
| 325.44652 | 117.04878 | 355.80045 | 122.83773 | 418.8298 | 140.94641 |
| 328.04439 | 115.8316 | 358.52682 | 121.56036 | 421.95808 | 139.48073 |
| 330.64227 | 114.54259 | 361.25318 | 120.2076 | 425.08636 | 137.92854 |
| 333.24015 | 113.1793 | 363.97955 | 118.77687 | 428.21465 | 136.2869 |
| 335.83803 | 111.73899 | 366.70591 | 117.26533 | 431.34293 | 134.55253 |
| 338.43591 | 110.21866 | 369.43227 | 115.66981 | 434.47121 | 132.7218 |
| 341.03379 | 108.61495 | 372.15864 | 113.98678 | 437.59949 | 130.79066 |
| 343.63167 | 106.9241 | 374.885 | 112.21231 | 440.72778 | 128.75459 |
| 346.22955 | 105.1419 | 377.61136 | 110.34197 | 443.85606 | 126.60853 |
| 348.82742 | 103.26364 | 380.33773 | 108.37081 | 446.98434 | 124.34678 |
| 351.4253 | 101.28396 | 383.06409 | 106.29322 | 450.11263 | 121.96292 |
| 354.02318 | 99.1968 | 385.79045 | 104.10283 | 453.24091 | 119.44962 |
| 356.62106 | 96.99521 | 388.51682 | 101.79236 | 456.36919 | 116.79854 |
| 359.21894 | 94.67121 | 391.24318 | 99.35342 | 459.49747 | 114.00005 |
| 361.81682 | 92.21555 | 393.96955 | 96.77631 | 462.62576 | 111.04302 |
| 364.4147 | 89.61741 | 396.69591 | 94.04967 | 465.75404 | 107.91442 |
| 367.01258 | 86.86399 | 399.42227 | 91.16008 | 468.88232 | 104.59885 |
| 369.61045 | 83.94003 | 402.14864 | 88.09151 | 472.01061 | 101.07791 |
| 372.20833 | 80.82702 | 404.875 | 84.82453 | 475.13889 | 97.32932 |
| 374.80621 | 77.50217 | 407.60136 | 81.33525 | 478.26717 | 93.32565 |
| 377.40409 | 73.93693 | 410.32773 | 77.59367 | 481.39545 | 89.03249 |
| 380.00197 | 70.0946 | 413.05409 | 73.56131 | 484.52374 | 84.40569 |
| 382.59985 | 65.92677 | 415.78045 | 69.18735 | 487.65202 | 79.38692 |
| 385.19773 | 61.36715 | 418.50682 | 64.40223 | 490.7803 | 73.89637 |
| 387.79561 | 56.32067 | 421.23318 | 59.10616 | 493.90859 | 67.81956 |
| 390.39348 | 50.64198 | 423.95955 | 53.14661 | 497.03687 | 60.98146 |
| 392.99136 | 44.08746 | 426.68591 | 46.26793 | 500.16515 | 53.08872 |
| 395.58924 | 36.18426 | 429.41227 | 37.97385 | 503.29343 | 43.57194 |
| 398.18712 | 25.71769 | 432.13864 | 26.98962 | 506.42172 | 30.96842 |
| 400.785 | 0 | 434.865 | 0 | 509.55 | 0 |

(c) 28d

| 30kPa | | 60kPa | | 90kPa | |
| --- | --- | --- | --- | --- | --- |
| Normal stress-σ | Shear stress-τ | Normal stress-σ | Shear stress-τ | Normal stress-σ | Shear stress-τ |
| 171.265 | 0 | 192.67 | 0 | 199.895 | 0 |
| 174.42187 | 31.25141 | 195.95626 | 32.53234 | 203.02419 | 30.97742 |
| 177.57874 | 43.97009 | 199.24253 | 45.77234 | 206.15338 | 43.5846 |
| 180.73561 | 53.57384 | 202.52879 | 55.76973 | 209.28258 | 53.10415 |
| 183.89247 | 61.5387 | 205.81505 | 64.06105 | 212.41177 | 60.99918 |
| 187.04934 | 68.43928 | 209.10131 | 71.24448 | 215.54096 | 67.83926 |
| 190.20621 | 74.57163 | 212.38758 | 77.62817 | 218.67015 | 73.91785 |
| 193.36308 | 80.11235 | 215.67384 | 83.396 | 221.79934 | 79.40999 |
| 196.51995 | 85.17697 | 218.9601 | 88.66821 | 224.92854 | 84.43021 |
| 199.67682 | 89.84606 | 222.24636 | 93.52867 | 228.05773 | 89.05836 |
| 202.83369 | 94.17845 | 225.53263 | 98.03864 | 231.18692 | 93.35277 |
| 205.99056 | 98.2187 | 228.81889 | 102.2445 | 234.31611 | 97.3576 |
| 209.14742 | 102.00155 | 232.10515 | 106.1824 | 237.4453 | 101.10729 |
| 212.30429 | 105.55466 | 235.39141 | 109.88114 | 240.57449 | 104.62925 |
| 215.46116 | 108.90053 | 238.67768 | 113.36415 | 243.70369 | 107.94578 |
| 218.61803 | 112.05772 | 241.96394 | 116.65075 | 246.83288 | 111.07529 |
| 221.7749 | 115.04177 | 245.2502 | 119.75711 | 249.96207 | 114.03318 |
| 224.93177 | 117.86583 | 248.53646 | 122.69692 | 253.09126 | 116.83248 |
| 228.08864 | 120.54114 | 251.82273 | 125.48188 | 256.22045 | 119.48433 |
| 231.24551 | 123.0774 | 255.10899 | 128.1221 | 259.34965 | 121.99836 |
| 234.40237 | 125.48305 | 258.39525 | 130.62636 | 262.47884 | 124.38292 |
| 237.55924 | 127.76546 | 261.68152 | 133.00232 | 265.60803 | 126.64532 |
| 240.71611 | 129.93113 | 264.96778 | 135.25676 | 268.73722 | 128.792 |
| 243.87298 | 131.98581 | 268.25404 | 137.39565 | 271.86641 | 130.82867 |
| 247.02985 | 133.9346 | 271.5403 | 139.42432 | 274.99561 | 132.76037 |
| 250.18672 | 135.78206 | 274.82657 | 141.3475 | 278.1248 | 134.59163 |
| 253.34359 | 137.53227 | 278.11283 | 143.16946 | 281.25399 | 136.32651 |
| 256.50045 | 139.18891 | 281.39909 | 144.894 | 284.38318 | 137.96862 |
| 259.65732 | 140.75528 | 284.68535 | 146.52457 | 287.51237 | 139.52126 |
| 262.81419 | 142.23436 | 287.97162 | 148.06428 | 290.64157 | 140.98737 |
| 265.97106 | 143.62885 | 291.25788 | 149.51592 | 293.77076 | 142.36964 |
| 269.12793 | 144.94119 | 294.54414 | 150.88205 | 296.89995 | 143.67047 |
| 272.2848 | 146.17359 | 297.8304 | 152.16496 | 300.02914 | 144.89206 |
| 275.44167 | 147.32805 | 301.11667 | 153.36675 | 303.15833 | 146.03641 |
| 278.59854 | 148.40641 | 304.40293 | 154.4893 | 306.28753 | 147.10531 |
| 281.7554 | 149.4103 | 307.68919 | 155.53434 | 309.41672 | 148.10039 |
| 284.91227 | 150.34121 | 310.97545 | 156.50341 | 312.54591 | 149.02315 |
| 288.06914 | 151.2005 | 314.26172 | 157.39791 | 315.6751 | 149.8749 |
| 291.22601 | 151.98938 | 317.54798 | 158.21913 | 318.80429 | 150.65686 |
| 294.38288 | 152.70893 | 320.83424 | 158.96818 | 321.93348 | 151.37011 |
| 297.53975 | 153.36015 | 324.12051 | 159.64608 | 325.06268 | 152.01562 |
| 300.69662 | 153.94389 | 327.40677 | 160.25375 | 328.19187 | 152.59423 |
| 303.85348 | 154.46091 | 330.69303 | 160.79196 | 331.32106 | 153.10673 |
| 307.01035 | 154.91189 | 333.97929 | 161.26143 | 334.45025 | 153.55376 |
| 310.16722 | 155.29741 | 337.26556 | 161.66275 | 337.57944 | 153.93589 |
| 313.32409 | 155.61794 | 340.55182 | 161.99642 | 340.70864 | 154.25361 |
| 316.48096 | 155.87389 | 343.83808 | 162.26286 | 343.83783 | 154.50732 |
| 319.63783 | 156.06558 | 347.12434 | 162.4624 | 346.96702 | 154.69732 |
| 322.7947 | 156.19324 | 350.41061 | 162.5953 | 350.09621 | 154.82387 |
| 325.95157 | 156.25703 | 353.69687 | 162.6617 | 353.2254 | 154.8871 |
| 329.10843 | 156.25703 | 356.98313 | 162.6617 | 356.3546 | 154.8871 |
| 332.2653 | 156.19324 | 360.26939 | 162.5953 | 359.48379 | 154.82387 |
| 335.42217 | 156.06558 | 363.55566 | 162.4624 | 362.61298 | 154.69732 |
| 338.57904 | 155.87389 | 366.84192 | 162.26286 | 365.74217 | 154.50732 |
| 341.73591 | 155.61794 | 370.12818 | 161.99642 | 368.87136 | 154.25361 |
| 344.89278 | 155.29741 | 373.41444 | 161.66275 | 372.00056 | 153.93589 |
| 348.04965 | 154.91189 | 376.70071 | 161.26143 | 375.12975 | 153.55376 |
| 351.20652 | 154.46091 | 379.98697 | 160.79196 | 378.25894 | 153.10673 |
| 354.36338 | 153.94389 | 383.27323 | 160.25375 | 381.38813 | 152.59423 |
| 357.52025 | 153.36015 | 386.55949 | 159.64608 | 384.51732 | 152.01562 |
| 360.67712 | 152.70893 | 389.84576 | 158.96818 | 387.64652 | 151.37011 |
| 363.83399 | 151.98938 | 393.13202 | 158.21913 | 390.77571 | 150.65686 |
| 366.99086 | 151.2005 | 396.41828 | 157.39791 | 393.9049 | 149.8749 |
| 370.14773 | 150.34121 | 399.70455 | 156.50341 | 397.03409 | 149.02315 |
| 373.3046 | 149.4103 | 402.99081 | 155.53434 | 400.16328 | 148.10039 |
| 376.46146 | 148.40641 | 406.27707 | 154.4893 | 403.29247 | 147.10531 |
| 379.61833 | 147.32805 | 409.56333 | 153.36675 | 406.42167 | 146.03641 |
| 382.7752 | 146.17359 | 412.8496 | 152.16496 | 409.55086 | 144.89206 |
| 385.93207 | 144.94119 | 416.13586 | 150.88205 | 412.68005 | 143.67047 |
| 389.08894 | 143.62885 | 419.42212 | 149.51592 | 415.80924 | 142.36964 |
| 392.24581 | 142.23436 | 422.70838 | 148.06428 | 418.93843 | 140.98737 |
| 395.40268 | 140.75528 | 425.99465 | 146.52457 | 422.06763 | 139.52126 |
| 398.55955 | 139.18891 | 429.28091 | 144.894 | 425.19682 | 137.96862 |
| 401.71641 | 137.53227 | 432.56717 | 143.16946 | 428.32601 | 136.32651 |
| 404.87328 | 135.78206 | 435.85343 | 141.3475 | 431.4552 | 134.59163 |
| 408.03015 | 133.9346 | 439.1397 | 139.42432 | 434.58439 | 132.76037 |
| 411.18702 | 131.98581 | 442.42596 | 137.39565 | 437.71359 | 130.82867 |
| 414.34389 | 129.93113 | 445.71222 | 135.25676 | 440.84278 | 128.792 |
| 417.50076 | 127.76546 | 448.99848 | 133.00232 | 443.97197 | 126.64532 |
| 420.65763 | 125.48305 | 452.28475 | 130.62636 | 447.10116 | 124.38292 |
| 423.81449 | 123.0774 | 455.57101 | 128.1221 | 450.23035 | 121.99836 |
| 426.97136 | 120.54114 | 458.85727 | 125.48188 | 453.35955 | 119.48433 |
| 430.12823 | 117.86583 | 462.14354 | 122.69692 | 456.48874 | 116.83248 |
| 433.2851 | 115.04177 | 465.4298 | 119.75711 | 459.61793 | 114.03318 |
| 436.44197 | 112.05772 | 468.71606 | 116.65075 | 462.74712 | 111.07529 |
| 439.59884 | 108.90053 | 472.00232 | 113.36415 | 465.87631 | 107.94578 |
| 442.75571 | 105.55466 | 475.28859 | 109.88114 | 469.00551 | 104.62925 |
| 445.91258 | 102.00155 | 478.57485 | 106.1824 | 472.1347 | 101.10729 |
| 449.06944 | 98.2187 | 481.86111 | 102.2445 | 475.26389 | 97.3576 |
| 452.22631 | 94.17845 | 485.14737 | 98.03864 | 478.39308 | 93.35277 |
| 455.38318 | 89.84606 | 488.43364 | 93.52867 | 481.52227 | 89.05836 |
| 458.54005 | 85.17697 | 491.7199 | 88.66821 | 484.65146 | 84.43021 |
| 461.69692 | 80.11235 | 495.00616 | 83.396 | 487.78066 | 79.40999 |
| 464.85379 | 74.57163 | 498.29242 | 77.62817 | 490.90985 | 73.91785 |
| 468.01066 | 68.43928 | 501.57869 | 71.24448 | 494.03904 | 67.83926 |
| 471.16753 | 61.5387 | 504.86495 | 64.06105 | 497.16823 | 60.99918 |
| 474.32439 | 53.57384 | 508.15121 | 55.76973 | 500.29742 | 53.10415 |
| 477.48126 | 43.97009 | 511.43747 | 45.77234 | 503.42662 | 43.5846 |
| 480.63813 | 31.25141 | 514.72374 | 32.53234 | 506.55581 | 30.97742 |
| 483.795 | 0 | 518.01 | 0 | 509.685 | 0 |
