# Supplementary material for: Material composition and constitutive model development of red mud-based filler for highway tunnel invert filling applications: A comprehensive study
Source: PLoS One. 2025 Apr 16;20(4):e0321926. doi: 10.1371/journal.pone.0321926 (PMC12002488; doi:10.1371/journal.pone.0321926)
Supplement: S15 Table — Data of “Df line” of RMBF considering Sp. (DOCX) [file pone.0321926.s015.docx]

Table S15. The "Df line" of RMBF considering Sp (Fig.20). Data of "Df line" of RMBF considering Sp.

(a) 7d

| 30kPa | | 60kPa | | 90kPa | |
| --- | --- | --- | --- | --- | --- |
| ε_3_ | ε_3/_ε_1_ | ε_3_ | ε_3/_ε_1_ | ε_3_ | ε_3/_ε_1_ |
| 2.7317 | 0.58716 | 2.7317 | 0.58716 | 1.4951 | 0.61503 |
| 2.911 | 0.59675 | 2.911 | 0.59675 | 1.9094 | 0.62649 |
| 3.0705 | 0.60724 | 3.0705 | 0.60724 | 2.3869 | 0.63958 |
| 3.2312 | 0.61413 | 3.2312 | 0.61413 | 2.6798 | 0.6468 |
| 3.3994 | 0.62039 | 3.3994 | 0.62039 | 2.9831 | 0.65124 |
| 3.5583 | 0.62492 | 3.5583 | 0.62492 | 3.3304 | 0.65677 |
| 3.7117 | 0.62984 | 3.7117 | 0.62984 | 3.5739 | 0.66615 |
| 3.869 | 0.63406 | 3.869 | 0.63406 | 3.7621 | 0.67899 |
| 4.0347 | 0.63783 | 4.0347 | 0.63783 | 4.0537 | 0.69542 |
| 4.1971 | 0.64175 | 4.1971 | 0.64175 | 4.3672 | 0.71954 |
| 4.3659 | 0.64691 | 4.3659 | 0.64691 | 4.5493 | 0.74743 |
| 4.6416 | 0.66658 | 4.6416 | 0.66658 | 4.7785 | 0.77201 |
| 4.9769 | 0.69357 | 4.9769 | 0.69357 | 4.9879 | 0.79459 |
| 5.2718 | 0.7139 | 5.2718 | 0.7139 | 5.1813 | 0.81248 |
| 5.5695 | 0.73294 | 5.5695 | 0.73294 | 5.3452 | 0.82621 |
| 5.8488 | 0.74912 | 5.8488 | 0.74912 | 5.5197 | 0.83975 |
| 6.08 | 0.76134 | 6.08 | 0.76134 | 5.7115 | 0.85115 |
| 6.3266 | 0.77132 | 6.3266 | 0.77132 | 5.8705 | 0.86168 |
| 6.5708 | 0.77946 | 6.5708 | 0.77946 | 6.0539 | 0.87205 |
| 6.7971 | 0.787 | 6.7971 | 0.787 | 6.2442 | 0.88377 |
| 7.0076 | 0.79342 | 7.0076 | 0.79342 | 6.4168 | 0.89354 |
| 7.2286 | 0.79922 | 7.2286 | 0.79922 | 6.6079 | 0.90282 |
| 7.4028 | 0.80479 | 7.4028 | 0.80479 | 6.8023 | 0.91 |
| 7.6332 | 0.80769 | 7.6332 | 0.80769 | 7.0048 | 0.91581 |
| 7.859 | 0.81234 | 7.859 | 0.81234 | 7.2061 | 0.92265 |
| 8.0488 | 0.81532 | 8.0488 | 0.81532 | 7.3954 | 0.92839 |
| 8.2634 | 0.81912 | 8.2634 | 0.81912 | 7.58 | 0.93267 |
| 8.4618 | 0.82254 | 8.4618 | 0.82254 | 7.7526 | 0.93709 |
| 8.6611 | 0.82548 | 8.6611 | 0.82548 | 7.9406 | 0.94032 |
| 8.8644 | 0.8288 | 8.8644 | 0.8288 | 8.1168 | 0.94452 |
| 9.0879 | 0.83171 | 9.0879 | 0.83171 | 8.289 | 0.94741 |
| 9.2703 | 0.83478 | 9.2703 | 0.83478 | 8.4749 | 0.94991 |
| 9.4915 | 0.83668 | 9.4915 | 0.83668 | 8.6475 | 0.9526 |
| 9.6867 | 0.8386 | 9.6867 | 0.8386 | 8.8115 | 0.95493 |
| 9.88 | 0.8411 | 9.88 | 0.8411 | 9.0094 | 0.9564 |
| 10.0892 | 0.84352 | 10.0892 | 0.84352 | 9.1969 | 0.95915 |
| 10.2892 | 0.84562 | 10.2892 | 0.84562 | 9.3608 | 0.96241 |
| 10.4754 | 0.84783 | 10.4754 | 0.84783 | 9.548 | 0.96479 |
| 10.6902 | 0.8497 | 10.6902 | 0.8497 | 9.7322 | 0.96867 |
| 10.8931 | 0.85144 | 10.8931 | 0.85144 | 9.902 | 0.97391 |
| 11.1028 | 0.85365 | 11.1028 | 0.85365 | 10.0821 | 0.97775 |
| 11.2997 | 0.85556 | 11.2997 | 0.85556 | 10.2596 | 0.98085 |
| 11.4989 | 0.85783 | 11.4989 | 0.85783 | 10.4529 | 0.9827 |
| 11.6977 | 0.85952 | 11.6977 | 0.85952 | 10.6429 | 0.9853 |
| 11.9025 | 0.861 | 11.9025 | 0.861 | 10.7997 | 0.98664 |
| 12.1023 | 0.86255 | 12.1023 | 0.86255 | 10.9938 | 0.98854 |
| 12.3202 | 0.86418 | 12.3202 | 0.86418 | 11.1585 | 0.99019 |
| 12.5115 | 0.86606 | 12.5115 | 0.86606 | 11.3572 | 0.99136 |
| 12.7135 | 0.86752 | 12.7135 | 0.86752 | 11.5416 | 0.99249 |
| 12.9268 | 0.86913 | 12.9268 | 0.86913 | 11.7097 | 0.99265 |
| 13.5595 | 0.90435 | 13.5595 | 0.90435 | 11.914 | 0.99275 |
|  |  |  |  | 12.0959 | 0.99374 |
|  |  |  |  | 12.2892 | 0.99469 |
|  |  |  |  | 12.4754 | 0.99523 |
|  |  |  |  | 12.658 | 0.9956 |
|  |  |  |  | 12.8324 | 0.99612 |
|  |  |  |  | 13.0056 | 0.99714 |
|  |  |  |  | 13.1925 | 0.998 |
|  |  |  |  | 13.3895 | 0.99882 |

(b) 14d

| 30kPa | | 60kPa | | 90kPa | |
| --- | --- | --- | --- | --- | --- |
| ε_3_ | ε_3/_ε_1_ | ε_3_ | ε_3/_ε_1_ | ε_3_ | ε_3/_ε_1_ |
| 0.3282 | 0.49279 | 0.4479 | 0.39543 | 1.4511 | 0.51503 |
| 0.4537 | 0.50983 | 0.5471 | 0.41668 | 1.5723 | 0.52649 |
| 0.5755 | 0.52204 | 0.6655 | 0.43519 | 1.7383 | 0.53958 |
| 0.6824 | 0.52891 | 0.7891 | 0.45107 | 1.8695 | 0.5468 |
| 0.8008 | 0.53422 | 0.896 | 0.46117 | 1.9882 | 0.55124 |
| 0.9197 | 0.53919 | 1.0128 | 0.47195 | 2.1455 | 0.55677 |
| 1.0512 | 0.54261 | 1.1438 | 0.47996 | 2.2987 | 0.56615 |
| 1.1591 | 0.54495 | 1.2169 | 0.48552 | 2.4772 | 0.57899 |
| 1.2765 | 0.54741 | 1.3786 | 0.4926 | 2.6695 | 0.59542 |
| 1.4036 | 0.5488 | 1.4793 | 0.49723 | 2.8987 | 0.61954 |
| 1.5289 | 0.55078 | 1.6018 | 0.50161 | 3.1631 | 0.64743 |
| 1.6448 | 0.55215 | 1.7208 | 0.5061 | 3.4235 | 0.67201 |
| 1.7704 | 0.55441 | 1.8527 | 0.50989 | 3.69 | 0.69459 |
| 1.889 | 0.5559 | 1.9517 | 0.51076 | 3.9406 | 0.71248 |
| 1.9965 | 0.55763 | 2.0448 | 0.50644 | 4.168 | 0.72621 |
| 2.1271 | 0.55444 | 2.1241 | 0.50022 | 4.3987 | 0.73975 |
| 2.2105 | 0.55033 | 2.2113 | 0.49595 | 4.6161 | 0.75115 |
| 2.2965 | 0.54423 | 2.3144 | 0.49746 | 4.8312 | 0.76168 |
| 2.3842 | 0.53747 | 2.4937 | 0.5112 | 5.0639 | 0.77205 |
| 2.4709 | 0.53373 | 2.6532 | 0.52471 | 5.2969 | 0.78377 |
| 2.5893 | 0.53581 | 2.8139 | 0.53482 | 5.5271 | 0.79354 |
| 2.7821 | 0.55021 | 2.9821 | 0.54423 | 5.7547 | 0.80282 |
| 2.9619 | 0.56357 | 3.141 | 0.55163 | 5.9629 | 0.81 |
| 3.1289 | 0.5716 | 3.2944 | 0.55903 | 6.1961 | 0.81581 |
| 3.308 | 0.58214 | 3.4517 | 0.56568 | 6.4337 | 0.82265 |
| 3.478 | 0.59324 | 3.6174 | 0.57186 | 6.6327 | 0.82839 |
| 3.6709 | 0.60329 | 3.7798 | 0.57794 | 6.8565 | 0.83267 |
| 3.8466 | 0.61139 | 3.9486 | 0.58507 | 7.0723 | 0.83709 |
| 4.0346 | 0.61941 | 4.2243 | 0.60665 | 7.2703 | 0.84032 |
| 4.2082 | 0.62602 | 4.5596 | 0.63541 | 7.4636 | 0.84452 |
| 4.3993 | 0.63403 | 4.8545 | 0.65739 | 7.6613 | 0.84741 |
| 4.7591 | 0.6671 | 5.1522 | 0.67802 | 7.8693 | 0.84991 |
| 5.1942 | 0.70905 | 5.4315 | 0.69567 | 8.0608 | 0.8526 |
| 5.6734 | 0.75111 | 5.6627 | 0.70909 | 8.2726 | 0.85493 |
| 6.1121 | 0.78725 | 5.9093 | 0.72044 | 8.4818 | 0.8564 |
| 6.466 | 0.80929 | 6.1535 | 0.72996 | 8.6851 | 0.85915 |
| 6.777 | 0.82931 | 6.3798 | 0.73868 | 8.8964 | 0.86241 |
| 7.0678 | 0.84375 | 6.5903 | 0.74618 | 9.1015 | 0.86479 |
| 7.354 | 0.85561 | 6.8113 | 0.75308 | 9.3235 | 0.86867 |
| 7.6187 | 0.86484 | 6.9855 | 0.75943 | 9.5672 | 0.87391 |
| 7.8624 | 0.8735 | 7.2159 | 0.76354 | 9.7841 | 0.87775 |
| 8.1131 | 0.87948 | 7.4417 | 0.76921 | 9.9841 | 0.88085 |
| 8.3427 | 0.88597 | 7.6315 | 0.77305 | 10.2094 | 0.8827 |
| 8.5916 | 0.89033 | 7.8461 | 0.77776 | 10.4176 | 0.8853 |
| 8.831 | 0.89577 | 8.0445 | 0.78198 | 10.6117 | 0.88664 |
| 9.0512 | 0.90179 | 8.2438 | 0.78571 | 10.8181 | 0.88854 |
| 9.2942 | 0.90647 | 8.4471 | 0.78979 | 11.0207 | 0.89019 |
| 9.5361 | 0.91051 | 8.6706 | 0.79352 | 11.2465 | 0.89136 |
| 9.7819 | 0.91492 | 8.853 | 0.7972 | 11.4335 | 0.89249 |
| 10.0034 | 0.91964 | 9.0742 | 0.79989 | 11.6286 | 0.89265 |
| 10.2514 | 0.92343 | 9.2694 | 0.80248 | 11.8163 | 0.89275 |
| 10.4709 | 0.92659 | 9.4627 | 0.80558 | 11.9956 | 0.89374 |
| 10.7103 | 0.93043 | 9.6719 | 0.80863 | 12.2153 | 0.89469 |
| 10.9412 | 0.93417 | 9.8719 | 0.81133 | 12.4112 | 0.89523 |
| 11.154 | 0.93775 | 10.0581 | 0.81406 | 12.5982 | 0.8956 |
| 11.4085 | 0.94098 | 10.2729 | 0.81653 | 12.7908 | 0.89612 |
| 11.6594 | 0.9441 | 10.4758 | 0.81882 | 12.991 | 0.89714 |
| 11.886 | 0.94673 | 10.6855 | 0.82157 | 13.1925 | 0.898 |
| 12.1269 | 0.94999 | 10.8824 | 0.82396 | 13.3938 | 0.89882 |
| 12.353 | 0.95324 | 11.0816 | 0.8267 |  |  |
| 12.6013 | 0.95603 | 11.2804 | 0.82886 |  |  |
| 12.8325 | 0.95948 | 11.4852 | 0.83082 |  |  |
| 13.0867 | 0.96198 | 11.685 | 0.8328 |  |  |
| 13.3205 | 0.9653 | 11.9029 | 0.8349 |  |  |
| 13.5775 | 0.96848 | 12.0942 | 0.83718 |  |  |
| 13.7982 | 0.97108 | 12.2962 | 0.83904 |  |  |
| 14.0522 | 0.97387 | 12.5095 | 0.84108 |  |  |
| 14.2995 | 0.9765 |  |  |  |  |
| 14.5259 | 0.97952 |  |  |  |  |

(c) 28d

| 30kPa | | 60kPa | | 90kPa | |
| --- | --- | --- | --- | --- | --- |
| ε_3_ | ε_3/_ε_1_ | ε_3_ | ε_3/_ε_1_ | ε_3_ | ε_3/_ε_1_ |
| 0.4363 | 0.48825 | 0.5563 | 0.38129 | 0.29415 | 0.40473 |
| 0.5683 | 0.50256 | 0.6709 | 0.40273 | 0.45283 | 0.41862 |
| 0.6747 | 0.51168 | 0.7829 | 0.41848 | 0.61151 | 0.42893 |
| 0.8002 | 0.51813 | 0.9011 | 0.43333 | 0.81277 | 0.44639 |
| 0.9181 | 0.52313 | 1.0177 | 0.44441 | 0.94823 | 0.45789 |
| 1.0353 | 0.52722 | 1.1335 | 0.45364 | 1.17271 | 0.47396 |
| 1.146 | 0.53265 | 1.2351 | 0.46005 | 1.30818 | 0.49122 |
| 1.2785 | 0.53651 | 1.3545 | 0.4675 | 1.55588 | 0.50748 |
| 1.3908 | 0.53899 | 1.4543 | 0.46738 | 1.73778 | 0.52355 |
| 1.5053 | 0.54157 | 1.5302 | 0.46034 | 1.93904 | 0.541 |
| 1.6345 | 0.54385 | 1.6057 | 0.45525 | 2.12095 | 0.55945 |
| 1.7432 | 0.54494 | 1.6763 | 0.44963 | 2.39187 | 0.57909 |
| 1.8238 | 0.53641 | 1.7757 | 0.45081 | 2.52733 | 0.59396 |
| 1.901 | 0.52816 | 1.9197 | 0.46011 | 2.81761 | 0.61142 |
| 1.9982 | 0.52085 | 2.0903 | 0.47735 | 3.06531 | 0.62511 |
| 2.089 | 0.51668 | 2.2507 | 0.4912 | 3.24722 | 0.63899 |
| 2.1923 | 0.51607 | 2.3837 | 0.50135 | 3.44848 | 0.65407 |
| 2.3271 | 0.52709 | 2.5578 | 0.51162 | 3.81229 | 0.67827 |
| 2.536 | 0.54268 | 2.7057 | 0.52065 | 4.12579 | 0.69552 |
| 2.6706 | 0.55025 | 2.8691 | 0.53022 | 4.32704 | 0.70941 |
| 2.8268 | 0.55635 | 3.0315 | 0.53979 | 4.62119 | 0.72567 |
| 2.9629 | 0.56174 | 3.2489 | 0.55704 | 4.86889 | 0.74055 |
| 3.1033 | 0.56577 | 3.5484 | 0.58738 | 5.32172 | 0.76495 |
| 3.2522 | 0.56966 | 3.8678 | 0.61924 | 5.7939 | 0.78677 |
| 3.3936 | 0.57402 | 4.1834 | 0.6485 | 6.20029 | 0.80303 |
| 3.5271 | 0.57858 | 4.487 | 0.67376 | 6.51379 | 0.81335 |
| 3.7002 | 0.58496 | 4.777 | 0.69475 | 6.85051 | 0.82941 |
| 3.9657 | 0.60479 | 5.0338 | 0.71148 | 7.30334 | 0.8433 |
| 4.2675 | 0.6311 | 5.2821 | 0.72425 | 7.64006 | 0.85381 |
| 4.5642 | 0.65583 | 5.5149 | 0.73531 | 8.06967 | 0.86532 |
| 4.8493 | 0.67651 | 5.7297 | 0.74455 | 8.42961 | 0.87107 |
| 5.114 | 0.69486 | 5.9341 | 0.75238 | 8.67731 | 0.87801 |
| 5.3941 | 0.71111 | 6.1637 | 0.7601 | 8.99081 | 0.88138 |
| 5.6596 | 0.72612 | 6.3773 | 0.76582 | 9.30818 | 0.88614 |
| 5.9013 | 0.73968 | 6.5908 | 0.77211 | 9.53266 | 0.88952 |
| 6.1596 | 0.75132 | 6.7965 | 0.77754 | 9.71456 | 0.89408 |
| 6.4105 | 0.7632 | 7.0105 | 0.78333 | 10.05128 | 0.89884 |
| 6.678 | 0.7744 | 7.2108 | 0.78734 | 10.41122 | 0.90459 |
| 6.9376 | 0.78483 | 7.4164 | 0.79159 | 10.77117 | 0.91252 |
| 7.1854 | 0.79462 | 7.6225 | 0.7957 | 11.06531 | 0.91729 |
| 7.434 | 0.80388 | 7.8018 | 0.79937 | 11.35946 | 0.92066 |
| 7.6621 | 0.81157 | 8.0261 | 0.8027 | 11.58394 | 0.92641 |
| 7.9179 | 0.81907 | 8.2292 | 0.80618 | 11.89744 | 0.93216 |
| 8.1608 | 0.82557 | 8.4123 | 0.80982 | 12.0329 | 0.93573 |
| 8.4023 | 0.83258 | 8.6282 | 0.81322 | 12.30382 | 0.9391 |
| 8.6243 | 0.83911 | 8.8427 | 0.81594 | 12.59797 | 0.94486 |
| 8.8675 | 0.84592 | 9.0274 | 0.81935 | 12.86889 | 0.94843 |
| 9.1148 | 0.85208 | 9.2246 | 0.82168 | 13.13595 | 0.95299 |
| 9.3624 | 0.85788 | 9.4361 | 0.82354 | 13.38365 | 0.95874 |
| 9.6025 | 0.86337 | 9.631 | 0.82578 | 13.63135 | 0.95874 |
| 9.8417 | 0.86814 | 9.8215 | 0.82797 | 13.83648 | 0.96449 |
| 10.0726 | 0.8733 | 10.0238 | 0.82989 | 14.1074 | 0.96687 |
| 10.3161 | 0.87767 | 10.2225 | 0.83159 | 14.4209 | 0.97263 |
| 10.5381 | 0.88232 | 10.4136 | 0.83337 | 14.73827 | 0.97263 |
| 10.7721 | 0.88573 | 10.6095 | 0.8351 | 15.07499 | 0.97838 |
| 11.0143 | 0.88995 | 10.7934 | 0.8367 | 15.41171 | 0.98294 |
| 11.2424 | 0.89372 | 11.0029 | 0.83851 |  |  |
| 11.4683 | 0.89747 | 11.1959 | 0.84046 |  |  |
| 11.7033 | 0.90035 | 11.3921 | 0.84211 |  |  |
| 11.9337 | 0.90358 | 11.5737 | 0.84347 |  |  |
| 12.145 | 0.90615 | 11.7875 | 0.84423 |  |  |
| 12.3795 | 0.90873 | 11.9801 | 0.84572 |  |  |
| 12.6084 | 0.91107 | 12.1724 | 0.84683 |  |  |
| 12.8207 | 0.91437 | 12.3722 | 0.84819 |  |  |
| 13.066 | 0.91685 | 12.5445 | 0.8495 |  |  |
| 13.2946 | 0.91979 | 12.7461 | 0.84994 |  |  |
| 13.524 | 0.92247 |  |  |  |  |
| 13.7432 | 0.92474 |  |  |  |  |
