# Supplementary material for: Material composition and constitutive model development of red mud-based filler for highway tunnel invert filling applications: A comprehensive study
Source: PLoS One. 2025 Apr 16;20(4):e0321926. doi: 10.1371/journal.pone.0321926 (PMC12002488; doi:10.1371/journal.pone.0321926)
Supplement: S16 Table — Data of “GF line” of RMBF. (DOCX) [file pone.0321926.s016.docx]

Table S16. The "GF line" of RMBF (Fig.21). Data of "GF line" of RMBF.

| 7d | | 14d | | 28d | |
| --- | --- | --- | --- | --- | --- |
| lg(σ_3_/p_a_) | μ_0_ | lg(σ_3_/p_a_) | μ_0_ | lg(σ_3_/p_a_) | μ_0_ |
| -0.52288 | 0.46866 | -0.52288 | 0.42164 | -0.52288 | 0.40374 |
| -0.22185 | 0.55551 | -0.22185 | 0.48584 | -0.22185 | 0.47231 |
| -0.04576 | 0.62537 | -0.04576 | 0.56589 | -0.04576 | 0.47465 |
