# Supplementary material for: Material composition and constitutive model development of red mud-based filler for highway tunnel invert filling applications: A comprehensive study
Source: PLoS One. 2025 Apr 16;20(4):e0321926. doi: 10.1371/journal.pone.0321926 (PMC12002488; doi:10.1371/journal.pone.0321926)
Supplement: S17 Table — Data of material constants of Duncan-Chang model for. (DOCX) [file pone.0321926.s017.docx]

Table S17. Fitting lines for material constants of Duncan-Chang model for RMBF considering Sp (Fig.22). Data of material constants of Duncan-Chang model for RMBF considering Sp.

| K | | n | | R_f_ | | c | |
| --- | --- | --- | --- | --- | --- | --- | --- |
| Age/d | K | Age/d | n | Age/d | R_f_ | Age/d | c |
| 7 | 0.91 | 7 | 0.385 | 7 | 0.659 | 7 | 17.578 |
| 14 | 0.936 | 14 | 0.482 | 14 | 0.679 | 14 | 42.238 |
| 28 | 1.025 | 28 | 0.325 | 28 | 0.709 | 28 | 80.125 |
| φ | | D | | G | | F | |
| Age/d | φ | Age/d | D | Age/d | G | Age/d | F |
| 7 | 24.83 | 7 | 0.032 | 7 | 0.635 | 7 | 0.324 |
| 14 | 18.97 | 14 | 0.037 | 14 | 0.568 | 14 | 0.293 |
| 28 | 13.85 | 28 | 0.041 | 28 | 0.492 | 28 | 0.157 |
