# Supplementary material for: Material composition and constitutive model development of red mud-based filler for highway tunnel invert filling applications: A comprehensive study
Source: PLoS One. 2025 Apr 16;20(4):e0321926. doi: 10.1371/journal.pone.0321926 (PMC12002488; doi:10.1371/journal.pone.0321926)
Supplement: S18 Table — Data of (σ1-σ3)-ε1 curves calculated by Duncan-Chang model for RMBF considering Sp. (DOCX) [file pone.0321926.s018.docx]

Table S18. The (σ1-σ3)-ε1 curves calculated by Duncan-Chang model for RMBF considering Sp (Fig.23). Data of (σ1-σ3)-ε1 curves calculated by Duncan-Chang model for RMBF considering Sp.

(a) 7d

| 30kPa | | | 60kPa | | | 90kPa | | |
| --- | --- | --- | --- | --- | --- | --- | --- | --- |
| ε_1_ | Theoretical value | Experimental value | ε_1_ | Theoretical value | Experimental value | ε_1_ | Theoretical value | Experimental value |
| 0.2011 | 133.21507 | 156.8221 | 0.2182 | 41.33009 | 48.4347 | 0.1972 | 139.27961 | 326.7377 |
| 0.4061 | 693.53915 | 545.8233 | 0.425 | 72.45683 | 117.0024 | 0.4155 | 401.79512 | 949.7297 |
| 0.6147 | 1232.71328 | 964.5913 | 0.6584 | 291.76215 | 355.0367 | 0.6071 | 669.29365 | 1418.855 |
| 0.8159 | 1680.97633 | 1365.265 | 0.8461 | 674.2099 | 668.6667 | 0.8291 | 1436.99397 | 2159.722 |
| 1.0265 | 2108 | 1929.011 | 1.0625 | 1599.26518 | 1232.543 | 1.0208 | 2211.72271 | 2649.728 |
| 1.22 | 2601.18165 | 2323.615 | 1.2712 | 1690.33754 | 1640.912 | 1.2295 | 2744.20221 | 3019.257 |
| 1.4325 | 2931 | 2665.212 | 1.4836 | 2094.2162 | 1952.761 | 1.4363 | 3188.49178 | 3121.762 |
| 1.6564 | 2164 | 2434.871 | 1.6773 | 1918.54729 | 1815.354 | 1.662 | 3694.74681 | 3332.711 |
| 1.8823 | 1896 | 2266.914 | 1.903 | 1594.60218 | 1752.615 | 1.8745 | 3689.40514 | 3273.218 |
| 2.1022 | 1688 | 1998.134 | 2.0814 | 1614.91593 | 1818.893 | 2.0832 | 3730.8948 | 3186.74 |
| 2.2787 | 1441 | 1761.524 | 2.2863 | 1880.59143 | 2005.802 | 2.2995 | 2885.69449 | 2608.461 |
| 2.4969 | 1448 | 1761.599 | 2.5044 | 1898.97657 | 2034.141 | 2.4779 | 2485.74776 | 2253.418 |
| 2.7019 | 1435 | 1731.373 | 2.7189 | 2151.46957 | 2159.336 | 2.6999 | 2103.07275 | 2021.34 |
| 2.9125 | 1413.95409 | 1820.593 | 2.918 | 2328.338 | 2405.247 | 2.9068 | 1766.66248 | 1828.382 |
| 3.125 | 1506.08476 | 1840.739 | 3.1268 | 2645.73459 | 2495.41 | 3.1192 | 1707.50014 | 1725.934 |
| 3.3355 | 1608.69306 | 1859.979 | 3.3506 | 2888.6 | 2626.206 | 3.3317 | 1653.63706 | 1631.305 |
| 3.5366 | 1952.03563 | 2002.567 | 3.565 | 2644.61164 | 2517.142 | 3.5234 | 1609.32533 | 1587.658 |
| 3.7834 | 2177.38996 | 2054.493 | 3.7738 | 1854.24517 | 1794.179 | 3.732 | 1565.41519 | 1514.22 |
| 3.9522 | 2326 | 2253.33 | 3.9882 | 1467.8894 | 1548.477 | 3.9711 | 1520.21522 | 1514.446 |
| 4.1628 | 2616 | 2379.378 | 4.2007 | 1332.91901 | 1431.955 | 4.1475 | 1490.12756 | 1489.714 |
| 4.3791 | 2836 | 2507.972 | 4.4094 | 1264.50307 | 1355.37 | 4.3658 | 1456.43302 | 1422.125 |
| 4.5689 | 3084 | 2607.098 | 4.6238 | 1176.92519 | 1271.44 | 4.565 | 1428.84884 | 1406.944 |
| 4.7927 | 2906 | 2713.727 | 4.8325 | 1146.38181 | 1223.05 | 4.787 | 1401.3663 | 1390.338 |
| 4.9976 | 2631.85824 | 1963.96 | 5.0108 | 1132.9286 | 1191.177 | 4.9805 | 1379.97841 | 1388.651 |
| 5.212 | 1620.08038 | 1620.914 | 5.2272 | 1080 | 1121.496 | 5.1967 | 1358.65314 | 1370.263 |
| 5.4245 | 1178.82074 | 1169.746 | 5.4548 | 1066 | 1096.232 | 5.4111 | 1339.93369 | 1344.885 |
| 5.62 | 1082.24535 | 1108.407 | 5.6616 | 1050 | 1086.439 | 5.6085 | 1324.62303 | 1329.76 |
| 5.8135 | 1053.50362 | 1030.489 | 5.857 | 1053.611 | 1058.798 | 5.8305 | 1309.37341 | 1358.022 |
| 6.0279 | 1039.95105 | 1072.265 | 6.0695 | 1039.64777 | 1036.093 | 6.0468 | 1296.29197 | 1324.206 |
| 6.2346 | 1031.0672 | 988.4766 | 6.2233 | 1029.76008 | 1052.246 | 6.2346 | 1286.18548 | 1282.871 |
| 6.4434 | 1023.00501 | 973.8125 | 6.4755 | 1013.94329 | 1005.583 | 6.4453 | 1276.05545 | 1268.86 |
| 6.6616 | 1014.80251 | 993.2987 | 6.6994 | 1000.31461 | 966.021 | 6.6501 | 1267.26938 | 1266.526 |
| 6.857 | 1007.50728 | 982.9313 | 6.8968 | 988.62138 | 977.322 | 6.8626 | 1259.08968 | 1267.422 |
| 7.0753 | 999.37776 | 969.9158 | 7.113 | 976.16118 | 932.861 | 7.0827 | 1251.45189 | 1288.738 |
| 7.2952 | 991.20257 | 1023.627 | 7.3123 | 964.996 | 947.08 | 7.2895 | 1244.90107 | 1253.599 |
| 7.4982 | 983.66644 | 1005.787 | 7.5171 | 953.84355 | 920.375 | 7.504 | 1238.60584 | 1244.712 |
| 7.7146 | 975.64384 | 988.2377 | 7.7203 | 943.09963 | 919.4552 | 7.6994 | 1233.20361 | 1217.068 |
| 7.9139 | 968.26514 | 984.6534 | 7.9516 | 931.25958 | 910.7917 | 7.9251 | 1227.23515 | 1214.132 |
| 8.1035 | 961.25441 | 971.6887 | 8.13 | 922.4108 | 926.7781 | 8.1263 | 1222.06147 | 1232.289 |
| 8.3369 | 952.63592 | 980.542 | 8.3692 | 910.93354 | 933.111 | 8.3331 | 1216.79997 | 1182.296 |
| 8.54 | 945.14687 | 976.6729 | 8.5759 | 901.373 | 858.1426 | 8.557 | 1211.07933 | 1213.497 |
| 8.7449 | 937.60142 | 979.4103 | 8.7714 | 892.63534 | 870.2026 | 8.7505 | 1206.05191 | 1204.164 |
| 8.9441 | 930.27547 | 941.0792 | 8.9858 | 883.39366 | 850.5466 | 8.9345 | 1201.15488 | 1167.345 |
| 9.1755 | 921.77715 | 940.363 | 9.1925 | 874.82133 | 843.441 | 9.1622 | 1194.88803 | 1215.217 |
| 9.3634 | 914.88576 | 921.6833 | 9.3804 | 867.31613 | 898.0973 | 9.3822 | 1188.57337 | 1244.283 |
| 9.5645 | 907.51953 | 909.1389 | 9.6061 | 858.663 | 838.0986 | 9.5645 | 1183.12462 | 1177.041 |
| 9.7732 | 899.88505 | 888.6478 | 9.8186 | 850.87694 | 828.8518 | 9.7884 | 1176.14764 | 1199.027 |
| 10.0009 | 891.56728 | 887.9634 | 10.0311 | 843.44101 | 823.3415 | 10.0009 | 1169.22977 | 1156.771 |
| 10.2096 | 883.95434 | 873.3112 | 10.2323 | 836.72318 | 866.9999 | 10.2 | 1162.49121 | 1134.085 |
| 10.424 | 876.14418 | 877.6036 | 10.4296 | 830.44038 | 864.9917 | 10.4069 | 1155.24025 | 1121.87 |
| 10.627 | 868.75929 | 855.8958 | 10.6345 | 824.23506 | 809.335 | 10.6061 | 1148.04335 | 1110.011 |
| 10.8149 | 861.93236 | 841.7752 | 10.8489 | 818.09054 | 809.2016 | 10.8356 | 1139.53133 | 1147.783 |
| 11.0274 | 854.22164 | 837.9693 | 11.0558 | 812.49891 | 808.7418 | 11.0558 | 1131.19844 | 1097.344 |
| 11.2304 | 846.86554 | 827.2611 | 11.2815 | 806.7777 | 811.7732 | 11.2323 | 1124.44869 | 1103.343 |
|  |  |  | 11.4713 | 802.27225 | 847.1695 | 11.4617 | 1115.65574 | 1118.975 |
|  |  |  | 11.6799 | 797.64272 | 797.9464 | 11.6459 | 1108.64408 | 1116.081 |
|  |  |  | 11.8981 | 793.16116 | 845.0461 | 11.8773 | 1099.99926 | 1113.198 |
|  |  |  | 12.0186 | 790.84447 | 784.4625 | 12.0841 | 1092.53143 | 1110.81 |
|  |  |  |  |  |  | 12.272 | 1086.05154 | 1087.26 |
|  |  |  |  |  |  | 12.5034 | 1078.60658 | 1091.519 |
|  |  |  |  |  |  | 12.7084 | 1072.63974 | 1097.862 |
|  |  |  |  |  |  | 12.9209 | 1067.22891 | 1083.486 |
|  |  |  |  |  |  | 13.1257 | 1062.90988 | 1092.584 |
|  |  |  |  |  |  | 13.3364 | 1059.5496 | 1073.637 |
|  |  |  |  |  |  | 13.5355 | 1057.54449 | 1042.19 |
|  |  |  |  |  |  | 13.7366 | 1056.84038 | 1047.138 |
|  |  |  |  |  |  | 13.9473 | 1057.71706 | 1049.937 |
|  |  |  |  |  |  | 14.1636 | 1060.55472 | 1037.096 |

(b) 14d

| 30kPa | | | 60kPa | | | 90kPa | | |
| --- | --- | --- | --- | --- | --- | --- | --- | --- |
| ε_1_ | Theoretical value | Experimental value | ε_1_ | Theoretical value | Experimental value | ε_1_ | Theoretical value | Experimental value |
| 0.2562 | 249.82431 | 299.2428 | 0.1877 | 302.96231 | 302.2815 | 0.203 | 205.78498 | 258.3495 |
| 0.4364 | 532.92153 | 600.308 | 0.4041 | 490.52423 | 646.3006 | 0.3946 | 665.12778 | 687.0336 |
| 0.6394 | 1116.46256 | 1100.454 | 0.6128 | 1021.93924 | 1229.138 | 0.592 | 1108.08115 | 1146.917 |
| 0.8557 | 1646.99219 | 1578.364 | 0.8252 | 1276.05868 | 1619.373 | 0.8026 | 1755.48614 | 1732.755 |
| 1.0492 | 2139.95638 | 1979.727 | 1.0189 | 1708.81789 | 1974.299 | 1.0416 | 2362.37457 | 2291.632 |
| 1.2522 | 1394.47806 | 1694.502 | 1.2446 | 1400 | 1603.35 | 1.2105 | 1475.49688 | 1917.004 |
| 1.4761 | 1216.88716 | 1588.243 | 1.423 | 1200 | 1535.121 | 1.4457 | 1998.06487 | 2327.756 |
| 1.6753 | 1200 | 1443.643 | 1.6279 | 1300 | 1571.243 | 1.6431 | 2206.08972 | 2473.852 |
| 1.8936 | 1219.61021 | 1543.471 | 1.846 | 1668.57418 | 1981.183 | 1.8309 | 2421.28307 | 2582.131 |
| 2.1022 | 1316.31613 | 1587.512 | 2.0605 | 1729.52158 | 1933.302 | 2.0776 | 3360 | 3157.833 |
| 2.2824 | 1573.87065 | 1673.777 | 2.2596 | 2323.68535 | 2149.109 | 2.2843 | 3493 | 3176.754 |
| 2.5045 | 2036.25504 | 1813.448 | 2.4684 | 2432 | 2310.782 | 2.5026 | 3371 | 3040.762 |
| 2.7113 | 2232 | 1968.827 | 2.6922 | 2643 | 2462.473 | 2.7075 | 3038 | 2785.726 |
| 2.9333 | 2428 | 2140.241 | 2.9066 | 2988 | 2566.212 | 2.9029 | 2683.70985 | 2395.65 |
| 3.1419 | 2860 | 2263.012 | 3.1154 | 2495 | 2418.891 | 3.1097 | 2176.61654 | 2098.748 |
| 3.3583 | 2507 | 2125.224 | 3.3298 | 2014.9835 | 1640.502 | 3.3185 | 1833.93908 | 1876.288 |
| 3.5537 | 1639.02109 | 1312.562 | 3.5423 | 1229.65596 | 1243.453 | 3.5366 | 1750.46422 | 1718.675 |
| 3.7453 | 1152.53708 | 1066.736 | 3.751 | 1242.88393 | 1217.231 | 3.7549 | 1629.61772 | 1628.075 |
| 3.9731 | 859.55397 | 897.815 | 3.9654 | 1097.21177 | 1068.196 | 3.9635 | 1553.95238 | 1615.035 |
| 4.1836 | 725.86881 | 744.9958 | 4.1741 | 925.26732 | 968.0883 | 4.1703 | 1461.68994 | 1518.77 |
| 4.4094 | 694.34321 | 670.5224 | 4.3524 | 907.54057 | 895.0682 | 4.3695 | 1433.38818 | 1470.85 |
| 4.5916 | 672.1958 | 606.1983 | 4.5688 | 887.28995 | 851.7544 | 4.5669 | 1407.89645 | 1452.062 |
| 4.7964 | 650.61328 | 586.6986 | 4.7964 | 867.48679 | 849.2334 | 4.7831 | 1382.74127 | 1392.677 |
| 5.0147 | 631.24141 | 605.0813 | 5.0032 | 850.8229 | 819.8307 | 4.9823 | 1361.98045 | 1403.942 |
| 5.2291 | 615.62558 | 579.4281 | 5.1986 | 836.24071 | 791.2851 | 5.1892 | 1342.72461 | 1353.932 |
| 5.4207 | 604.33307 | 559.0399 | 5.4111 | 821.66518 | 837.0536 | 5.3922 | 1325.97529 | 1334.586 |
| 5.6446 | 594.08396 | 564.6395 | 5.5649 | 811.94969 | 647.6346 | 5.5857 | 1311.8569 | 1300.688 |
| 5.8362 | 587.63968 | 556.5923 | 5.8171 | 797.53366 | 815.7448 | 5.8191 | 1297.05066 | 1317.61 |
| 6.0696 | 582.42749 | 573.9183 | 6.041 | 786.3129 | 784.3209 | 6.0448 | 1284.85617 | 1296.385 |
| 6.2783 | 579.98389 | 588.2851 | 6.2384 | 777.65097 | 834.3031 | 6.2308 | 1276.23775 | 1258.846 |
| 6.4566 | 579.38093 | 588.3332 | 6.4546 | 769.48748 | 760.9339 | 6.4585 | 1267.27929 | 1296.648 |
| 6.6729 | 580.27747 | 597.9993 | 6.6539 | 763.18764 | 739.6935 | 6.6728 | 1260.28277 | 1270.54 |
| 6.8931 | 582.78429 | 626.8249 | 6.8587 | 757.93874 | 804.8147 | 6.8759 | 1254.78318 | 1221.409 |
| 7.1112 | 586.59928 | 620.6449 | 7.0619 | 753.95781 | 736.7262 | 7.0618 | 1250.59243 | 1231.929 |
| 7.2972 | 590.71018 | 616.9363 | 7.2932 | 750.91371 | 731.5478 | 7.2649 | 1246.80434 | 1213.269 |
| 7.5211 | 596.47231 | 655.571 | 7.4716 | 749.64751 | 719.8544 | 7.4831 | 1243.49831 | 1214.004 |
| 7.7202 | 602.13515 | 693.457 | 7.7108 | 749.4281 | 725.7053 | 7.6785 | 1241.06986 | 1215.192 |
| 7.9308 | 608.45587 | 658.2561 | 7.9175 | 750.60254 | 748.3251 | 7.9005 | 1238.75637 | 1226.715 |
| 8.1319 | 614.60417 | 618.9873 | 8.113 | 752.87702 | 763.1221 | 8.1281 | 1236.69212 | 1251.647 |
| 8.3141 | 620.10177 | 609.3286 | 8.3274 | 756.67199 | 783.2719 | 8.3331 | 1234.93721 | 1231.121 |
| 8.5437 | 626.69634 | 609.2994 | 8.5341 | 761.61884 | 807.6954 | 8.5398 | 1233.11588 | 1191.469 |
| 8.7694 | 632.55146 | 629.5725 | 8.722 | 767.21307 | 796.6335 | 8.7486 | 1231.07274 | 1236.874 |
| 8.9745 | 637.09646 | 624.7972 | 8.9477 | 775.31421 | 784.6365 | 8.9572 | 1228.67568 | 1182.853 |
| 9.185 | 640.76594 | 606.239 | 9.1602 | 784.31965 | 821.6664 | 9.1717 | 1225.67964 | 1177.045 |
| 9.3786 | 643.05789 | 608.5706 | 9.3727 | 794.66167 | 836.0942 | 9.3709 | 1222.27166 | 1122.512 |
| 9.6006 | 644.17508 | 630.5669 | 9.5739 | 805.68559 | 811.2025 | 9.5587 | 1218.38479 | 1168.135 |
| 9.7941 | 643.63388 | 607.0918 | 9.7712 | 817.65941 | 828.1275 | 9.7902 | 1212.52564 | 1155.257 |
| 10.0236 | 640.90925 | 598.1145 | 9.9761 | 831.3141 | 849.5778 | 9.9914 | 1206.3247 | 1169.9 |
| 10.2191 | 636.59764 | 595.2533 | 10.1905 | 846.93232 | 833.6161 | 10.1925 | 1198.95328 | 1184.934 |
| 10.4391 | 629.31697 | 614.8607 | 10.3974 | 863.29422 | 855.3158 | 10.3992 | 1190.01043 | 1112.953 |
| 10.6288 | 620.78362 | 607.4713 | 10.6231 | 882.58782 | 883.047 | 10.6042 | 1179.62873 | 1125.442 |
| 10.8489 | 620.78362 | 623.4812 | 10.8129 | 899.97969 | 866.4523 | 10.8413 | 1165.55299 | 1114.969 |
| 11.0633 | 620.78362 | 629.3671 | 11.0215 | 920.32418 | 881.6311 | 11.0349 | 1152.26618 | 1114.395 |
| 11.2493 | 620.78362 | 627.439 | 11.2397 | 942.98318 | 933.3688 | 11.2512 | 1135.35416 | 1141.001 |
|  |  |  |  |  |  | 11.4599 | 1116.8149 | 1130.347 |
|  |  |  |  |  |  | 11.6459 | 1098.32809 | 1123.355 |
|  |  |  |  |  |  | 11.8772 | 1072.5878 | 1122.92 |
|  |  |  |  |  |  | 12.0878 | 1046.33532 | 1086.735 |
|  |  |  |  |  |  | 12.2908 | 1018.33856 | 1023.821 |
|  |  |  |  |  |  | 12.4977 | 992.08608 | 1040.818 |
|  |  |  |  |  |  | 12.7045 | 965.8336 | 992.1158 |
|  |  |  |  |  |  | 12.9151 | 939.58112 | 995.8989 |
|  |  |  |  |  |  | 13.1257 | 913.32864 | 985.106 |

(c) 28d

| 30kPa | | | 60kPa | | | 90kPa | | |
| --- | --- | --- | --- | --- | --- | --- | --- | --- |
| ε_1_ | Theoretical value | Experimental value | ε_1_ | Theoretical value | Experimental value | ε_1_ | Theoretical value | Experimental value |
| 0.1935 | 139.7429 | 117.6024 | 0.2143 | 789.34062 | 441.9048 | 0.201 | 80.41329 | 142.1022 |
| 0.3946 | 391.05116 | 356.4367 | 0.4268 | 1261.72305 | 959.0404 | 0.4021 | 463.42744 | 576.6523 |
| 0.5939 | 706.82341 | 668.5667 | 0.6298 | 1872.3063 | 1580.079 | 0.6355 | 962.17235 | 1132.911 |
| 0.831 | 1432.40193 | 1231.843 | 0.8309 | 2160.01581 | 2076.149 | 0.8025 | 1704.1096 | 1716.895 |
| 1.0377 | 1869.50212 | 1642.212 | 1.0416 | 2393.88645 | 2522.542 | 1.0263 | 2833.85297 | 2461.915 |
| 1.2427 | 2124.23068 | 1951.761 | 1.275 | 2021.46168 | 2488.052 | 1.2237 | 3056 | 2907.933 |
| 1.4096 | 1614.8361 | 1812.754 | 1.4817 | 1986.98726 | 2563.899 | 1.4476 | 3674 | 3211.493 |
| 1.6677 | 1333.85963 | 1752.415 | 1.6847 | 2010.03906 | 2588.554 | 1.6544 | 3203 | 3097.94 |
| 1.848 | 1474.51991 | 1818.293 | 1.8573 | 2173.85865 | 2711.104 | 1.8707 | 2927 | 2939.365 |
| 2.0756 | 1645.34991 | 2005.302 | 2.1021 | 2610.75877 | 2827.313 | 2.0814 | 2894.98181 | 2725.334 |
| 2.2691 | 1754.52827 | 2033.341 | 2.2995 | 2918 | 2933.705 | 2.2844 | 2379.78907 | 2446.426 |
| 2.4797 | 2004.62424 | 2160.136 | 2.5138 | 3189 | 2918.058 | 2.5043 | 2425.89062 | 2436.393 |
| 2.7036 | 2625.47887 | 2406.247 | 2.7188 | 3032 | 2806.179 | 2.6904 | 2175.97108 | 2277.601 |
| 2.9066 | 2795 | 2496.51 | 2.9351 | 2574.31197 | 2226.466 | 2.9483 | 2480.53309 | 2385.434 |
| 3.0907 | 2912 | 2627.306 | 3.1438 | 1801.72234 | 1767.695 | 3.1249 | 2104.22803 | 2167.502 |
| 3.3202 | 3021 | 2515.942 | 3.3487 | 1498.68108 | 1539.991 | 3.3392 | 2060.60996 | 2109.795 |
| 3.5517 | 1881.38098 | 1787.279 | 3.5536 | 1344.29582 | 1401.17 | 3.5366 | 2021.8205 | 2058.538 |
| 3.7566 | 1497.00931 | 1545.177 | 3.7623 | 1245.74123 | 1302.197 | 3.7452 | 2008.09718 | 2019.611 |
| 3.954 | 1363.47101 | 1432.955 | 3.9786 | 1145.45941 | 1210.674 | 3.954 | 1918.52331 | 1911.235 |
| 4.1627 | 1291.12823 | 1355.27 | 4.1778 | 1117.42837 | 1167.388 | 4.1607 | 1918.55832 | 1952.163 |
| 4.3544 | 1194.58237 | 1272.24 | 4.3959 | 1102.20504 | 1134.111 | 4.3752 | 1853.09255 | 1849.068 |
| 4.5801 | 1162.58849 | 1222.35 | 4.6028 | 1084.81724 | 1105.684 | 4.5839 | 1795.60012 | 1844.128 |
| 4.7889 | 1130.5677 | 1189.177 | 4.7982 | 1077.42945 | 1066.531 | 4.7471 | 1754.46068 | 1772.142 |
| 4.9728 | 1091.64616 | 1121.896 | 4.9898 | 1075.04165 | 1059.455 | 4.9975 | 1697.14098 | 1708.96 |
| 5.193 | 1080.10118 | 1094.032 | 5.2118 | 1076.57582 | 1056.487 | 5.2137 | 1652.67422 | 1716.382 |
| 5.3941 | 1073.06516 | 1085.039 | 5.4301 | 1063.37138 | 1036.97 | 5.4206 | 1613.96304 | 1650.177 |
| 5.618 | 1060.21942 | 1056.498 | 5.6388 | 1051.34682 | 1056.514 | 5.6104 | 1581.39828 | 1540.215 |
| 5.8342 | 1046.12902 | 1033.893 | 5.8437 | 1040.11103 | 1034.55 | 5.8285 | 1547.08221 | 1583.536 |
| 6.0372 | 1032.74692 | 1048.446 | 6.0523 | 1029.25233 | 1062.967 | 6.0258 | 1518.59302 | 1523.341 |
| 6.2422 | 1020.3927 | 1004.083 | 6.2611 | 1018.96922 | 993.3336 | 6.2574 | 1487.89461 | 1487.13 |
| 6.4357 | 1008.13426 | 965.8206 | 6.4717 | 1009.19136 | 981.3033 | 6.449 | 1464.49275 | 1391.978 |
| 6.6615 | 996.77313 | 975.4225 | 6.6823 | 1000.00995 | 1016.832 | 6.6538 | 1441.25457 | 1388.065 |
| 6.8797 | 983.78456 | 931.1614 | 6.8626 | 992.6234 | 975.9506 | 6.8436 | 1421.18715 | 1364.701 |
| 7.0865 | 971.52058 | 945.48 | 7.1016 | 983.50585 | 969.4088 | 7.0466 | 1401.12305 | 1354.656 |
| 7.2725 | 960.16933 | 918.675 | 7.3103 | 976.17246 | 965.0977 | 7.2819 | 1379.47655 | 1328.651 |
| 7.4773 | 950.19467 | 917.8552 | 7.4906 | 970.30859 | 959.9457 | 7.4792 | 1362.50973 | 1354.299 |
| 7.6917 | 939.47853 | 909.1918 | 7.7126 | 963.68904 | 954.5981 | 7.705 | 1344.2645 | 1345.511 |
| 7.908 | 928.57026 | 924.7781 | 7.9402 | 957.59055 | 950.6943 | 7.9042 | 1329.09425 | 1293.73 |
| 8.1167 | 917.89849 | 932.8106 | 8.1205 | 953.25396 | 951.3979 | 8.1224 | 1313.36083 | 1333.14 |
| 8.3311 | 907.93003 | 856.6426 | 8.3292 | 948.78018 | 956.4624 | 8.3348 | 1298.8355 | 1329.16 |
| 8.5285 | 898.03654 | 869.7026 | 8.5607 | 944.50285 | 958.392 | 8.5209 | 1286.68012 | 1275.443 |
| 8.7486 | 889.24857 | 850.0466 | 8.7656 | 941.31823 | 997.9225 | 8.7296 | 1273.61607 | 1301.342 |
| 8.9382 | 879.82439 | 843.041 | 8.9648 | 938.76347 | 925.3562 | 8.9326 | 1261.42448 | 1261.006 |
| 9.1565 | 872.03188 | 897.5973 | 9.1811 | 936.59368 | 943.7734 | 9.1602 | 1248.29315 | 1289.975 |
| 9.3709 | 863.44419 | 836.6986 | 9.3954 | 935.06443 | 939.1102 | 9.3709 | 1236.58732 | 1265.215 |
| 9.5739 | 855.42173 | 828.6518 | 9.5985 | 934.18511 | 924.8019 | 9.5587 | 1226.4792 | 1209.477 |
| 9.7731 | 848.21257 | 824.1415 | 9.8072 | 933.85942 | 935.514 | 9.7712 | 1215.37258 | 1216.677 |
| 9.9932 | 841.51372 | 865.5999 | 10.0026 | 934.08542 | 914.7972 | 9.9875 | 1204.38998 | 1190.714 |
| 10.2018 | 834.55573 | 862.4917 | 10.2246 | 934.96523 | 921.4097 | 10.2018 | 1193.79463 | 1179.967 |
| 10.3974 | 828.40225 | 808.335 | 10.4238 | 936.31884 | 928.7355 | 10.4106 | 1183.71494 | 1163.268 |
| 10.6175 | 823.03181 | 808.5016 | 10.6307 | 938.28973 | 954.882 | 10.6099 | 1174.29453 | 1168.571 |
| 10.8337 | 817.46233 | 807.2418 | 10.8242 | 940.65393 | 926.889 | 10.811 | 1164.96721 | 1158.262 |
| 11.0159 | 812.49155 | 811.7732 | 11.0651 | 944.3009 | 931.6219 | 11.031 | 1154.94693 | 1127.235 |
| 11.2455 | 808.69609 | 845.2695 | 11.2682 | 947.98196 | 948.8654 | 11.2207 | 1146.44504 | 1102.103 |
| 11.4485 | 804.43757 | 796.1464 | 11.4768 | 952.34016 | 950.3435 | 11.4409 | 1136.72009 | 1138.992 |
| 11.6552 | 801.17006 | 842.7461 | 11.6893 | 957.38151 | 979.376 | 11.6515 | 1127.54896 | 1140.094 |
| 11.8563 | 798.33353 | 782.3625 | 11.8696 | 962.13516 | 966.6097 | 11.8582 | 1118.65828 | 1108.568 |
|  |  |  | 12.0992 | 968.82141 | 983.783 | 12.0688 | 1109.70112 | 1113.569 |
|  |  |  |  |  |  | 12.2871 | 1100.5134 | 1123.665 |
|  |  |  |  |  |  | 12.4996 | 1091.65498 | 1069.23 |
|  |  |  |  |  |  | 12.7045 | 1083.18508 | 1112.954 |
|  |  |  |  |  |  | 12.9037 | 1075.01189 | 1058.917 |
|  |  |  |  |  |  | 13.1162 | 1066.35318 | 1034.16 |
|  |  |  |  |  |  | 13.3344 | 1057.52084 | 1076.351 |
|  |  |  |  |  |  | 13.5487 | 1048.89874 | 1070.5 |
|  |  |  |  |  |  | 13.7461 | 1040.9984 | 1084.779 |
|  |  |  |  |  |  | 13.9528 | 1032.76512 | 1024.898 |
