# Supplementary material for: Material composition and constitutive model development of red mud-based filler for highway tunnel invert filling applications: A comprehensive study
Source: PLoS One. 2025 Apr 16;20(4):e0321926. doi: 10.1371/journal.pone.0321926 (PMC12002488; doi:10.1371/journal.pone.0321926)
Supplement: Attachment — Submitted filename: Response to Reviewers.docx [file pone.0321926.s021.docx]

Dear editors and reviewers,

We sincerely appreciate your letter and the reviewers' comments regarding our manuscript entitled "Material composition and constitutive model development of red mud-based filler for highway tunnel invert filling applications: A comprehensive study" (Manuscript Number: PONE-D-25-03386). These comments are invaluable and extremely helpful in revising and improving our manuscript, and they provide significant guidance for our research. We have carefully reviewed the comments and made corrections, which we hope will meet your approval.

The editor's and reviewers' comments are outlined below in italicized font, with specific concerns numbered. Our responses are provided in blue text, and the changes made to the manuscript are highlighted in yellow text. Specific changes in the responses are in bold text.

The following is the original email:

PONE-D-25-03386

Material composition and constitutive model development of red mud-based filler for highway tunnel invert filling applications: A comprehensive study

PLOS ONE

Dear Dr. Rao,

Thank you for submitting your manuscript to PLOS ONE. After careful consideration, we feel that it has merit but does not fully meet PLOS ONE’s publication criteria as it currently stands. Therefore, we invite you to submit a revised version of the manuscript that addresses the points raised during the review process.

==============================

Please submit your revised manuscript by Apr 09 2025 11:59PM. If you will need more time than this to complete your revisions, please reply to this message or contact the journal office at plosone@plos.org. When you're ready to submit your revision, log on to https://www.editorialmanager.com/pone/ and select the 'Submissions Needing Revision' folder to locate your manuscript file.

Please include the following items when submitting your revised manuscript:

A rebuttal letter that responds to each point raised by the academic editor and reviewer(s). You should upload this letter as a separate file labeled 'Response to Reviewers'.

A marked-up copy of your manuscript that highlights changes made to the original version. You should upload this as a separate file labeled 'Revised Manuscript with Track Changes'.

An unmarked version of your revised paper without tracked changes. You should upload this as a separate file labeled 'Manuscript'.

If you would like to make changes to your financial disclosure, please include your updated statement in your cover letter. Guidelines for resubmitting your figure files are available below the reviewer comments at the end of this letter.

If applicable, we recommend that you deposit your laboratory protocols in protocols.io to enhance the reproducibility of your results. Protocols.io assigns your protocol its own identifier (DOI) so that it can be cited independently in the future. For instructions see: https://journals.plos.org/plosone/s/submission-guidelines#loc-laboratory-protocols. Additionally, PLOS ONE offers an option for publishing peer-reviewed Lab Protocol articles, which describe protocols hosted on protocols.io. Read more information on sharing protocols at https://plos.org/protocols?utm_medium=editorial-email&utm_source=authorletters&utm_campaign=protocols.

We look forward to receiving your revised manuscript.

Kind regards,

Fahd Saeed Alakbari, Ph.D.

Academic Editor

PLOS ONE

Journal Requirements:

When submitting your revision, we need you to address these additional requirements.

1. Please ensure that your manuscript meets PLOS ONE's style requirements, including those for file naming. The PLOS ONE style templates can be found at

https://journals.plos.org/plosone/s/file?id=wjVg/PLOSOne_formatting_sample_main_body.pdf and

https://journals.plos.org/plosone/s/file?id=ba62/PLOSOne_formatting_sample_title_authors_affiliations.pdf

2. Please note that PLOS ONE has specific guidelines on code sharing for submissions in which author-generated code underpins the findings in the manuscript. In these cases, we expect all author-generated code to be made available without restrictions upon publication of the work. Please review our guidelines at https://journals.plos.org/plosone/s/materials-and-software-sharing#loc-sharing-code and ensure that your code is shared in a way that follows best practice and facilitates reproducibility and reuse.

3. In your Methods section, please provide additional information regarding the permits you obtained for the work. Please ensure you have included the full name of the authority that approved the field site access and, if no permits were required, a brief statement explaining why.

4. We note that the grant information you provided in the ‘Funding Information’ and ‘Financial Disclosure’ sections do not match.

When you resubmit, please ensure that you provide the correct grant numbers for the awards you received for your study in the ‘Funding Information’ section.

5. Please review your reference list to ensure that it is complete and correct. If you have cited papers that have been retracted, please include the rationale for doing so in the manuscript text, or remove these references and replace them with relevant current references. Any changes to the reference list should be mentioned in the rebuttal letter that accompanies your revised manuscript. If you need to cite a retracted article, indicate the article’s retracted status in the References list and also include a citation and full reference for the retraction notice.

[Note: HTML markup is below. Please do not edit.]

Reviewers' comments:

Reviewer's Responses to Questions

Comments to the Author

1. Is the manuscript technically sound, and do the data support the conclusions?

The manuscript must describe a technically sound piece of scientific research with data that supports the conclusions. Experiments must have been conducted rigorously, with appropriate controls, replication, and sample sizes. The conclusions must be drawn appropriately based on the data presented.

Reviewer #1: Yes

2. Has the statistical analysis been performed appropriately and rigorously?

Reviewer #1: Yes

3. Have the authors made all data underlying the findings in their manuscript fully available?

The PLOS Data policy requires authors to make all data underlying the findings described in their manuscript fully available without restriction, with rare exception (please refer to the Data Availability Statement in the manuscript PDF file). The data should be provided as part of the manuscript or its supporting information, or deposited to a public repository. For example, in addition to summary statistics, the data points behind means, medians and variance measures should be available. If there are restrictions on publicly sharing data—e.g. participant privacy or use of data from a third party—those must be specified.

Reviewer #1: Yes

4. Is the manuscript presented in an intelligible fashion and written in standard English?

PLOS ONE does not copyedit accepted manuscripts, so the language in submitted articles must be clear, correct, and unambiguous. Any typographical or grammatical errors should be corrected at revision, so please note any specific errors here.

Reviewer #1: Yes

5. Review Comments to the Author

Please use the space provided to explain your answers to the questions above. You may also include additional comments for the author, including concerns about dual publication, research ethics, or publication ethics. (Please upload your review as an attachment if it exceeds 20,000 characters)

Reviewer #1: comments given in atthed file

Let's give the sample specifications in a small table

Are the layer thicknesses different from the values in Figure 3? Explain...

Could it be that the rates of increase are mutually confused???

The explanation of the figure is prone to misunderstanding in this way, at least it will be better to understand if the values determined by the ratio are shown on the graph

Can you give the desired min max values in Code 23 in a table?

give the correlation coefficients to the curve fitting equations....

may be given regression corr coef for the models ?

Can you explain what effect groundwater will have on the RBMF fill in the tunnel?

Can we make an estimate of the RMBF recycling tonnes in tunnel design per km?

6. PLOS authors have the option to publish the peer review history of their article (what does this mean?). If published, this will include your full peer review and any attached files.

If you choose “no”, your identity will remain anonymous but your review may still be made public.

Do you want your identity to be public for this peer review? For information about this choice, including consent withdrawal, please see our Privacy Policy.

Reviewer #1: Yes: Dr. Mesut TIĞDEMİR

[NOTE: If reviewer comments were submitted as an attachment file, they will be attached to this email and accessible via the submission site. Please log into your account, locate the manuscript record, and check for the action link "View Attachments". If this link does not appear, there are no attachment files.]

While revising your submission, please upload your figure files to the Preflight Analysis and Conversion Engine (PACE) digital diagnostic tool, https://pacev2.apexcovantage.com/. PACE helps ensure that figures meet PLOS requirements. To use PACE, you must first register as a user. Registration is free. Then, login and navigate to the UPLOAD tab, where you will find detailed instructions on how to use the tool. If you encounter any issues or have any questions when using PACE, please email PLOS at figures@plos.org. Please note that Supporting Information files do not need this step.

**Journal Requirements:**

When submitting your revision, we need you to address these additional requirements.

1. Please ensure that your manuscript meets PLOS ONE's style requirements, including those for file naming. The PLOS ONE style templates can be found at

https://journals.plos.org/plosone/s/file?id=wjVg/PLOSOne_formatting_sample_main_body.pdf and

https://journals.plos.org/plosone/s/file?id=ba62/PLOSOne_formatting_sample_title_authors_affiliations.pdf

Reply:

Thank you for your valuable comment regarding the manuscript formatting. We have reviewed and revised the manuscript to fully comply with PLOS ONE's formatting requirements. Please see the revised manuscript.

2. Please note that PLOS ONE has specific guidelines on code sharing for submissions in which author-generated code underpins the findings in the manuscript. In these cases, we expect all author-generated code to be made available without restrictions upon publication of the work. Please review our guidelines at https://journals.plos.org/plosone/s/materials-and-software-sharing#loc-sharing-code and ensure that your code is shared in a way that follows best practice and facilitates reproducibility and reuse.

Reply:

Thank you for your comment regarding the sharing of author-generated code. We would like to clarify that our manuscript does not include any author-generated code underpinning the findings. The only reference to code in the manuscript pertains to the serial number field used for inserting references via a reference manager. As no code is involved in our work, we trust that this requirement does not apply to our submission.

At the same time, we think that the word "code" may cause ambiguity. After discussion, we decided to replace "code" with "specification", which can more accurately express the meaning of Chinese standards. Please see pages 6 and 9-10 of the revised manuscript.

The specific modifications are as follows:

“The compaction test was conducted following the Highway Geotechnical Test **Specification** (JTG 3430-2020) [60] to determine the maximum dry density and the optimum moisture content of the mixture. The mixture was prepared by the dry soil method, beginning with a moisture content of 18%, increasing by 2% increments. The mixture was thoroughly mixed and then tested after being sealed for 24 hours. The experiment consisted of 7 groups of tests, with a total of 35 specimens (Table 2). The test utilized the heavy II-2 compaction method, involving compaction in three layers.”

“The UCS test was conducted following the Test **Specification** for Stabilized Materials with Inorganic Binders for Highway Engineering (JTG E51-2009) [61]. The specimens were prepared based on the optimum moisture content. To account for compaction effects during construction, three compaction degrees of 90%, 93%, and 96% were selected. The specimens were formed using hydraulic molds and placed in a standard curing box (temperature 20±2°C, humidity above 95%) for 7 days and 28 days. Three specimens were prepared in each group, resulting in a total of 126 specimens (Table 3). The test instrument used was a YYW-11 unconfined pressure instrument, manufactured by Tianjin Gangyuan Testing Instrument Factory, with a loading rate of 3 mm/min.”

“At a composite modified material content of 30% and a compaction degree of 96%, the 28d unconfined compressive strength of MRM reaches 6.22 MPa, which is still lower than the 7.2 MPa compressive strength requirement for invert filling in the **specification** [20]. To address this, soil stabilizers were introduced, with resin polymer as the main component. In this study, two types of stabilizers, Tushengda (TSD) and Suzhou Luxing (SZLX), were used to mix with MRM containing 30% composite modified materials. As shown in Fig. 9, with the addition of 5-fold diluted TSD stabilizer, the 28d unconfined compressive strength of MRM reaches 7.37 MPa, which meets the requirements of the **specification**.”

3. In your Methods section, please provide additional information regarding the permits you obtained for the work. Please ensure you have included the full name of the authority that approved the field site access and, if no permits were required, a brief statement explaining why.

Reply:

Thank you for your comments on our fieldwork permit. We would like to clarify that material acquisition for this study was conducted at a red mud dump site in Qingzhen, Guiyang, Guizhou Province, and no special permit was required to enter the site. And no specific authorization is required to collect samples from the site.

It should be further explained that, because **Editor Maidelyn R. Peregrin** believed that Figure 4 might involve copyright issues during the technical review, although these two photos were taken by us personally on site, we decided to delete Fig.4 to avoid unnecessary disputes.

4. We note that the grant information you provided in the ‘Funding Information’ and ‘Financial Disclosure’ sections do not match.

When you resubmit, please ensure that you provide the correct grant numbers for the awards you received for your study in the ‘Funding Information’ section.

Reply:

Thank you for pointing out the inconsistency between the ‘Funding Information’ and ‘Financial Disclosure’ sections of our manuscript. We apologize for the oversight, which occurred when we were filling out the relevant content in the submission system. We will make the necessary corrections to ensure that both sections are consistent and accurately reflect the funding sources. The revised manuscript will include the corrected information when resubmitted.

5. Please review your reference list to ensure that it is complete and correct. If you have cited papers that have been retracted, please include the rationale for doing so in the manuscript text, or remove these references and replace them with relevant current references. Any changes to the reference list should be mentioned in the rebuttal letter that accompanies your revised manuscript. If you need to cite a retracted article, indicate the article’s retracted status in the References list and also include a citation and full reference for the retraction notice.

Reply:

Thank you for your comment regarding the reference list. We have carefully reviewed all the references cited in the manuscript to ensure that they are complete and accurate. In addition to making the necessary changes, we have also verified the status of each cited article to confirm that none of them have been retracted.

We can confirm that all cited references are valid and relevant to the manuscript. Any changes made to the reference list we will ensure these are clearly noted in the 'Revised Manuscript with Track Changes'.

We made the following changes

1). We deleted the sentence referring to the reference [3] of the original manuscript, and replaced 3. Peiyu G, Hailun L. On-site exchange meeting on green utilization of red mud. China Nonferrous Metals News. 2024 (col. Column). with 3. Liu G, Zhang C, Song A, Xu D, Ma Z. Synergistic effect of mixed red mud and pyrite cinder as oxygen carrier in CH_4_ chemical looping combustion. International Journal of Hydrogen Energy. 2025; 99:26-34. http://doi.org/10.1016/j.ijhydene.2024.12.093. And added the relevant citation sentences.

2). To avoid format errors in news reports, delete 5. Red mud spill damages rice fields in Bac Kan.; 2014. 6. Xin'an Tiemen Town-Red Mud Reservoir Landslide Danger.; 2016. 7. Aluminium Insider. Hindalco Halts Operations at Jharkand alumina refinery after red mud release.; 2019.

3). We have supplemented the Doi number of reference 43.

The above changes can be found in the introduction and references section of the revised manuscript, on pages 2, 5 and 27 of the revised manuscript.

The specific modifications are as follows:

“The accumulation and disposal of red mud pose serious environmental and safety challenges around the world[1] Red mud is an insoluble industrial waste produced as a byproduct during the extraction of alumina from bauxite. For every ton of alumina produced, about 1.5 tons of red mud are generated[2]. **With the rapid development of the aluminum industry, the demand for alumina is increasing, and a large amount of red mud is discharged. As a major producer of alumina, China discharges millions of tons of red mud every year[3].** Due to the high heavy metal content and strong alkalinity of red mud, the current main method of storing it in open-air dams on land is not only expensive to maintain but also prone to safety accidents. In recent years, red mud leakage incidents have been reported in Hungary[4], **Vietnam, China, and India.** These incidents have resulted in serious pollution of water bodies and farmland, and even caused casualties. Although the global average utilization rate of red mud has reached 15%[5], China's comprehensive utilization rate of red mud remains at only 5.69% (Fig. 1), far below the world average. Faced with the significant challenges of red mud accumulation and utilization, China urgently needs to explore more effective strategies for resource utilization.”

“43.	Xiaoming L, Binwen T, Haifeng Y, Emile M. Durability and environmental performance of red mud-gangue based highway pavement base materials. Journal of Engineering Science. 2018; 40(04):438-445. **http://doi.org/10.13374/j.issn2095-9389.2018.04.006**”

**Reviewer #1**: comments given in atthed file

1.Let's give the sample specifications in a small table

Reply:

Thank you for your helpful suggestion to present the sample specifications in a table. In order to make the experimental variable settings clearer, we have added Tables 2 and 3 to the manuscript. These tables now provide detailed information on the sample specifications and experimental variables, as requested.

We hope these additions enhance the clarity of the manuscript, and we appreciate your feedback. Please see pages 6 of the revised manuscript.

The specific modifications are as follows:

“The compaction test was conducted following the Highway Geotechnical Test Specification (JTG 3430-2020)[60] to determine the maximum dry density and the optimum moisture content of the mixture. The mixture was prepared by the dry soil method, beginning with a moisture content of 18%, increasing by 2% increments. The mixture was thoroughly mixed and then tested after being sealed for 24 hours. The experiment consisted of 7 groups of tests, with a total of 35 specimens **(Table 2)**. The test utilized the heavy II-2 compaction method, involving compaction in three layers.

**Table 2 Compaction test plan**

| **Composite modified materials/%** | **0** | **5** | **10** | **15** | **20** | **25** | **30** |
| --- | --- | --- | --- | --- | --- | --- | --- |
| **Maintenance age/d** | **18~26** | | | | **14~22** | | **12~20** |
| **Number of test pieces** | **20** | | | | **10** | | **5** |
| **Total** | **35** | | | | | | |

The UCS test was conducted following the Test Specification for Stabilized Materials with Inorganic Binders for Highway Engineering (JTG E51-2009)[61]. The specimens were prepared based on the optimum moisture content. To account for compaction effects during construction, three compaction degrees of 90%, 93%, and 96% were selected. The specimens were formed using hydraulic molds and placed in a standard curing box (temperature 20±2°C, humidity above 95%) for 7 days and 28 days. Three specimens were prepared in each group, resulting in a total of 126 specimens **(Table 3)**. The test instrument used was a YYW-11 unconfined pressure instrument, manufactured by Tianjin Gangyuan Testing Instrument Factory, with a loading rate of 3 mm/min.”

**Table 3 Unconfined compressive strength test plan**

| **Composite modified materials/%** | **0** | **5** | **10** | **15** | **20** | **25** | **30** |
| --- | --- | --- | --- | --- | --- | --- | --- |
| **Maintenance age/d** | **7, 28** | | | | | | |
| **Compaction/%** | **90, 93, 96** | | | | | | |
| **Number of test pieces** | **126** | | | | | | |

2.Are the layer thicknesses different from the values in Figure 3? Explain...

Reply:

Thank you for your thoughtful comment. We apologize for the confusion. Upon review, we realized that the RMBF filling diagram in the introduction does not correspond to the sizes used for our confining pressure calculation. Fig. 2 is a schematic diagram showing the tunnel invert filling layer, not the tunnel dimensions used to calculate the confining pressure. Our confining pressure calculation is based on Table 4. To avoid any further ambiguity, we will remove the marked sizes from Fig. 2. Please see page 5 of the revised manuscript.

The specific modifications are as follows:

**Fig. 2** Schematic diagram of RMBF backfill inverted arch

3.Could it be that the rates of increase are mutually confused???

The explanation of the figure is prone to misunderstanding in this way, at least it will be better to understand if the values determined by the ratio are shown on the graph

Reply:

Thank you for pointing out the problem. We did combine the 7-day and 28-day test specimens when analyzing Figure 8, which would cause ambiguity. Now, we have restated the analysis and discussion of Figure 8 to distinguish the analysis content of the two groups of test specimens. At the same time, we have partially processed Figure 8 and annotated the data of the curve graph. We believe that the above changes can make this content clearer and more complete. Please see pages 9 of the revised manuscript.

The specific modifications are as follows:

“**As shown in Fig. 8, the unconfined compressive strength of the modified red mud (MRM) shows a significant increase. For specimens at a curing age of 7 days, the strength increase ranged from 197% to 226%, with an average increase of 210% when the composite modifier was initially added. As the amount of composite modifier continued to increase, the strength gain tended to stabilize, with an increase of 14% to 45%, and an average increase of 23.5%, showing a gradually decreasing trend. For specimens at a curing age of 28 days, the trend was consistent with that of the 7-day specimens. When the composite modifier content was 5%, the average increase reached 233%. As the content continued to increase, the average increase decreased to 23.4%.**”

|  |  |
| --- | --- |
| (a) 7d | (b) 28d |
| **Fig. 8** The changing trend of unconfined compressive strength of MRM | |

4.Can you give the desired min max values in Code 23 in a table?

Reply:

Thank you for your question. According to Specification 20, the strength grade of the invert filling material must not be lower than that of C15 concrete or rubble concrete (the same description can be found on page 3 of the revised manuscript). In China, the design value for the axial compressive strength of C15 concrete is 7.2 MPa, which can be understood as the minimum strength for the invert filling material.

Regarding the maximum strength, the specification does not provide a clear upper limit. But in practice, it is usually sufficient for the material to meet the design strength in actual construction, because increasing the strength often requires the use of more high-strength materials, which means an increase in cost.

The relevant statements are shown in bold below:

“The most effective approach to recycling red mud lies in the field of engineering materials[6]. Many researchers have studied the potential of red mud for a broad spectrum of applications, including its feasibility as a raw material for concrete, mortar, building blocks, tiles, road subgrade, and more[7-17]. Due to the high alkalinity and heavy metal contamination of red mud, it must undergo modification. We have compiled commonly used modification materials from related studies in both Chinese and international literature, primarily including cement, fly ash, slag, metal slag, lime, and others (Table 1). The results indicate that adding these materials improves the strength and other mechanical properties of the mixture to varying extents. Notably, Chen et al.[18] suggested using red mud to replace part of the loess in roadbed construction. Through a series of experiments, it was observed that when the red mud content was 15%-20%, the mechanical properties of the loess roadbed were optimal, exhibiting high compressive strength and good dynamic properties. Similarly, based on previous research conducted by our group[19], we have proposed a novel utilization approach: modifying red mud for use as a filling material for highway tunnel inverts (Fig. 2). **Concerning tunnel invert filling materials, the following regulations are applicable[20]: For tunnels with inverts, the invert filling layer can serve as the roadbed, and the strength grade of the invert filling material should not be lower than C15 concrete or rubble concrete.**”

5.give the correlation coefficients to the curve fitting equations....

Reply:

Thank you for the reviewer's valuable comments. The correlation coefficient (R²) of the curve fitting equation is an important indicator to measure the degree of fit between the data and the fitting curve. We have annotated the R² values of all fitted curves in Fig. 17. Please see page 16-17 of the revised manuscript for details.

The specific modifications are as follows:

| (a) 7d-30kPa | (b) 7d-60kPa | (c) 7d-90kPa |
| --- | --- | --- |

| (d) 14d-30kPa | (e) 14d-60kPa | (f) 14d-90kPa |
| --- | --- | --- |

| (g) 28d-30kPa | (h) 28d-60kPa | (i) 28d-90kPa |
| --- | --- | --- |

Fig. 17 The "*ab* line" of RMBF considering *S_p_*

6.may be given regression corr coef for the models?

Reply:

Thank you for your valuable comment. Figure 23 presents the experimental data and theoretical curves, but it does not involve nonlinear data fitting. We understand that the way the experimental data is presented might give the impression of a fitted curve because the data points are not connected using a linear line type. To avoid any misunderstanding, we will change the line type of the experimental data to better clarify that it is not a fitted curve. Please see page 21 of the revised manuscript.

The specific modifications are as follows:

| (a) 7d | (b) 14d | (c) 28d |
| --- | --- | --- |

Fig. 23 The (*σ*_1_-*σ*_3_)-*ε*_1_ curves calculated by Duncan-Chang model for RMBF considering *S_p_*

7.Can you explain what effect groundwater will have on the RBMF fill in the tunnel?

Reply:

Thank you for your insightful question regarding the potential effects of groundwater on the RMBF fill in the tunnel. Based on previous research conducted by our team, the concentration of harmful substances leached from the modified red mud (using cement, steel slag, slaked lime, and EDTA-2Na as composite modifiers) is below the regulatory limit, classifying it as general solid waste and meeting the environmental protection standards for roadbed filling.

However, the pH of the modified red mud remains high and does not meet the sewage discharge concentration limit. As a result, it is crucial to strictly control the application conditions and roadbed structure when using modified red mud for filling. For instance, the bottom of the roadbed should be maintained at least 0.5 meters above the groundwater level or long-term surface water level.

Although our study primarily focuses on tunnel invert filling, and a curing agent was added to meet the strength requirements, we acknowledge the importance of assessing the environmental impact of this material. Our team is actively studying how to mitigate potential environmental risks, including how to design a tunnel drainage system to effectively manage groundwater and whether additional materials can be used to lower the pH value of the RMBF. This would allow safe usage of the material in tunnels located in areas with abundant groundwater or heavy rainfall.

We believe this ongoing research will be published as a paper in the future.

8.Can we make an estimate of the RMBF recycling tonnes in tunnel design per km?

Reply:

Thank you for your excellent question. Based on the current density of RMBF (2.07 t/m^3^), as long as we can estimate the volume of the tunnel filling layer, we can estimate the number of tons to be recycled. As you know, the shape of the tunnel filling layer is usually irregular. Taking Fig. 2 in the manuscript as an example, we use AutoCAD to calculate the cross-sectional area of the tunnel filling layer, as shown in the figure below, and convert the irregular arc into a regular polygon. Through the "Area" command, we get a cross-sectional area of 8.5 m^2^. As shown in the following formula, it is estimated that 8757.6 t of red mud can be recycled per kilometer.

$8.5m^{2}\times1000m\times2.07t/m^{3}\times51.7\%=8757.6t$ (1)

It is worth noting that this recovery amount is not universal due to the different sizes of tunnels, so we did not give a specific recovery amount in the revised draft. At the same time, since the density of RMBF has been clearly given, the amount of red mud recovered can be estimated for the filling layer of tunnel inverts of different sizes. I hope the above answer can answer your question as much as possible.

**Fig.1** Schematic diagram of cross-sectional area calculation of tunnel invert filling layer

In the end, we would like to express our sincere gratitude to **Dr. Mesut TIĞDEMİR** for his valuable contributions and insightful feedback, which have significantly improved the quality of our manuscript. We would also like to extend our appreciation to **Editor Fahd Saeed Alakbari** for his thorough review and constructive suggestions, which greatly enhanced the clarity and overall quality of the work.
